# Exploring antimicrobial interactions between metal ions and quaternary ammonium compounds toward synergistic metallo-antimicrobial formulations

**DOI:** 10.1128/spectrum.01047-24

**Published:** 2024-08-20

**Authors:** Andrii Lekhan, Raymond J. Turner

**Affiliations:** 1 Department of Biological Sciences, University of Calgary, Calgary, Canada; University of Manitoba, Winnipeg, Canada

**Keywords:** antimicrobial mixtures, synergy, checkerboard assay, metal-based antimicrobials, quaternary ammonium compounds, biofilm prevention

## Abstract

Multi-target antimicrobial agents are considered a viable alternative to target-specific antibiotics, resistance to which emerged as a global threat. Used centuries before the discovery of conventional antibiotics, metal(loid)-based antimicrobials (MBAs), which target multiple biomolecules within the bacterial cell, are regaining research interest. However, there is a significant limiting factor—the balance between cost and efficiency. In this article, we utilize a checkerboard assay approach to explore antimicrobial combinations of MBAs with commonly used quaternary ammonium compound (QAC) antiseptics in order to discover novel combinations with more pronounced antimicrobial properties than would be expected from a simple sum of antimicrobial effects of initial components. This phenomenon, called synergy, was herein demonstrated for several mixtures of Al3+with cetyltrimethylammonium bromide (CTAB) and TeO32- with benzalkonium chloride (BAC) and didecyldimethylammonium bromide (DDAB) against planktonic and biofilm growth of *Pseudomonas aeruginosa* ATCC27853. Biofilm growth of *Escherichia coli* ATCC25922 was synergistically inhibited by the Cu2 +and benzalkonium chloride (BAC) mixture. Multiple additive mixtures were identified for both organisms. The current study observed unexpected species and growth state specificities for the synergistic combinations. The benefit of synergistic mixtures will be captured in economy/efficiency optimization for antimicrobial applications in which MBAs and QACs are presently used.

**IMPORTANCE:**

We are entering the antimicrobial resistance era (AMR), where resistance to antibiotics is becoming more and more prevalent. In order to address this issue, various approaches are being explored. In this article, we explore for synergy between two very different antimicrobials, the antiseptic class of quaternary ammonium compounds and antimicrobial metals. These two antimicrobials have very different actions. Considering a OneHealth approach to the problem, finding synergistic mixtures allows for greater efficacy at lower concentrations, which would also address antimicrobial pollution issues.

## INTRODUCTION

Trends in antibiotic resistance development drive increasing concern within the research community (1). The percentage of antibiotic-resistant isolates of human pathogens is increasing, while the numbers of new antibiotics and novel antibiotic classes delivered to the market were decreasing since the “golden era of antibiotics” in the late 50 s (2). If left in the current state, the antibiotic resistance issue, according to the most pessimistic forecasts, may cause up to 10 million human casualties per year by 2050 (3). Once considered an advantage, target specificity of conventional antibiotics appears to be one of the main reasons for rapid resistance evolution in bacteria (4). Emerging draught in the conventional antibiotics pipeline pushes to reimagine the dominant antimicrobial treatment paradigm when human infections are subject to single-antibiotic class administration at a time. Alternative strategies involve combination of target-specific antimicrobial agents into one multitarget drug (5) or development of single antimicrobial agents with multiple cellular targets (6). While the combinational approach had already demonstrated promising results since the early 2000s in treatment of tuberculosis (7), HIV (8), cancer (9), and malaria (10), the single multitarget antimicrobial agents’ field is just starting to receive attention. Prominent examples of such multitarget antimicrobial agents are metal(loid)-based antimicrobials (MBAs) / metalloantibiotics (11). Several metals were in primitive use well before the conventional antibiotics era: metallic silver and copper were used to preserve water and food (12, 13), and some diseases were treated with arsenic and mercury (14, 15). For the past several decades, silver, copper, and zinc have been increasingly used in medicine as wound dressings, drugs, and antimicrobial creams (16–18) and for odor control in textiles such as silver-containing fabrics (19). Despite the long history of use and current applications, mechanistic studies of the antimicrobial action of MBAs are far from providing a complete picture. Current interest in metal ions and metal ion-containing molecules as antimicrobials lies in the idea that they are the multitarget mode of antimicrobial action, in contrary to most conventional antibiotics, which are predominantly single-target. The main modes of action of MBAs include disruption of [Fe-S] clusters, oxidation of thiol groups of proteins and antioxidants, interference with essential metal uptake, substitution of essential metals in metalloproteins, and production of reactive oxygen species (ROS) both directly and indirectly, as reviewed in detail elsewhere (20).

We see two technical limitations for wider use of MBAs—an economical aspect for coinage metals (such as Ag and Cu, which tend to be expensive) and the efficiency aspect for widely accessible metals (such as Al and Zn, which, although inexpensive, demonstrate moderate antimicrobial properties). Both issues could be mitigated by combination with other antimicrobial agents. Earlier, our group had shown that Cu2 +ions provided by a copper salt may cooperate with quaternary ammonium compounds (QACs) against *E. coli*, increasing the final antimicrobial efficiency of the mixture beyond additive expectations (21); similar observations were made by others for cetylpyridinium chloride and silver ions against *Enterococcus faecalis* (22). Such enhancement of antimicrobial effects, when two antimicrobial agents work together better, than would be expected from summing up the activity of individual substances, is called synergy (23). Along with antagonism, synergy is a phenomenon of drug interactions. Thus, beside obvious benefits of the drug combination to combat resistance development, drug interactions may be further used to control the efficiency and/or cost of the final drug composition.

**Fig 1:**
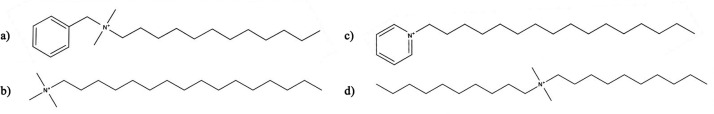
Structural formulas of cations of common aliphatic quaternary ammonium compounds (QACs), [counter-anion] omitted in the figure. (**a**) Benzalkonium [chloride] (BAC); (**b**) cetylpiridinium [bromide] (CPB); (**c**) cetyltrimethylammonium [bromide] (CTAB); (**d**) didecyldimethylammonium [bromide] (DDAB).

In this study, we screened for synergistic effects between several MBAs (Ag+, Cu2+, Zn2+, Al3+, and TeO32-; note: for simplicity of notation, here the relevant metal ion will be referred to rather than the salt or speciation states as typically different salts will give rise to the same antimicrobial outcome) in ionic form and some most common in use aliphatic QAC biocide/antiseptics (benzalkonium, cetylpiridinium, cetyltrimethylammonium, and didecyldimethylammonium) (Fig. 1). We report on results of checkerboard microdilution assays used for determining fractional inhibitory concentration indexes (FICs) and fractional biofilm inhibition indexes (FBICs) for pairs of MBAs and QACs against planktonic and biofilm growth of two well-studied model Gram-negative *Escherichia coli* and *Pseudomonas aeruginosa*—critical pathogens, according to the WHO antibiotic resistance priority list (24). The goal of the study is to find new synergistic mixtures between known antimicrobial metals with known and in use QACs that would have the potential for use in a variety of applications from topical infection treatment or control, touch surface sterilization, and biofouling control.

## RESULTS

A total of 20 combinations of individual MBAs (Ag+, Cu2+, Zn2+, Al3+, and TeO32-) with individual QACs (BAC, CTAB, CPB, and DDAB) were tested against *E. coli* and *P. aeruginosa* for prevention of planktonic and biofilm growth. Thus, 80 unique concentration mixtures were tested, identifying synergistic, additive, indifferent, or antagonistic interactions in the mixtures of MBA/QAC antimicrobials. The checkerboard analyses of each combination for *P. aeruginosa* and *E. coli* are available in Supplementary Material S1 and S2, respectively. Each checkerboard experiment was performed in three biological replicates; FICs and FBICs were first calculated for each, followed by mean and standard deviation calculations. MIC and BPC values were also derived for single antimicrobial agents of interest as a mean value of the 15 replicates for QACs and 12 replicates for MBAs arising from the checkerboard plates (Table 1).

**TABLE 1:** Inhibitory concentrations (minimum inhibitory concentration, MIC; biofilm prevention concentration, BPC) of single antimicrobial agents used in current synergy studies^*a*^

*E. coli*			*P. aeruginosa*	
MBA	MIC (mM)	BPC (mM)	MIC (mM)	BPC (mM)
Ag^2+^	0.053 ± 0.006	0.053 ± 0.006	0.016 ± 0.006	0.020 ± 0.008
Al^3+^	9.375 ± 3.608	13.542 ± 3.608	6.771 ± 1.804	6.250
Cu^2+^	9.375 ± 3.340	7.422 ± 3.340	9.375 ± 3.264	11.364 ± 2.528
Zn^2+^	14.844 ± 4.896	8.239 ± 4.896	15.625 ± 5.653	18.750 ± 6.528
TeO_3_^2-^	0.006 ± 0.001	0.006 ± 0.001	0.050 ± 0.016	0.038 ± 0.021
QAC	MIC (ppm)	BPC (ppm)	MIC (ppm)	BPC (ppm)
BAC	10.000 ± 1.291	9.667 ± 1.291	50.000	51.667 ± 14.840
CPB	12.500 ± 3.169	8.750 ± 3.169	86.667 ± 28.137	100.000
CTAB	12.500 ± 3.165	10.096 ± 3.165	65.208 ± 44.172	63.125 ± 46.748
DDAB	1.854 ± 0.521	1.854 ± 0.521	47.500 ± 44.371	59.167 ± 45.185

^*a*^ Each value represented as mean ± standard deviation is based on at least 12 replicates.

### *P. aeruginosa*—planktonic

**Fig 2:**
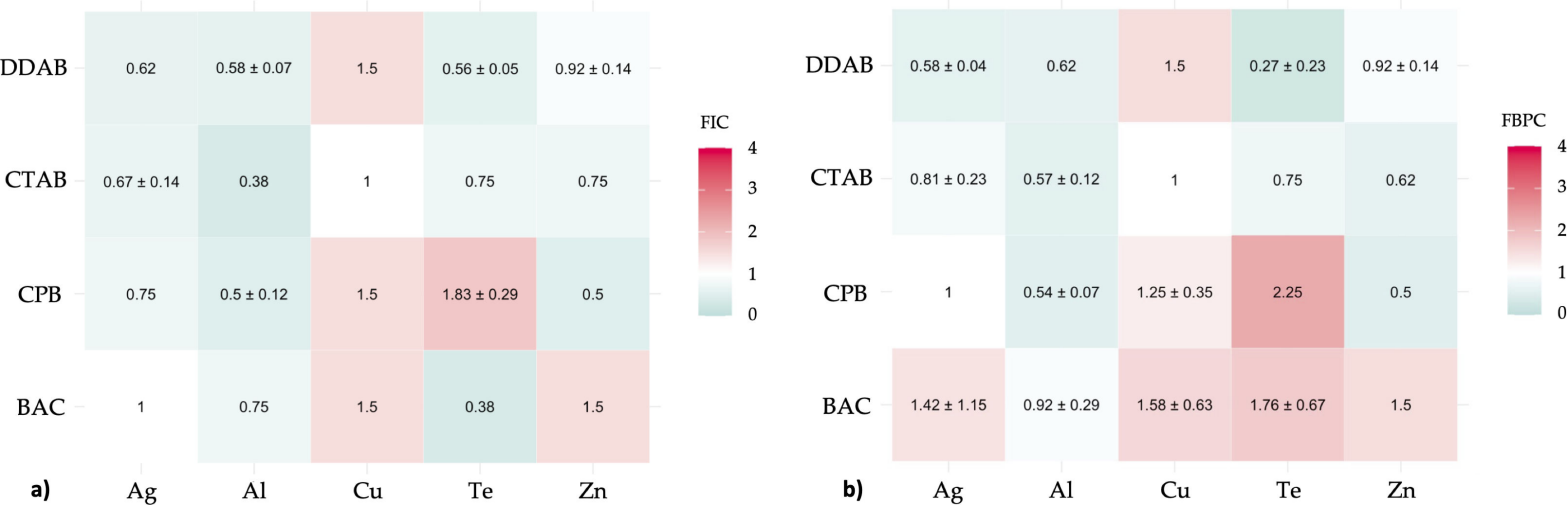
Synergistic properties of studied MBA/QAC mixtures against (a) planktonic growth of *P. aeruginosa* and (b) biofilm growth of *P. aeruginosa*. Each value, represented as mean ± standard deviation, demonstrates the fractional inhibition coefficient and is based on three replicates. A synergistic effect is observed for FIC <0.5 (toward green color) and antagonistic for FIC >4 (toward red color). No antimicrobial agent interaction is observed at FIC = 1 (white color).

None of the studied mixtures were shown to be antagonistic with regard to prevention of planktonic growth of *P. aeruginosa*. he combination of Ag +with all studied QACs is shown to be additive, as well as Al3+/QAC combinations, except for synergy of Al3+/CTAB. Combinations of Cu2 +with CTAB, TeO32- with CTAB, and DDAB were additive, and combination of TeO32- with BAC was synergistic. Zn2 +combinations with selected QACs were mostly additive, except for the indifferent Zn2+/BAC combination (Fig. 2a).

### *P. aeruginosa*—biofilm

Ag+/QAC mixtures were mostly additive against biofilm growth of *P. aeruginosa*, except for the Ag+/BAC mixture that demonstrated indifference. Al3+/QAC mixtures were all additive, and Cu2+/CTAB was borderline additive to indifferent properties. TeO32-/DDAB combinations demonstrated synergistic properties, and TeO32-/CTAB showed additive properties. Zn2+/BAC was indifferent, while other Zn2+/QAC mixtures were additive (Fig. 2b).

### *E. coli*—planktonic

**Fig 3:**
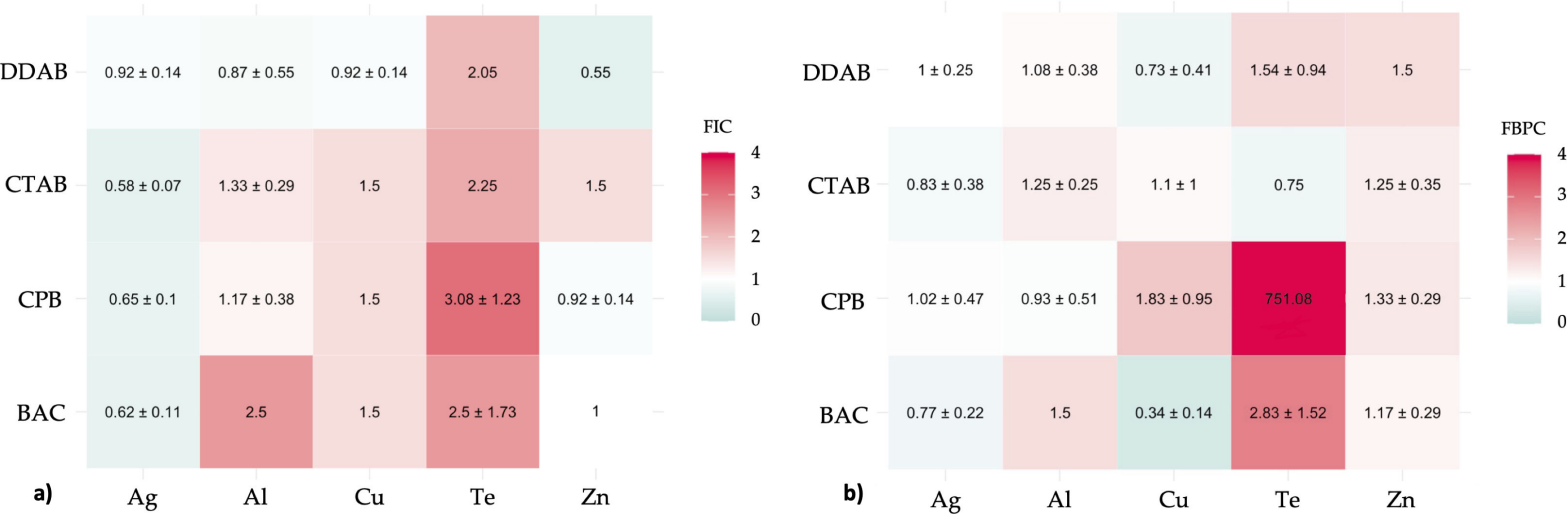
Synergistic properties of studied MBA/QAC mixtures against (a) planktonic growth of *E. coli* and (b) biofilm growth of *E. coli*. Each value, represented as mean ± standard deviation, demonstrates the fractional inhibition coefficient and is based on three replicates. A synergistic effect is observed for FIC <0.5 (toward green color) and antagonistic for FIC >4 (toward red color). No antimicrobial agent interaction is observed at FIC = 1 (white color).

Planktonic growth of *E. coli* was additively prevented by all Ag+/QAC mixtures. Al3+/DDAB was mostly additive. Zn2+/DDAB and Zn2+/CPB were shown to be additive. All other MBA/QAC mixtures were indifferent (Fig. 3a).

### *E. coli*—biofilm

Against biofilm growth of *E. coli*, Ag+/CTAB and Ag+/BAC combinations demonstrated additive antimicrobial properties, similarly to Al3+/CPB. Cu2+/DDAB was shown to be additive. TeO32-/CTAB was shown to be additive, while TeO32-/CPB was dramatically antagonistic. The only synergistic MBA/QAC mixture identified against *E. coli* biofilm growth was Cu2+/BAC (Fig. 3b).

### Best MBA–QAC combinations

Planktonic growth of *P. aeruginosa* was synergistically prevented by the Al3+/CTAB combination in 1.563 mM /1.953 ppm concentrations, respectively, compared to the MICs of single antimicrobial agents of 6.771 ± 1.804 mM / 65.208 ± 44.172 ppm. A similar synergy was demonstrated by the TeO32- /BAC combination at 0.016 mM / 6.25 ppm in contrary to 0.050 ± 0.016 mM / 50 ppm single-agent MICs. The Zn2+/CPB combination demonstrated additivity with fractional concentrations in a mixture of 3.125 mM / 25 ppm, respectively, compared to 15.625 ± 5.653 mM /86.667 ± 28.137 ppm single-agents MICs.

**Fig 4:**
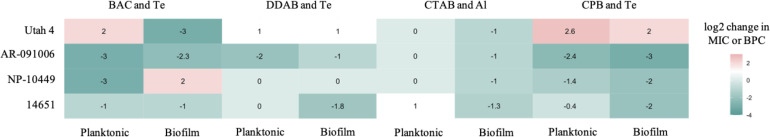
Change in MIC (planktonic) and BPC (biofilm) of synergistic and antagonistic MBA/QAC combinations against clinical isolates of *P. aeruginosa*, compared to *P. aeruginosa* reference strain ATCC 27853. Each value represents a base 2 logarithm of fold-change between clinical isolate MIC (BPC) and indicator strain MIC (BPC). Values are color-coded as green (favorable), white (acceptable), and red (unfavorable) based on antimicrobial activity.

Synergistic prevention of biofilm formation of *P. aeruginosa* was achieved with a mixture of TeO32- /DDAB in respective concentrations of 0.003 mM / 6.25 ppm, while the inhibitory effect with single antimicrobial agents is achieved at concentrations of 0.038 ± 0.021 mM / 59.167 ± 45.185 ppm. The Zn2+/CPB mixture in fractional concentrations of 3.125 mM / 25 ppm demonstrated additive effects, compared to single antimicrobial agents BPCs of 18.750 ± 6.528 mM / 100 ppm. Synergistic combinations of QACs and MBAs (TeO32-/BAC, TeO32-/DDAB, and Al3+/CTAB), as well as the most antagonistic combination (TeO32-/CPB), were tested against several clinical isolates of *P. aeruginosa*. Clinical isolates were as susceptible or more susceptible to synergistic and antagonistic MBA/QAC mixtures, compared to the *P. aeruginosa* reference strain (Fig. 4).

Planktonic growth of *E. coli* was additively prevented with the Zn2+/DDAB mixture in fractional concentrations of 12.5 mM / 0.08 ppm, while respective concentrations of single antimicrobial agents to achieve the same effect were 14.844 ± 4.896 mM / 1.854 ± 0.521 ppm.

**Fig 5:**
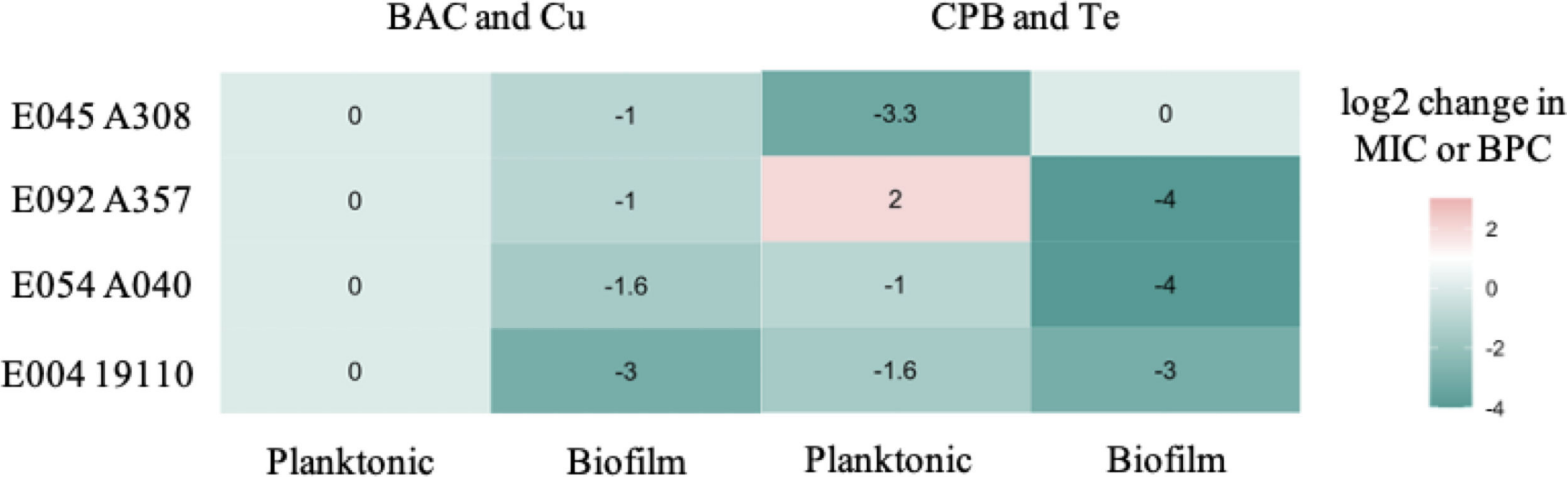
Change in the MIC (planktonic) and BPC (biofilm) of synergistic and antagonistic MBA/QAC combinations against clinical isolates of *E. coli*, compared to the *E. coli* reference strain ATCC 25922. Each value represents a base 2 logarithm of fold-change between clinical isolate MIC (BPC) and indicator strain MIC (BPC). Values are color-coded as green (favorable), white (acceptable), and red (unfavorable) based on antimicrobial activity.

Biofilm growth of *E. coli* was synergistically prevented with the Cu2+ /BAC mixture in 3.125 mM / 0.02 ppm fractional concentrations, compared to single-agent BPCs of 7.422 ± 3.340 mM /9.667 ± 1.291 ppm. Additive biofilm growth prevention was achieved with the TeO32- /CTAB mixture in concentrations of 0.002 mM / 6.25 ppm, while the single-agent’s concentrations required to prevent the biofilm growth are 0.006 ± 0.001 mM / 10.096 ± 3.165 ppm, respectively. The synergistic combination of Cu2+ /BAC and antagonistic combination of TeO32-/CPB were tested against four clinical isolates of *E. coli*, demonstrating, except one instance, the same or greater efficiency, compared to the *E. coli* reference strain of the microorganism (Fig. 5).

## DISCUSSION

Despite promising characteristics of drug cocktails, the field of mechanistic studies of drug–drug interactions is far from having been fully exploited or investigated. An exponential increase in work required to study large amounts of combinations makes direct mechanistic studies problematic for multiple pairwise combinations of agents, not to mention the possible interactions of 3 and more (25). Thus, the current research effort is directed on the study of effects between antimicrobial classes instead of specific drugs to extrapolate information to all the members of the class (26).

Complexity of drug–drug interactions and effects on cells varies from one pair of agents to another. In the simplest case of synergistic effect, one agent could increase the permeability of the cell wall or membrane, increasing the uptake of the companion agent. Agents can also mutually enhance each other’s binding to the same molecular target that leads to more pronounced inhibition. Finally, synergy may be caused by physiological changes within the cell initiated by one agent, which leads to the cell to be more susceptible to another agent in the mixture (27). Antagonism, on the other hand, may originate from two main sources: i) chemical interaction of two agents that inhibit binding with their targets or ii) mutually neutralizing physiological effects achieved by each of the agents (28). Molecular interactions are the most obvious way by which bioactive agents can decrease each other’s efficiency. Reactions and physical interactions between agents can produce less potent products, and spatial or electrostatic binding can decrease both drugs’ bioavailability. Indirect cell physiology-modulated antagonism may also appear, as in the case of bacteriostatic–bactericidal agent mixtures, when bactericidal agent targets growing or dividing cells, but the bacteriostatic agent limits those processes, thus sabotaging the activity of the companion agent (29, 30).

Our study explored antimicrobial synergistic interactions between two groups of antimicrobial agents—QACs and MBAs. QACs are now highly used in a wide variety of applications (31). It is known that aliphatic QACs’ antimicrobial action is predominantly achieved through the interactions with microbial membranes (31). QACs increase membrane permeability, which leads to the uncoupling of the electron transport chain (ETC). Both factors provide potential for synergism with antimicrobial agents that act intracellularly. Increased membrane permeability allows intracellularly acting drugs to enter bacterial cells more readily, increasing their concentration within the cell and thus enhancing antimicrobial properties. This idea is supported with the general trend between *P. aeruginosa* and *E. coli*. The former microorganism demonstrates less ambient membrane permeability, compared to the latter (32), which favors more additivity and synergy between MBAs and membrane-disturbing QACs.

Uncoupled ETC, on the other hand, leads to decrease in proton-motive force (PMF) that provides energy directly or indirectly for the functioning of multidrug efflux pumps that provide resistance to QACs. By reducing the activity of those pumps, aliphatic QACs can decrease the ability of bacterial cells to clear toxic intracellular agents, further increasing their potential to cause damage to the interior of the cell. These concepts are likely to be involved for the synergy cases studied here, including synergistic interactions of multiple aliphatic QACs with Al3+, Zn2 +, and TeO32-. Certainly, further bioinorganic chemical studies are necessary to understand these and why other MBA/QAC combinations were not synergistic.

### *Al* ^3+^

Due to its low intrinsic antimicrobial toxicity, Al3+is rarely referenced as MBAs. However, there is no doubt that the Al3+ion demonstrates antimicrobial effects, especially in mixtures, including natural mixtures such as “medicinal” clays (33). Studies of those mixtures showed that the Al3+ inflict both intracellular damage and membrane damage (34). Inside the cell, Al3+ ions are thought to interfere with normal [Fe-S] cluster functioning, perhaps by mimicking the Fe3 +ion, which leads to malfunctioning of multiple proteins and causes Fe starvation. This effect was clearly shown with the aconitase (Acn) enzyme, demonstrating that Al3+ ions decrease the efficiency of citrate metabolism in bacteria (35). We believe that this mechanism of action, along with few natural ways for Al3+ to enter the cell, contributes the most to QAC/Al3+ synergy patterns. Al3+ is predominantly additive with all classes of QACs against *P. aeruginosa* and is mostly indifferent with QACs against *E. coli*. *P. aeruginosa* testing revealed the synergistic mixture of Al3+ with CTAB. The Al3+/CPB mixture could also be considered slightly synergistic.

Al3+ ions may also interact with membrane phospholipids. Electrostatic interactions lead to aggregation of phosphate groups with further impaired normal phospholipid–protein interactions and membrane protein misfolding (36). It was also suggested that Al3+ may detach the phosphate from phospholipids, as observed by the increased concentration in the growth medium (34). To our belief, this is the reason why not all of the QAC/Al3+mixtures are synergistic. Such a mechanism of action could change the membrane composition to be less favorable toward QAC incorporation, not enough to render this companion agent inactive, but enough to negate the mixture’s potential to be synergistic.

### *Zn* ^2+^

As most other MBAs, Zn2 +primarily targets [Fe-S] clusters and thus inactivates their co-enzymes, such as dehydratases, although its effect is much less pronounced than one of other soft metals, mostly yielding [3Fe-4S] clusters as a product of the reaction, which can be readily fixed by the cell (37). Zn2 +interferes with several bacterial metabolism pathways, such as glycolysis and polysaccharide synthesis. It inhibits glucosyltransferase, glyceraldehyde-3-phosphate dehydrogenase, and pyruvate kinase activities (38).

As a soft acid, Zn2 +ions tend to associate with thiol groups (R-SH) of proteins (39) as well as reduce the bacterial antioxidant potential by thiol depletion of glutathione, glutaredoxin, and thioredoxin systems. The mostly intracellular mechanisms of action of Zn2 +align well with the idea of the increased permeability of the bacterial membranes after exposure to QACs, leading to mixtures being additive to slightly synergistic against *P. aeruginosa*. *E. coli* susceptibility to Zn2 +is similar to *P. aeruginosa* (MICs around 15 mM for both organisms); thus, indifference of Zn2 +mixtures with QACs in terms of synergy is suggested to be due to less exposure of intracellular space of Gram-positive bacteria, compared to Gram-negative after QAC treatment.

### *TeO*_3_^2-^

The tellurium oxyanion, tellurite (TeO32-) has antimicrobial activity that is similar in some respects to that of Ag +in terms of mechanisms, although the TeO32- ion is much more potent, presumably due to a more straightforward uptake system. The oxyanion is to some extent similar to phosphate and thus can be imported inside the cell using the phosphate transporters or via a monocarboxylate importer (40). Within the bacterial cell, TeO32- disrupts thiol homeostasis by depletion of the key antioxidant—glutathione (41). Interactions with other cysteine-containing biomolecules is also a possibility. TeO32- was shown to damage membranes and disrupt proton-motive force creation (42). Under some conditions, TeO32- also indirectly leads to high ROS production by uncoupling the ETC (43) (leading to excess H2O2 levels) and by releasing Fenton-active Fe2 +to the cell—both the result of destruction of [Fe-S] clusters (44). These mechanisms of action for TeO32- were recently validated by our team using transcriptomics and metabolomics approaches (45). One could perhaps expect antagonistic interactions between tellurite and QACs since they present opposite charges and could be electrostatically interacting and as such inactivating both preventing tellurite from entering the cell and QAC from aligning correctly in the membrane. *P. aeruginosa* is less sensitive to TeO32- than *E. coli,* and a slight increase in membrane permeability may cause an increase in TeO32- toxicity through its ability to take electrons from the ETC, even if the effect is partly reduced due to electrostatic interaction between the TeO32- and QAC molecule. Yet this does not explain the selectivity of the TeO32- and QAC combinations where only BAC and DDAB were synergistic.

### *Ag*^+^ and *Cu*^2+^

Ag + and Cu2 +mixtures with QACs demonstrate a range of efficacies from indifferent to additive to synergistic. These MBAs’ molecular targets are both intracellular and membrane (20); therefore, we assume that membrane impairment due to exposure to QACs does not increase the potency of MBAs either because these MBAs “open” the membrane for themselves, or their uptake mechanisms do not rely on the integrity of the membrane. Consistent synergy of BAC and Cu2 +against *E. coli* biofilm growth, along with no other mixture identified as synergistic, remains a difficult phenomenon to explain and more than others requires precise mechanistic research.

As can be seen from the above, mechanistic analysis of drug interactions is complicated. When studying interactions of antimicrobial agents with multiple modes of action, one should consider there is increased space for mechanistic interactions with the companion agent in the mixture. Precise mechanism of synergism/antagonism could frame future synergistic research, theoretically reducing the number of molecules to be tested on practice. It is still unclear and difficult to comprehend how seemingly similar molecules of structure, chemistry, and known antimicrobial mechanisms could raise such different patterns of synergistic interactions. Additionally, those patterns of synergy vary from organism to organism, leading to a yet more demanding task of generalizing the information retrieved from separate studies. The current study just scrapes the surface and demonstrates the width of the landscape of possible synergistic–antagonistic interactions in mixtures, where one or more of the components possesses multiple mechanisms of antimicrobial action.

Regardless, the combinational approach in antimicrobial therapy in general is one of the most obvious and less demanding methods to immediately address antibiotic resistance. To its advantage, the combination of antimicrobial agents may mutually increase their constituents’ properties, resulting in a more effective yet more economically favorable antimicrobial drug, compared to individual agents used. In this study, we identified QAC/MBA synergistic mixtures of CTAB/Al3+, CPB/Zn2+, BAC/ TeO32-, and possibly CPB/Al3+ against planktonic growth of *P. aeruginosa*, while DDAB/ TeO32- and CPB/Zn2 + were synergistic against biofilm growth of *P. aeruginosa*. BAC/Cu2 + was the only mixture demonstrating synergy against biofilm growth of *E. coli*. We also confirmed that these mixtures are at least as effective against antibiotic-resistant clinical isolates of those pathogens as against indicator strains. Future work will aim to understand the remarkable observation of the specificity of the combinations to not only a given bacterial species but also to the growth state of the organism.

QACs and MBAs are already in wide use and approved for applications in agriculture, animal husbandry, cosmetics and personal care products, and human health by infection control. They are also widely used in control of biofouling in the industry, and particularly BAC is a popular biocide to control microbially influenced corrosion. Regardless that the MBAs and QACs used in these studies have had their eukaryotic organism toxicity explored in various ways over the past decades, the combinations here will require specific targeted toxicity experiments performed before use, which are relevant to the applications and how they are used.

## MATERIALS AND METHODS

### Media

Lysogeny broth (LB) agar was prepared in distilled water with 10 g/L NaCl (VWR International Co., Mississauga, Canada), 5 g/L yeast extract (EMD Chemicals Inc., Darmstadt, Germany), 10 g/L tryptone (VWR Chemicals LLC, Solon, USA), and 15 g/L bacteriological agar (VWR International Co., Mississauga, Canada). Mueller–Hinton (MH) broth (HiMedia Laboratories Pvt.Ltd., Mumbai, India) was prepared from powder by dissolving in double-distilled water according to the manufacturer’s recommendations and sterilized by autoclaving.

### Strains

Frozen cultures of *Pseudomonas aeruginosa* ATCC27853 and *Escherichia coli* ATCC25922 were reviewed on lysogeny broth agar plates. A sufficient number of colonies were transferred to saline with a cotton swab to achieve 1.0 McFarland standard turbidity. The bacterial suspension was then diluted 1:30 with MH broth and used as the inoculum. The same approach was later applied to clinical isolates of *P. aeruginosa*: AR-091006, NP-10449, 14651, and Utah 4; and clinical isolates of *E. coli*: E004 19110, E092 A357, E054 A040, and E045 A308; all clinical isolates were resistant to ß-lactam antibiotics, and some isolates also demonstrated resistance to other classes of antibiotics in the clinical environment.

### Stocks

1M crystal violet (CV) (SIGMA-ALDRICH Co., St. Louis, USA) stock was prepared from crystalline form by dissolving in double-distilled water. 1M stocks of selected metal and metalloid salts [AgNO3 (SIGMA Chemical Company, St. Louis, USA), CuSO4 (SIGMA-ALDRICH Co., St. Louis, USA), ZnSO4 (SIGMA-ALDRICH Co., St. Louis, USA), Al2(SO4)3 (MCB, Norwood, USA), and K2TeO3 (VWR International Co., Mississauga, Canada)] were prepared from crystals by dissolving in double-distilled water. 50, 000 ppm stocks of benzalkonium chloride, cetylpiridinium bromide hydrate, and cetyltrimethylammonium (all supplied from SIGMA-ALDRICH Co., St. Louis, USA) were prepared from crystalline form by dissolving in double-distilled water. 50, 000 ppm stock of didecyldimethylammonium bromide (VWR International Co., Mississauga, Canada) was prepared from 80% aqueous gel by diluting with double-distilled water.

### Checkerboard assay

**Fig 6:**
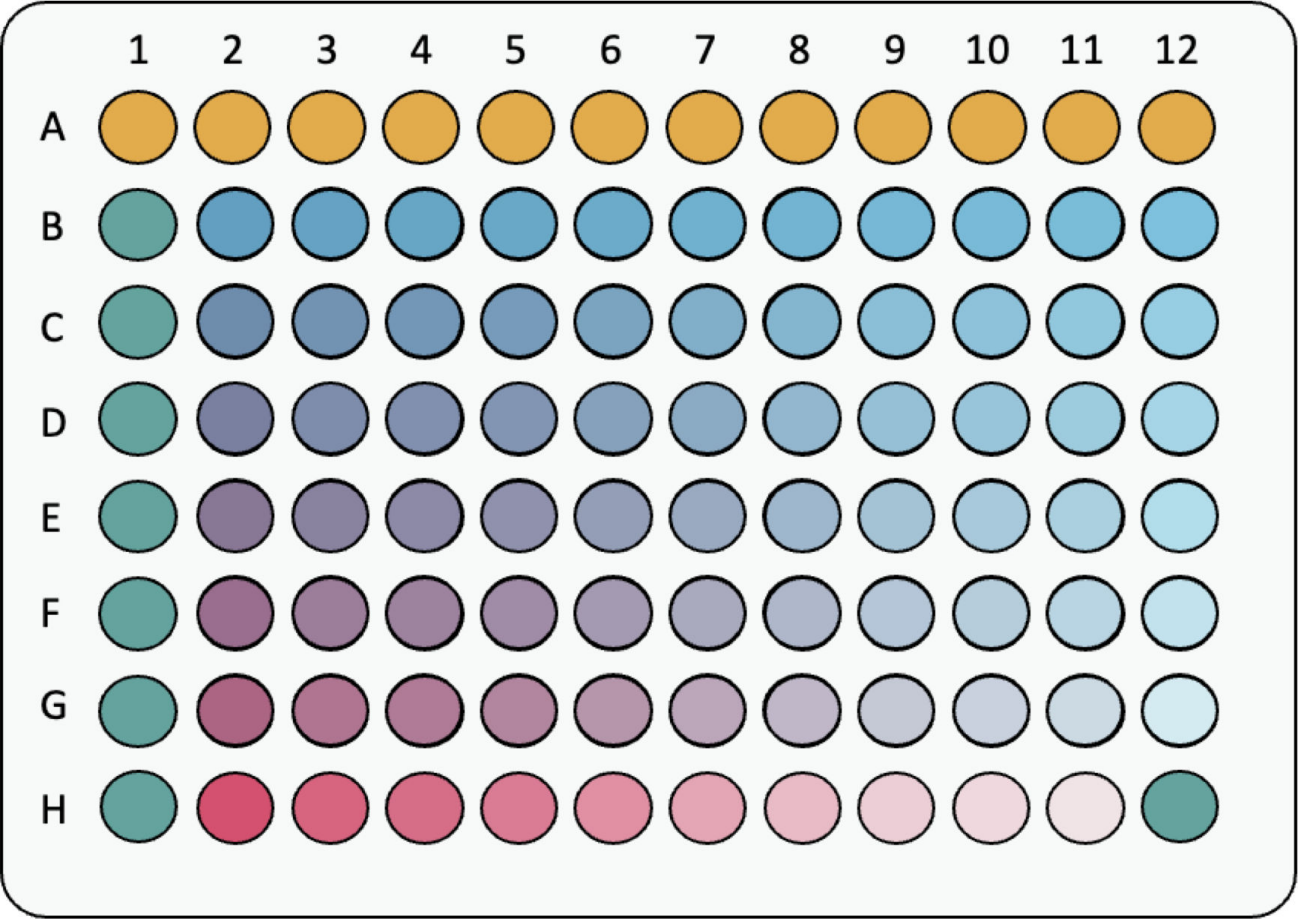
Schematic layout of checkerboard assay. In a microtiter plate, wells A1–A12 are sterility control (growth medium only, yellow-colored), wells B1–H1 are growth control (growth medium and inoculum, green-colored), wells B2–H12 are the challenge zone, where the B2–G11 area contains mixtures of antimicrobials in different concentrations (colored with combination of red and blue), and wells H2–H11 and B12–G12 contain serial dilutions of respective single antimicrobial agents (red or blue gradients, respectively).

For each combination of MBA and QAC, in 96-well microtiter plates (NUNC microwell, ThermoFisher), to wells A1–A12 (first row of the microtiter plate) 150 μL of the growth medium was added to account for sterility control. Wells B1-H1 (first column of the microtiter plate) served as the growth control, containing 135 μL of the growth medium. The checkerboard area of the microtiter plate consists of two orthogonal dilution series and was prepared as follows. All wells in the checkerboard area received 135 μL of the growth medium. To wells B2-B12 (first row of the checkerboard area), 135 uL of MBA was added to reach the desired concentration. Twofold dilution series was prepared from row B to row G of the checkerboard area of the microtiter plate by transferring 135 μL of liquid from the previous row of wells to the next. Twofold dilution series of QAC was prepared similarly for columns 2–11 of checkerboard area. The resulting challenge plate has 60 combinations of two antimicrobials in different concentrations and six serial dilutions of MBA and one serial dilution of QAC alone (Fig. 6). The microtiter plate, except for sterility control wells, was then inoculated with 15 μL of *E. coli* or *P. aeruginosa* inoculum. The plate was then transferred to a shaker-incubator for 24 hours at 37°C and 150 RPM.

Incubated microtiter plates were analyzed using OD reader (PerkinElmer 2030 Multilabel Reader, Victor (TM) X4) at 600 nm wavelength. In single antimicrobial dilution series (last row and last column), wells that have the lowest concentration of the antimicrobial agent that did not allow inoculum proliferation (no change in OD, compared to sterility control) were identified as those that contain the minimum inhibitory concentration (MIC) of that antimicrobial. Within the checkerboard area, the well with no inoculum proliferation identified, containing the least concentration of both antimicrobials, was selected as a reference well. Fractional inhibitory concentration index (FICi) was then calculated as follows:


$$FIC_{i}=\frac{MIC_{AC}}{MIC_{A}}+\frac{MIC_{BC}}{MIC_{B}}$$

where MICA and MICB are MICs of MBA and QAC alone, respectively, and MICAc and MICBc are concentrations of MBA and QAC in the reference well, respectively. FICi values below 0.5 are usually interpreted as a sign of synergistic interactions in the mixture, while mixtures with FICi above 4 are considered antagonistic. Mixtures with FICi in range 0.5–1 and 1–4 are identified as additive and indifferent, respectively (46).

Biofilm data were gathered from incubated MIC plates later, after OD reading. Liquid from the microtiter plate was discarded by inversion and shaking. The plate was rinsed in distilled water by submerging, with water remaining in the plate discarded. About 170 µL 0.1 M crystal violet (CV) solution was added to each well of the rinsed plate. After 10 minutes of exposure, the CV solution was discarded and plates rinsed twice in distilled water by submerging, and remaining rinsing water was discarded. Plates were inverted and tapped on a paper towel to remove unbound dye residues and let dry at room temperature for at least 1 hour. About 30% acetic acid solution was then added to each well, solubilizing possible biofilm and biofilm-associated CV dye. OD600 reading was then taken from the plate. Wells containing the biofilm therefore were stained deep purple, while wells with no biofilm demonstrate little coloration. Biofilm prevention concentrations (BPCs) and reference wells were selected similarly to the MIC. FBPCi calculations followed the same logic as FICi:


$$FBPC_{i}=\frac{BPC_{AC}}{BPC_{A}}+\frac{BPC_{BC}}{BPC_{B}}$$

Further considering variation, during the calculations of FICs or FBPC, the standard deviation of given trials was included to get the possible range of the FIC value. Mixtures were identified as synergistic if the deviation range was completely below or at 0.5 cut-off. If the deviation range contained the cut-off, the mixture was identified as potentially synergistic.

### Clinical isolates

For the combinations that demonstrated synergy (FIC ≤ 0.5) and for the most negatively interacting combination (FIC >2) for each bacterial strain, an additional assay was performed. For each individual mixture, in 96-well microtiter plates (NUNC microwell, ThermoFisher), one column of wells contained 150 μL of the growth medium to account for sterility control, one more column was reserved for growth control, containing 135 μL of the growth medium. Remaining columns contained twofold dilution series of MBA/QAC combination in the growth medium. Microtiter plates were then inoculated with 15 μL inoculum (except sterility control column)—three columns (technical repeats) per strain, including the indicator strain as the reference. MIC and BPC values were visually identified as minimum concentrations that prevented growth of planktonic or biofilm forms of bacterium, respectively, using a similar approach to checkerboard assay. MIC and BPC values for clinical isolates were then transformed using the following approach:


$$\log_{2}\left(\frac{C_{isolate}}{C_{indicator}}\right)$$

where Cisolate and Cindicator are MIC or BPC of the antimicrobial combination against the clinical isolate and indicator strain. After transformation, negative numbers represent decrease in MIC or BPC against the clinical isolate, compared to the indicator strain, while positive numbers represent the increase in the MIC or BPC. Logarithmic nature of the transformed values implies that final numbers represent powers of log2 (e.g., −2 meaning four times lower concentration required and 3 meaning eight times higher concentration required).

## SUPPLEMENTAL MATERIAL

Fig. S1 spectrum.01047-24-s0001.pdf *P. aeruginosa* checkerboard data.

Fig. S2 spectrum.01047-24-s0002.pdf *E. coli* checkerboard data.

ASM does not own the copyrights to Supplemental Material that may be linked to, or accessed through, an article. The authors have granted ASM a non-exclusive, world-wide license to publish the Supplemental Material files. Please contact the corresponding author directly for reuse.

## References

[1] Ventola CL. 2015. The antibiotic resistance crisis: part 1: causes and threats. P T 40:277–283. 25859123 PMC4378521

[2] Bush K, Courvalin P, Dantas G, Davies J, Eisenstein B, Huovinen P, Jacoby GA, Kishony R, Kreiswirth BN, Kutter E, et al.. 2011. Tackling antibiotic resistance. Nat Rev Microbiol 9:894–896. doi:10.1038/nrmicro2693 22048738 PMC4206945

[3] O’Neil J. Review on antimicrobial resistance antimicrobial resistance: tackling a crisis for the healthand wealth of nations. Available from: https://amr-review.org/sites/default/files/AMR%20Review%20Paper%20-%20Tackling%20a%20crisis%20for %20the%20health%20and%20wealth%20of%20nations_1.pdf. Retrieved 7 Dec 2023.

[4] Laxminarayan R, Duse A, Wattal C, Zaidi AKM, Wertheim HFL, Sumpradit N, Vlieghe E, Hara GL, Gould IM, Goossens H, et al.. 2013. Antibiotic resistance-the need for global solutions. Lancet Infect Dis 13:1057–1098. doi:10.1016/S1473-3099(13)70318-9 24252483

[5] Fischbach MA. 2011. Combination therapies for combating antimicrobial resistance. Curr Opin Microbiol 14:519–523. doi:10.1016/j.mib.2011.08.003 21900036 PMC3196371

[6] Gray DA, Wenzel M. 2020. Multitarget approaches against multiresistant superbugs. ACS Infect Dis 6:1346–1365. doi:10.1021/acsinfecdis.0c00001 32156116 PMC7307902

[7] Caminero JA, Sotgiu G, Zumla A, Migliori GB. 2010. Best drug treatment for multidrug-resistant and extensively drug-resistant tuberculosis. Lancet Infect Dis 10:621–629. doi:10.1016/S1473-3099(10)70139-0 20797644

[8] Richman DD. 2001. HIV chemotherapy. Nat New Biol 410:995–1001. doi:10.1038/35073673 11309630

[9] Keith CT, Borisy AA, Stockwell BR. 2005. Multicomponent therapeutics for networked systems. Nat Rev Drug Discov 4:71–78. doi:10.1038/nrd1609 15688074

[10] Malenga G, Palmer A, Staedke S, Kazadi W, Mutabingwa T, Ansah E, Barnes KI, Whitty CJM. 2005. Antimalarial treatment with artemisinin combination therapy in Africa. BMJ 331:706–707. doi:10.1136/bmj.331.7519.706 16195260 PMC1239958

[11] Turner RJ. 2024. The good, the bad, and the ugly of metals as antimicrobials. Biometals 37:545–559. doi:10.1007/s10534-023-00565-y 38112899 PMC11101337

[12] Alexander JW. 2009. History of the medical use of silver. Surg Infect (Larchmt) 10:289–292. doi:10.1089/sur.2008.9941 19566416

[13] Dollwet HH, Sorenson JRJ. 1985. Historic uses of copper compounds in medicine. Trace Elem Med 2:80–87.

[14] Doyle D. 2009. Notoriety to respectability: a short history of arsenic prior to its present day use in haematology. Br J Haematol 145:309–317. doi:10.1111/j.1365-2141.2009.07623.x 19298591

[15] Zhao M, Li Y, Wang Z. 2022. Mercury and mercury-containing preparations: history of use, clinical applications, pharmacology, toxicology, and pharmacokinetics in traditional Chinese medicine. Front Pharmacol 13:807807. doi:10.3389/fphar.2022.807807 35308204 PMC8924441

[16] Woodmansey EJ, Roberts CD. 2018. Appropriate use of dressings containing nanocrystalline silver to support antimicrobial stewardship in wounds. Int Wound J 15:1025–1032. doi:10.1111/iwj.12969 30117675 PMC7949668

[17] Salah I, Parkin IP, Allan E. 2021. Copper as an antimicrobial agent: recent advances. RSC Adv 11:18179–18186. doi:10.1039/d1ra02149d 35480904 PMC9033467

[18] Schwartz JR. 2016. Zinc pyrithione: a topical antimicrobial with complex pharmaceutics. J Drugs Dermatol 15:140–144. 26885780

[19] Lee HJ, Yeo SY, Jeong SH. 2003. Antibacterial effect of nanosized silver colloidal solution on textile fabrics. J Mat Sci 10:2199–2204.

[20] Lemire JA, Harrison JJ, Turner RJ. 2013. Antimicrobial activity of metals: mechanisms, molecular targets and applications. Nat Rev Microbiol 11:371–384. doi:10.1038/nrmicro3028 23669886

[21] Harrison JJ, Turner RJ, Joo DA, Stan MA, Chan CS, Allan ND, Vrionis HA, Olson ME, Ceri H. 2008. Copper and quaternary ammonium cations exert synergistic bactericidal and antibiofilm activity against Pseudomonas aeruginosa. Antimicrob Agents Chemother 52:2870–2881. doi:10.1128/AAC.00203-08 18519726 PMC2493123

[22] Lv S, Fan W, Fan B. 2023. Enhanced in vitro antibacterial effect against Enterococcus faecalis by using both low-dose cetylpyridinium chloride and silver ions. BMC Oral Health 23:299. doi:10.1186/s12903-023-02972-6 37198581 PMC10190056

[23] Greco WR, Bravo G, Parsons JC. 1995. The search for synergy: a critical review from a response surface perspective. Pharmacol Rev 47:331–385. 7568331

[24] WHO Media Centre. WHO publishes list of bacteria for which new antibiotics are urgently needed. Available from: https://www.who.int/en/news-room/detail/27-02-2017-who-publishes-list-of-bacteria-for-which-new-antibiotics-are-urgently-needed. Retrieved 7 Dec 2023.

[25] Berenbaum MC. 1989. What is synergy? Pharmacol Rev 41:93–141. 2692037

[26] Bollenbach T. 2015. Antimicrobial interactions: mechanisms and implications for drug discovery and resistance evolution. Curr Opin Microbiol 27:1–9. doi:10.1016/j.mib.2015.05.008 26042389

[27] Ankomah P, Johnson PJT, Levin BR. 2013. The pharmaco -, population and evolutionary dynamics of multi-drug therapy: experiments with S. aureus and E. coli and computer simulations. PLoS Pathog 9:e1003300. doi:10.1371/journal.ppat.1003300 23593006 PMC3617031

[28] Tyers M, Wright GD. 2019. Drug combinations: a strategy and their potential to combat antibiotic resistance. Nat Rev Microbiol 17:141–145. 30683887 10.1038/s41579-018-0141-x

[29] Singh N, Yeh PJ. 2017. Suppressive drug combinations and their potential to combat antibiotic resistance. J Antibiot (Tokyo) 70:1033–1042. doi:10.1038/ja.2017.102 28874848 PMC5659931

[30] Ocampo PS, Lázár V, Papp B, Arnoldini M, Abel zur Wiesch P, Busa-Fekete R, Fekete G, Pál C, Ackermann M, Bonhoeffer S. 2014. Antagonism between bacteriostatic and bactericidal antibiotics is prevalent. Antimicrob Agents Chemother 58:4573–4582. doi:10.1128/AAC.02463-14 24867991 PMC4135978

[31] Gerba CP. 2015. Quaternary ammonium biocides: efficacy in application. Appl Environ Microbiol 81:464–469. doi:10.1128/AEM.02633-14 25362069 PMC4277564

[32] Hancock REW. 1998. Resistance mechanisms in Pseudomonas aeruginosa and other non fermentative Gram-negative bacteria. Clin Infect Dis 27:S93–S99. 9710677 10.1086/514909

[33] Morrison KD, Misra R, Williams LB. 2016. Unearthing the antibacterial mechanism of medicinal clay: a geochemical approach to combating antibiotic resistance. Sci Rep 6:19043. doi:10.1038/srep19043 26743034 PMC4705759

[34] Londono SC, Hartnett HE, Williams LB. 2017. Antibacterial activity of aluminum in clay from the Colombian amazon. Environ Sci Technol 51:2401–2408. doi:10.1021/acs.est.6b04670 28121138

[35] Middaugh J, Hamel R, Jean-Baptiste G, Beriault R, Chenier D, Appanna VD. 2005. Aluminum triggers decreased aconitase activity via Fe-S cluster disruption and the overexpression of isocitrate dehydrogenase and isocitrate lyase: a metabolic network mediating cellular survival. J Biol Chem 280:3159–3165. doi:10.1074/jbc.M411979200 15548528

[36] Oteiza PI, Vesteraeten SV. 2006. Interactions of Al and related metals with membrane phospholipids: consequences on membrane physical properties. Adv Planar Lipid Bilayers Liposomes 4:79–106. doi:10.1016/S1554-4516(06)04003-8

[37] Xu FF, Imlay JA. 2012. Silver(I), mercury(II), cadmium(II), and zinc(II) target exposed enzymic iron-sulfur clusters when they toxify Escherichia coli. Appl Environ Microbiol 78:3614–3621. doi:10.1128/AEM.07368-11 22344668 PMC3346352

[38] Koo H, Sheng J, Nguyen PTM, Marquis RE. 2006. Co-operative inhibition by fluoride and zinc of glucosyl transferase production and polysaccharide synthesis by mutans streptococci in suspension cultures and biofilms. FEMS Microbiol Lett 254:134–140. doi:10.1111/j.1574-6968.2005.00018.x 16451191

[39] Pace NJ, Weerapana E. 2014. Zinc-binding cysteines: diverse functions and structural motifs. Biomolecules 4:419–434. doi:10.3390/biom4020419 24970223 PMC4101490

[40] Kessi J, Turner RJ, Zannoni D. 2022. Tellurite and Selenite: how can these two oxyanions be chemically different yet so similar in the way they are transformed to their metal forms by bacteria? Biol Res 55:17. doi:10.1186/s40659-022-00378-2 35382884 PMC8981825

[41] Harrison JJ, Tremaroli V, Stan MA, Chan CS, Vacchi-Suzzi C, Heyne BJ, Parsek MR, Ceri H, Turner RJ. 2009. Chromosomal antioxidant genes have metal ion-specific roles as determinants of bacterial metal tolerance. Environ Microbiol 11:2491–2509. doi:10.1111/j.1462-2920.2009.01973.x 19555372

[42] Borghese R, Borsetti F, Foladori P, Ziglio G, Zannoni D. 2004. Effects of the metalloid oxyanion tellurite (TeO32-) on growth characteristics of the phototrophic bacterium Rhodobacter capsulatus. Appl Environ Microbiol 70:6595–6602. doi:10.1128/AEM.70.11.6595-6602.2004 15528523 PMC525167

[43] Lohmeier-Vogel EM, Ung S, Turner RJ. 2004. In vivo 31P-NMR investigation of tellurite toxicity in Escherichia coli. Appl Environ Microbiol 70:7342–7347. doi:10.1128/AEM.70.12.7342-7347.2004 15574934 PMC535159

[44] Pérez JM, Calderón IL, Arenas FA, Fuentes DE, Pradenas GA, Fuentes EL, Sandoval JM, Castro ME, Elías AO, Vásquez CC. 2007. Bacterial toxicity of potassium tellurite: unveiling an ancient enigma. PLoS One 2:e211. doi:10.1371/journal.pone.0000211 17299591 PMC1784070

[45] Pormohammad A, Firrincieli A, Salazar-Alemán DA, Mohammadi M, Hansen D, Cappelletti M, Zannoni D, Zarei M, Turner RJ. 2023. Insights into the synergistic antibacterial activity of silver nitrate with potassium tellurite against Pseudomonas aeruginosa. Microbiol Spectr 11:e0062823. doi:10.1128/spectrum.00628-23 37409940 PMC10433965

[46] Jung WK, Koo HC, Kim KW, Shin S, Kim SH, Park YH. 2008. Antibacterial activity and mechanism of action of the silver ion in Staphylococcus aureus and Escherichia coli. Appl Environ Microbiol 74:2171–2178. doi:10.1128/AEM.02001-07 18245232 PMC2292600

