# Supplementary material for: Exploring antimicrobial interactions between metal ions and quaternary ammonium compounds toward synergistic metallo-antimicrobial formulations
Source: Microbiol Spectr. 2024 Aug 20;12(10):e01047-24. doi: 10.1128/spectrum.01047-24 (PMC11448152; doi:10.1128/spectrum.01047-24)
Supplement: Fig. S1 — P. aeruginosa checkerboard data. [file spectrum.01047-24-s0001.pdf]

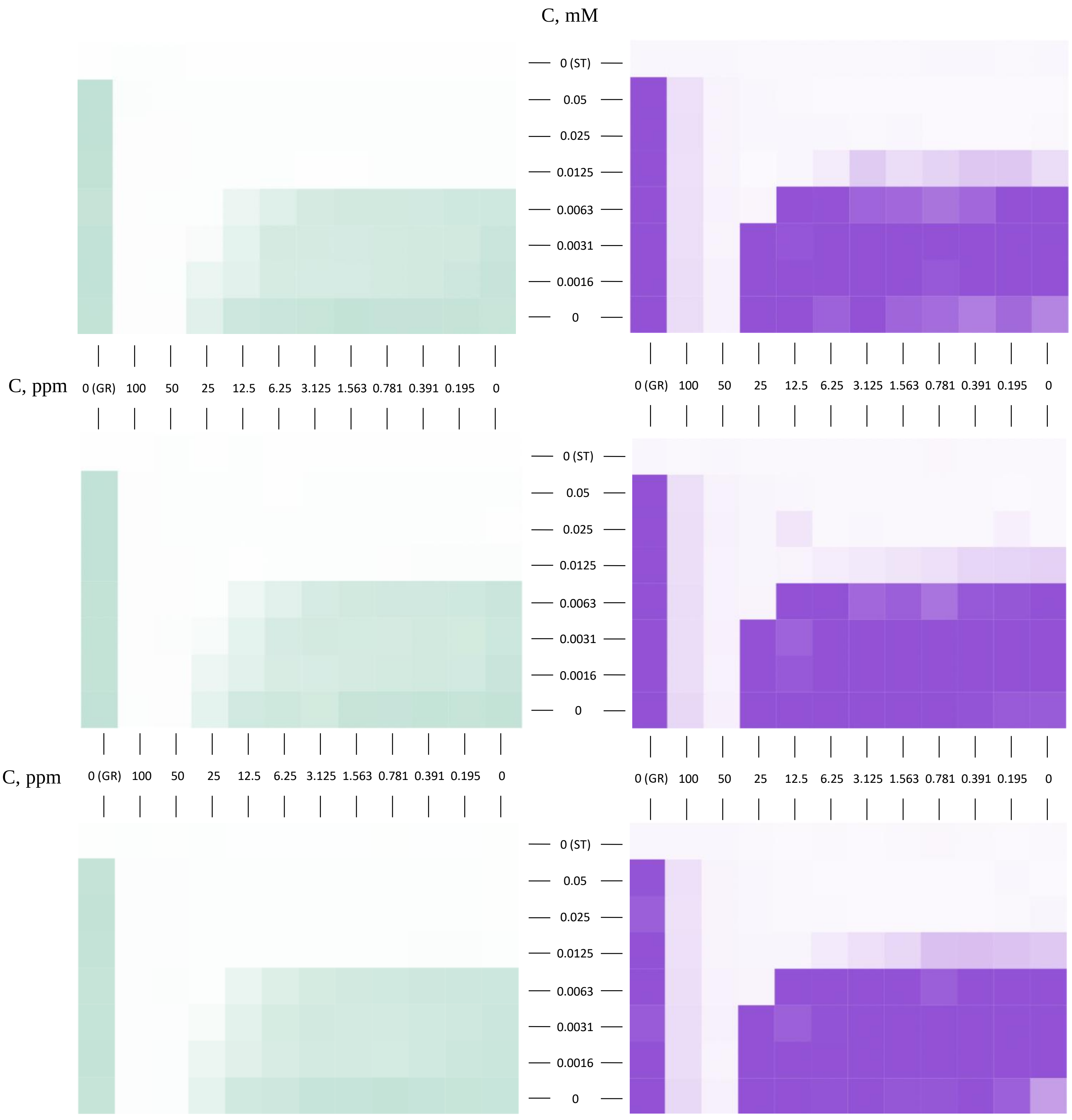

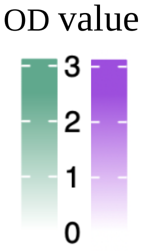

Figure S.1.1. BAC/Ag⁺, *P. aeruginosa*. Heatmaps of OD readings from the grown plates of planktonic (green to white) and biofilm (purple to white) growth of *P. aeruginosa* after 24h exposure to checkerboard assay of benzalkonium chloride (BAC, horizontal concentrations gradient) and silver nitrate (Ag^+, vertical concentrations gradient).

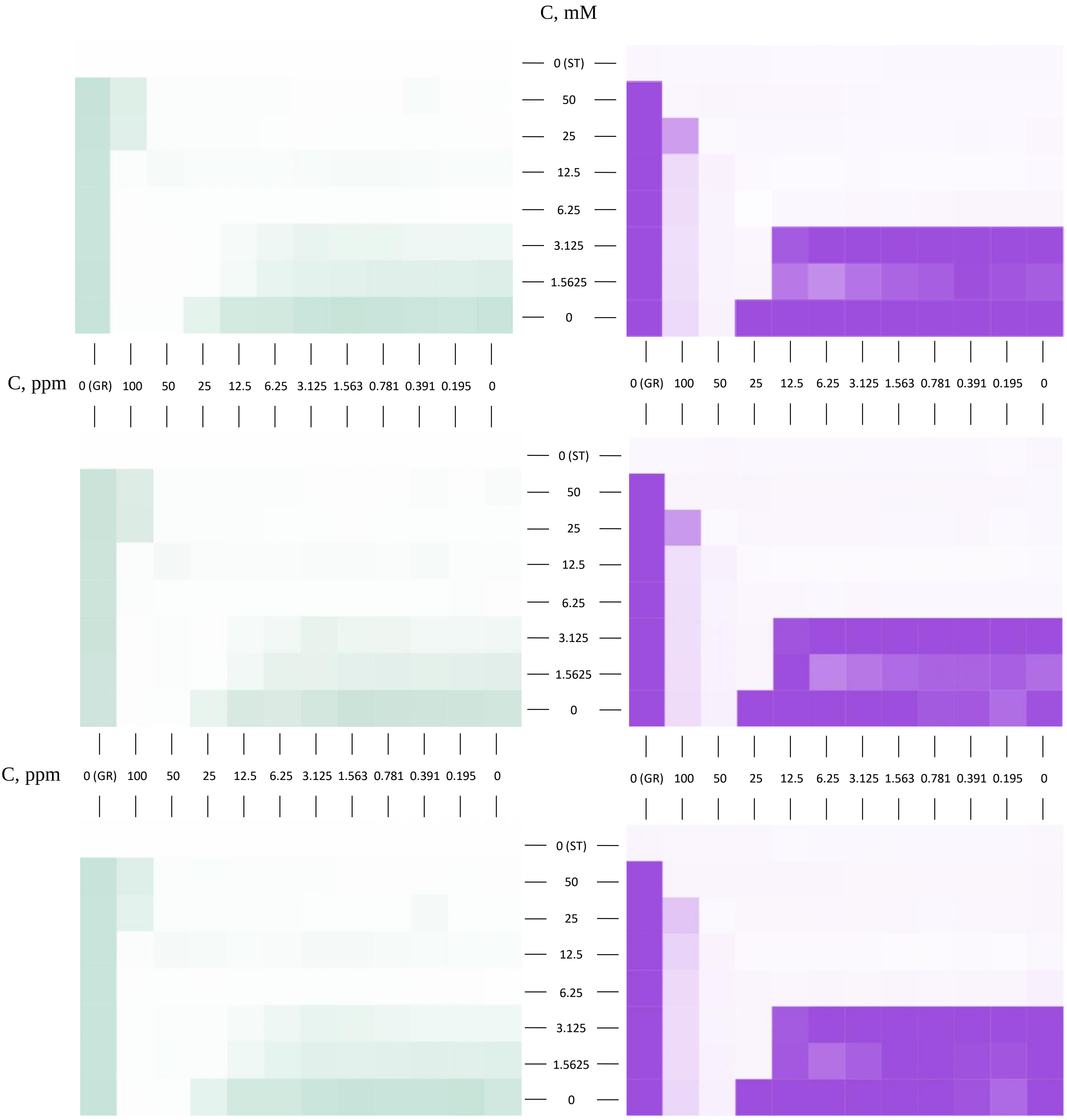

Figure S.1.2. BAC/Al^{3+}, *P. aeruginosa*. Heatmaps of OD readings from the grown plates of planktonic (green to white) and biofilm (purple to white) growth of *P. aeruginosa* after 24h exposure to checkerboard assay of benzalkonium chloride (BAC, horizontal concentrations gradient) and aluminum chloride (Al^{3+}, vertical concentrations gradient).

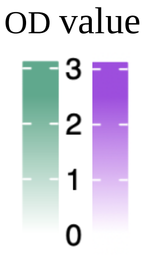

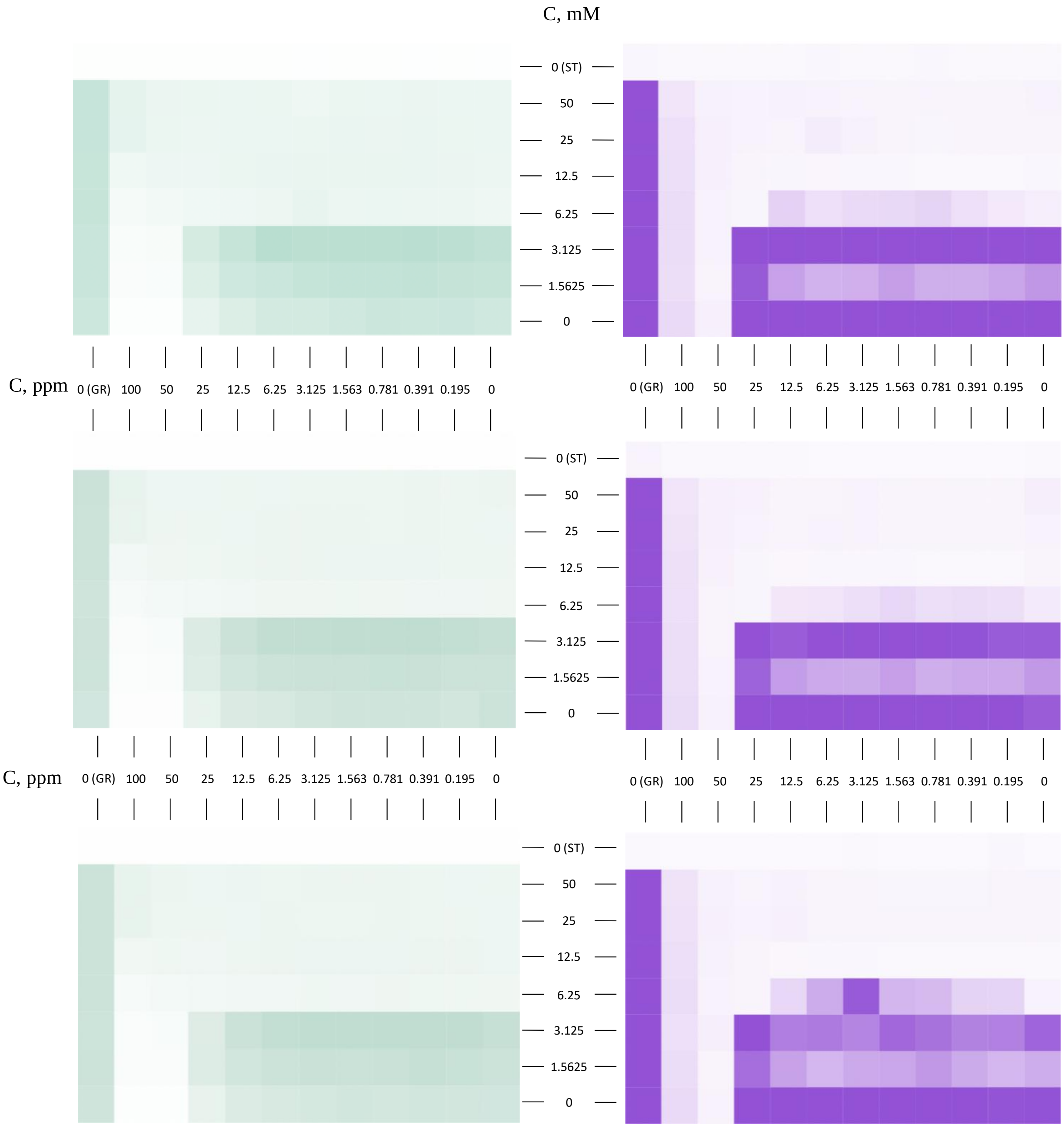

Figure S.1.3. BAC/Cu^{2+}, *P. aeruginosa.* Heatmaps of OD readings from the grown plates of planktonic (green to white) and biofilm (purple to white) growth of *P. aeruginosa* after 24h exposure to checkerboard assay of benzalkonium chloride (BAC, horizontal concentrations gradient) and copper chloride (Cu^{2+}, vertical concentrations gradient).

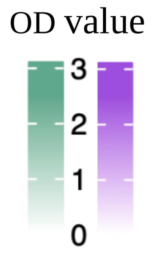

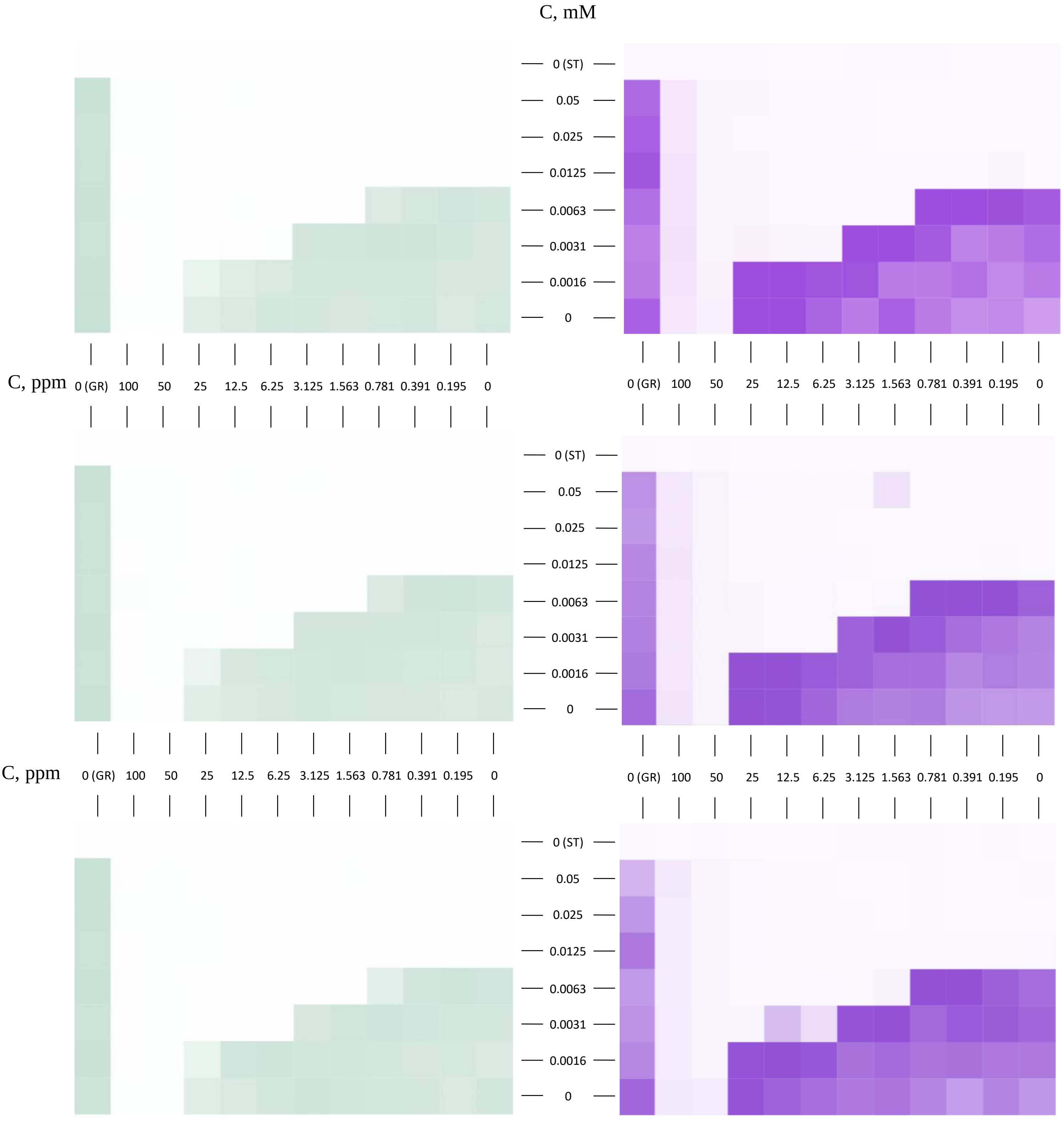

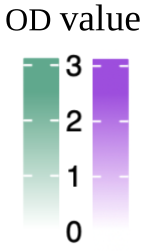

Figure S.1.4. BAC/TeO_3^{2-}, *P. aeruginosa.* Heatmaps of OD readings from the grown plates of planktonic (green to white) and biofilm (purple to white) growth of *P. aeruginosa* after 24h exposure to checkerboard assay of benzalkonium chloride (BAC, horizontal concentrations gradient) and sodium tellurite (TeO_3^{2-}, vertical concentrations gradient).

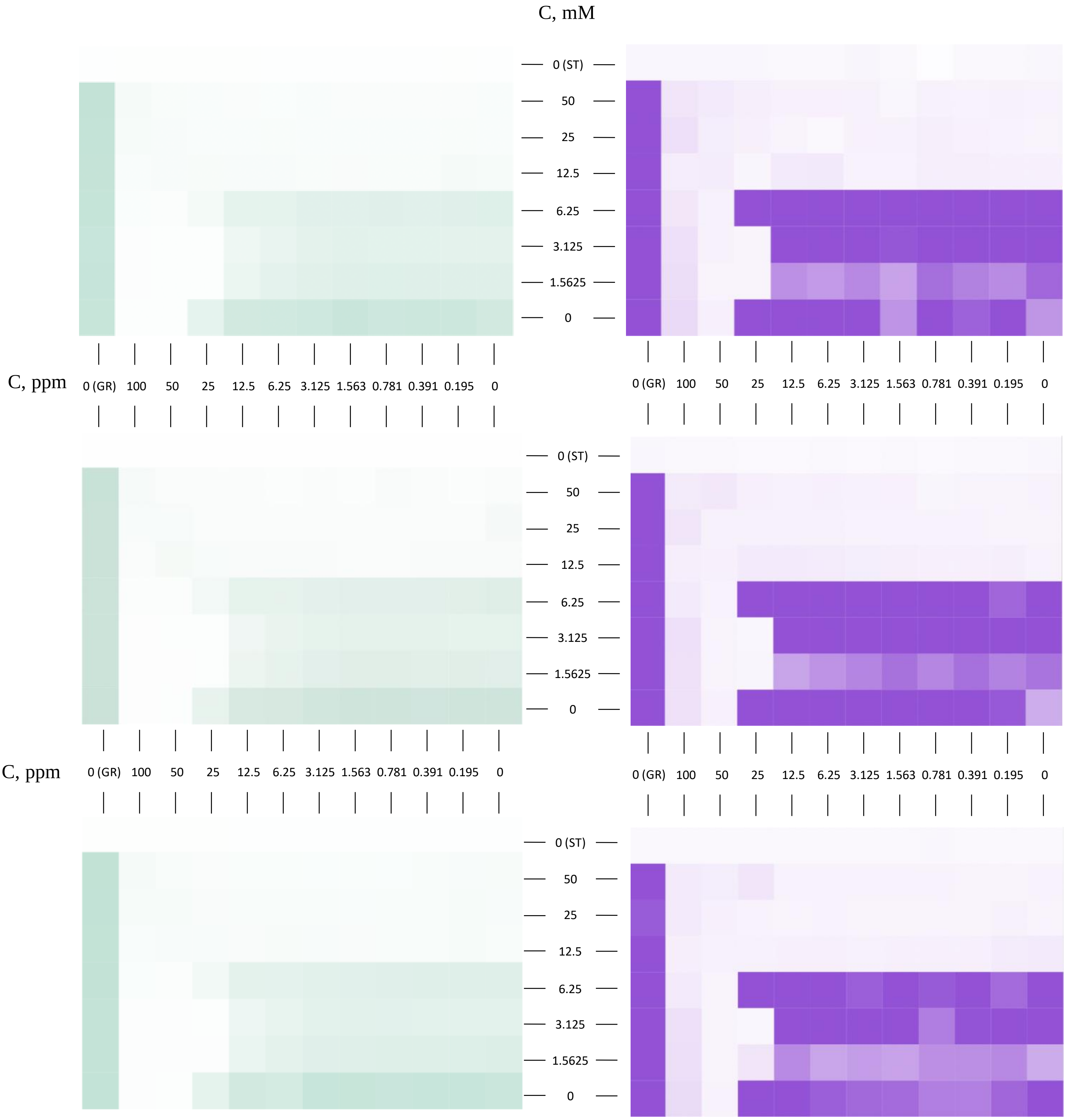

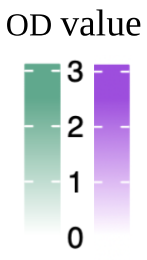

Figure S.1.5. BAC/Zn⁺, *P. aeruginosa.* Heatmaps of OD readings from the grown plates of planktonic (green to white) and biofilm (purple to white) growth of *P. aeruginosa* after 24h exposure to checkerboard assay of benzalkonium chloride (BAC, horizontal concentrations gradient) and zinc chloride (Zn^{2+}, vertical concentrations gradient).

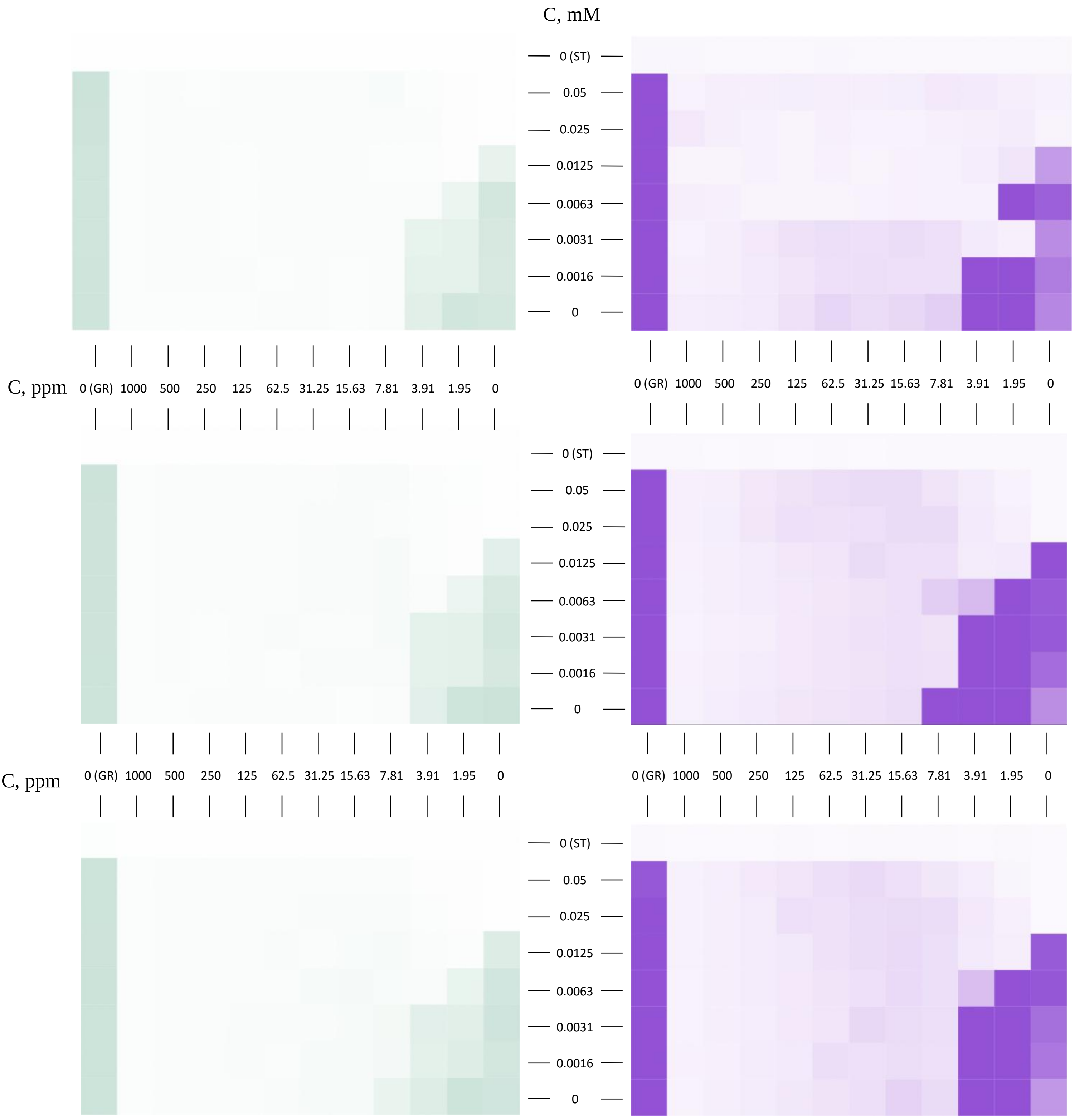

Figure S.1.6. CTAB/Ag⁺, *P. aeruginosa*. Heatmaps of OD readings from the grown plates of planktonic (green to white) and biofilm (purple to white) growth of *P. aeruginosa* after 24h exposure to checkerboard assay of cetyltrimethylammonium bromide (CTAB, horizontal concentrations gradient) and silver nitrate (Ag^+, vertical concentrations gradient).

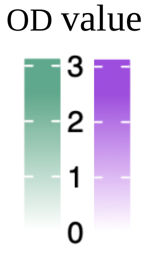

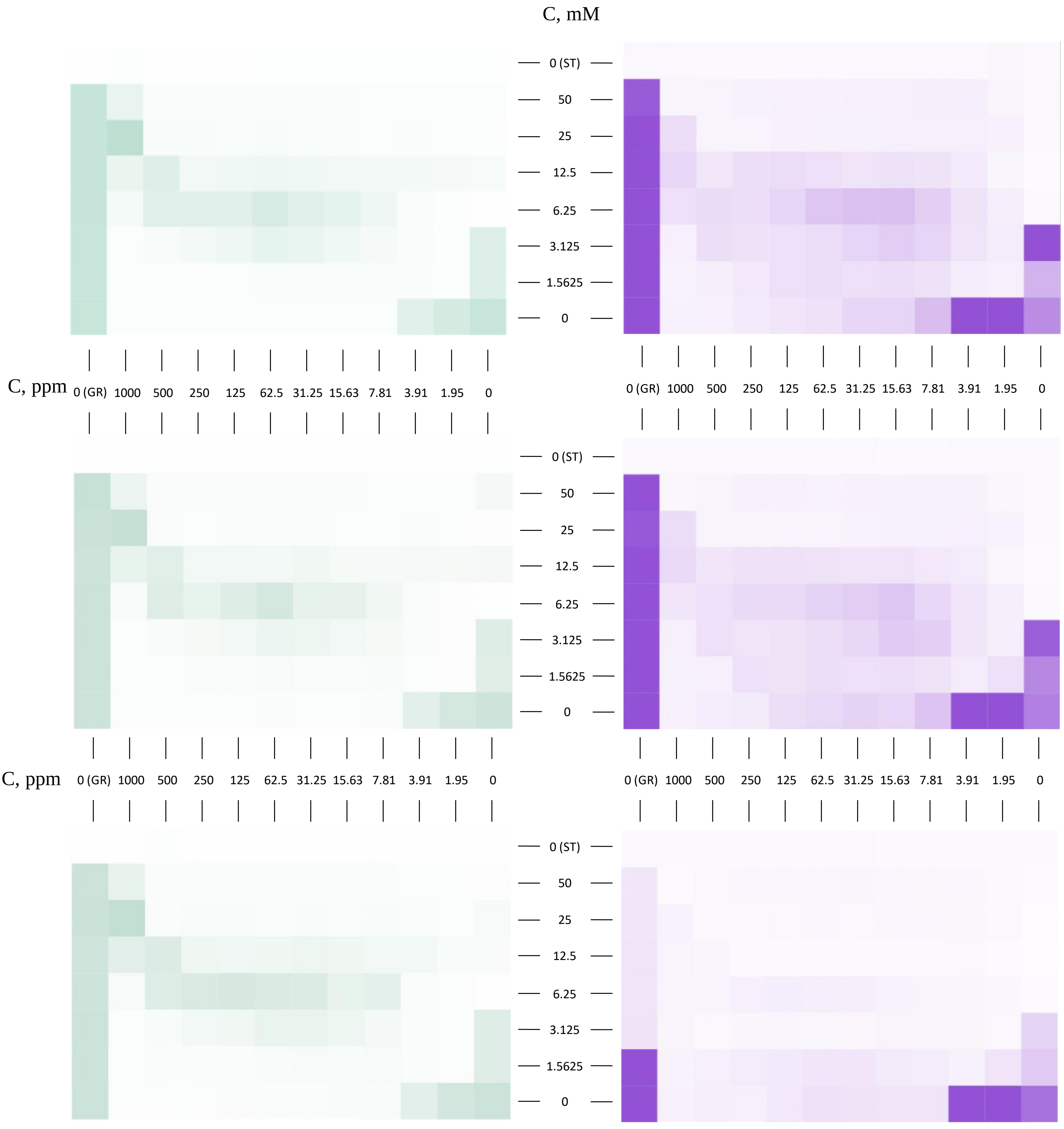

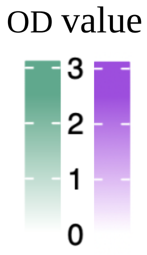

Figure S.1.7. CTAB/Al^{3+}, *P. aeruginosa*. Heatmaps of OD readings from the grown plates of planktonic (green to white) and biofilm (purple to white) growth of *P. aeruginosa* after 24h exposure to checkerboard assay of cetyltrimethylammonium bromide (CTAB, horizontal concentrations gradient) and aluminum chloride (Al^{3+}, vertical concentrations gradient).

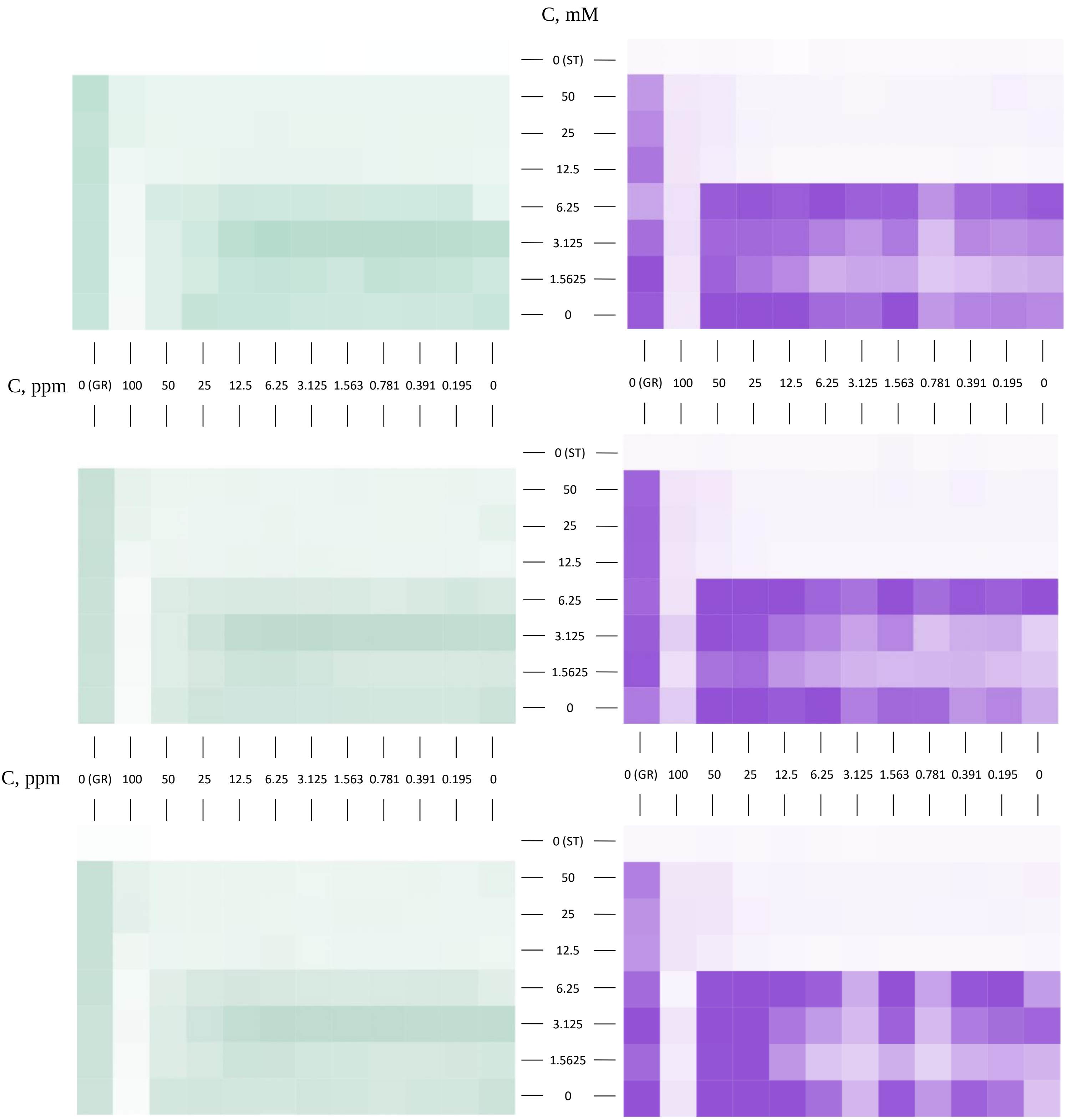

Figure S.1.8. CTAB/Cu^{2+}, *P. aeruginosa*. Heatmaps of OD readings from the grown plates of planktonic (green to white) and biofilm (purple to white) growth of *P. aeruginosa* after 24h exposure to checkerboard assay of cetyltrimethylammonium bromide (CTAB, horizontal concentrations gradient) and copper chloride (Cu^{2+}, vertical concentrations gradient).

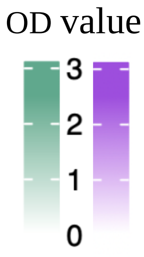

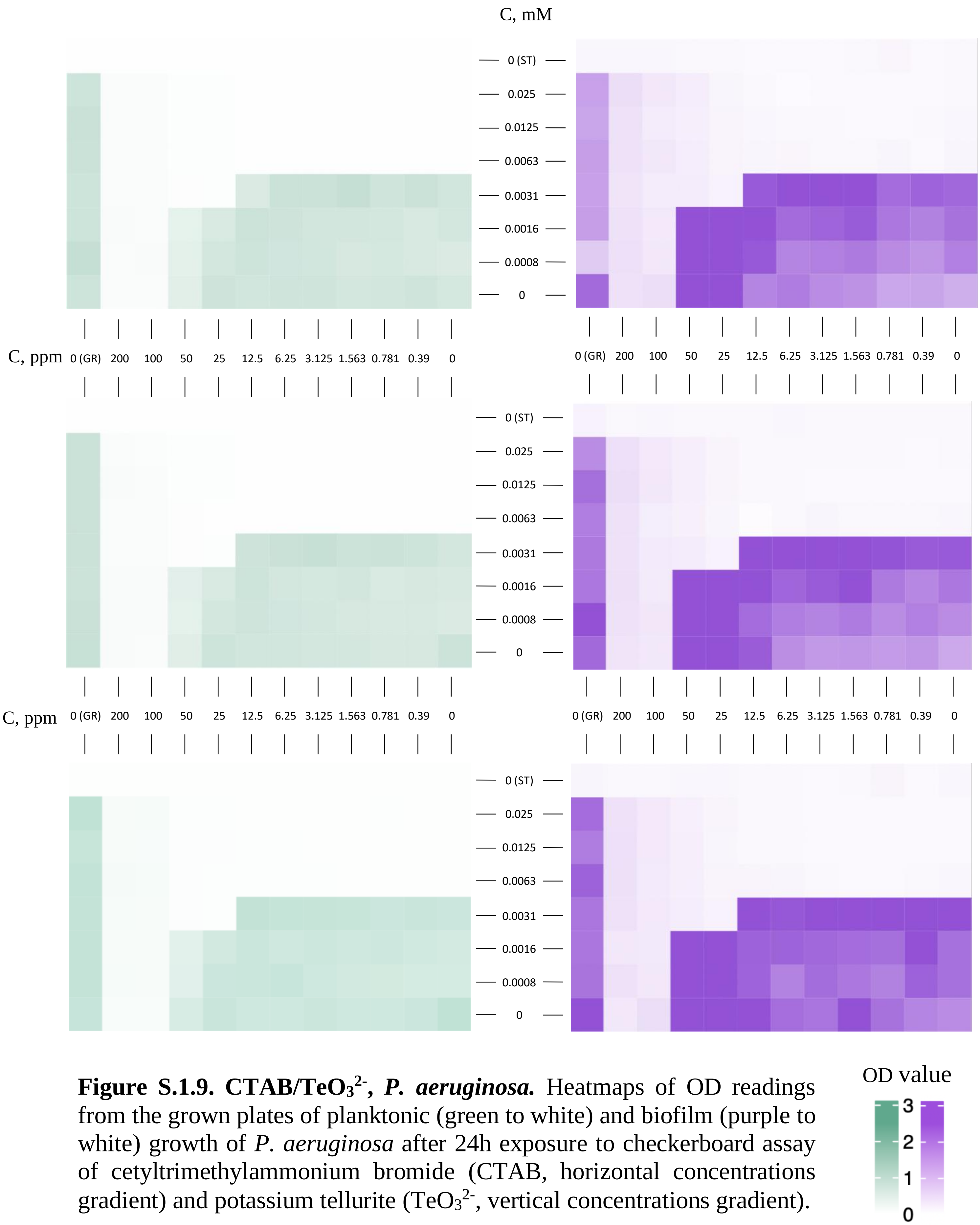

Figure S.1.9. CTAB/TeO_3^{2-}, *P. aeruginosa*. Heatmaps of OD readings from the grown plates of planktonic (green to white) and biofilm (purple to white) growth of *P. aeruginosa* after 24h exposure to checkerboard assay of cetyltrimethylammonium bromide (CTAB, horizontal concentrations gradient) and potassium tellurite (TeO_3^{2-}, vertical concentrations gradient).

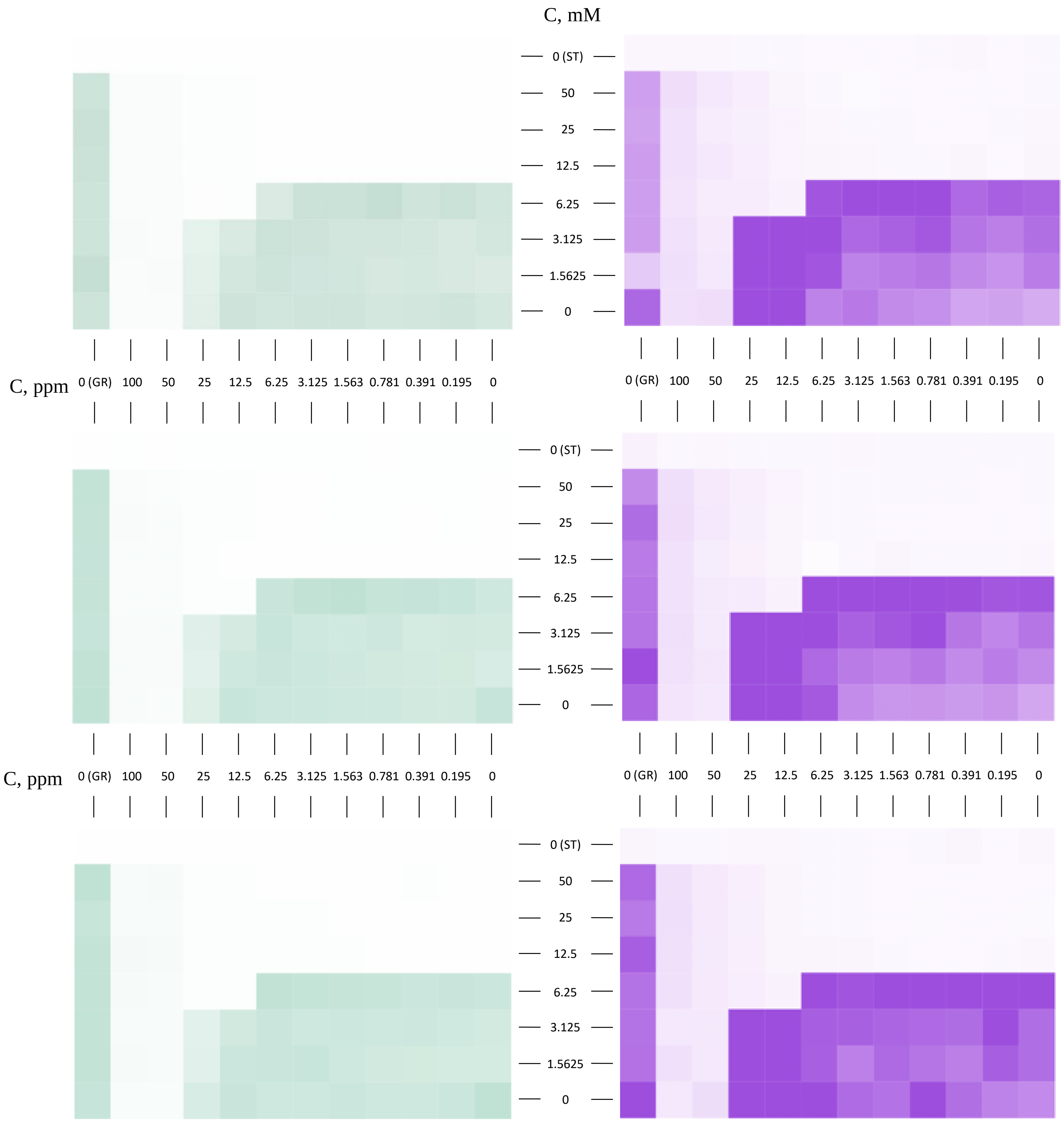

Figure S.1.10. CTAB/Zn^{2+}, *P. aeruginosa*. Heatmaps of OD readings from the grown plates of planktonic (green to white) and biofilm (purple to white) growth of *P. aeruginosa* after 24h exposure to checkerboard assay of cetyltrimethylammonium bromide (CTAB, horizontal concentrations gradient) and zinc chloride (Zn^{2+}, vertical concentrations gradient).

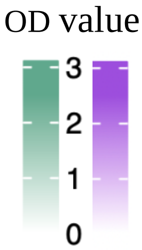

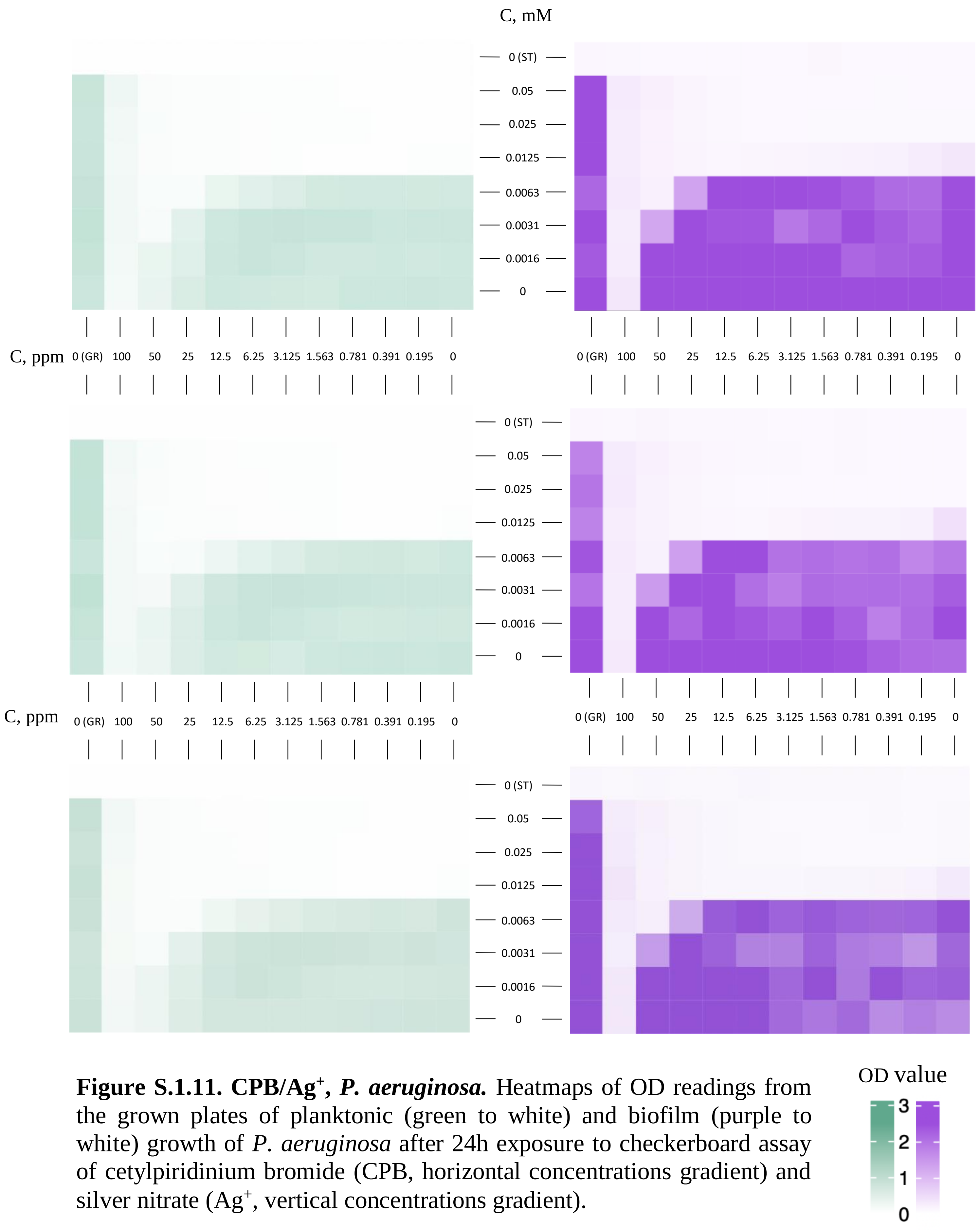

Figure S.1.11. CPB/Ag^{+}, *P. aeruginosa*. Heatmaps of OD readings from the grown plates of planktonic (green to white) and biofilm (purple to white) growth of *P. aeruginosa* after 24h exposure to checkerboard assay of cetylpiridinium bromide (CPB, horizontal concentrations gradient) and silver nitrate (Ag^{+}, vertical concentrations gradient).

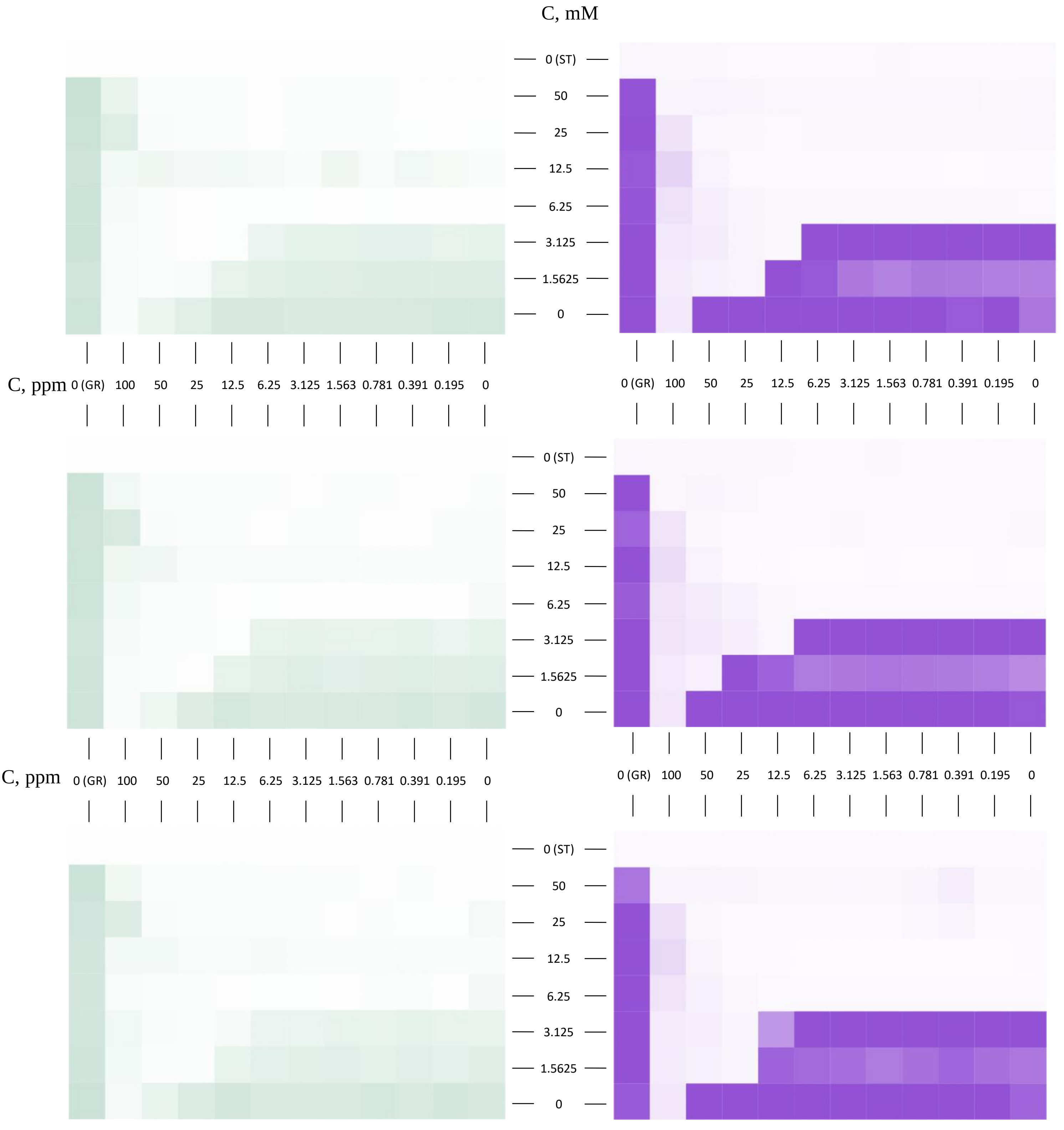

Figure S.1.12. CPB/Al^{3+}, *P. aeruginosa*. Heatmaps of OD readings from the grown plates of planktonic (green to white) and biofilm (purple to white) growth of *P. aeruginosa* after 24h exposure to checkerboard assay of cetylpiridinium bromide (CPB, horizontal concentrations gradient) and aluminum chloride (Al^{3+}, vertical concentrations gradient).

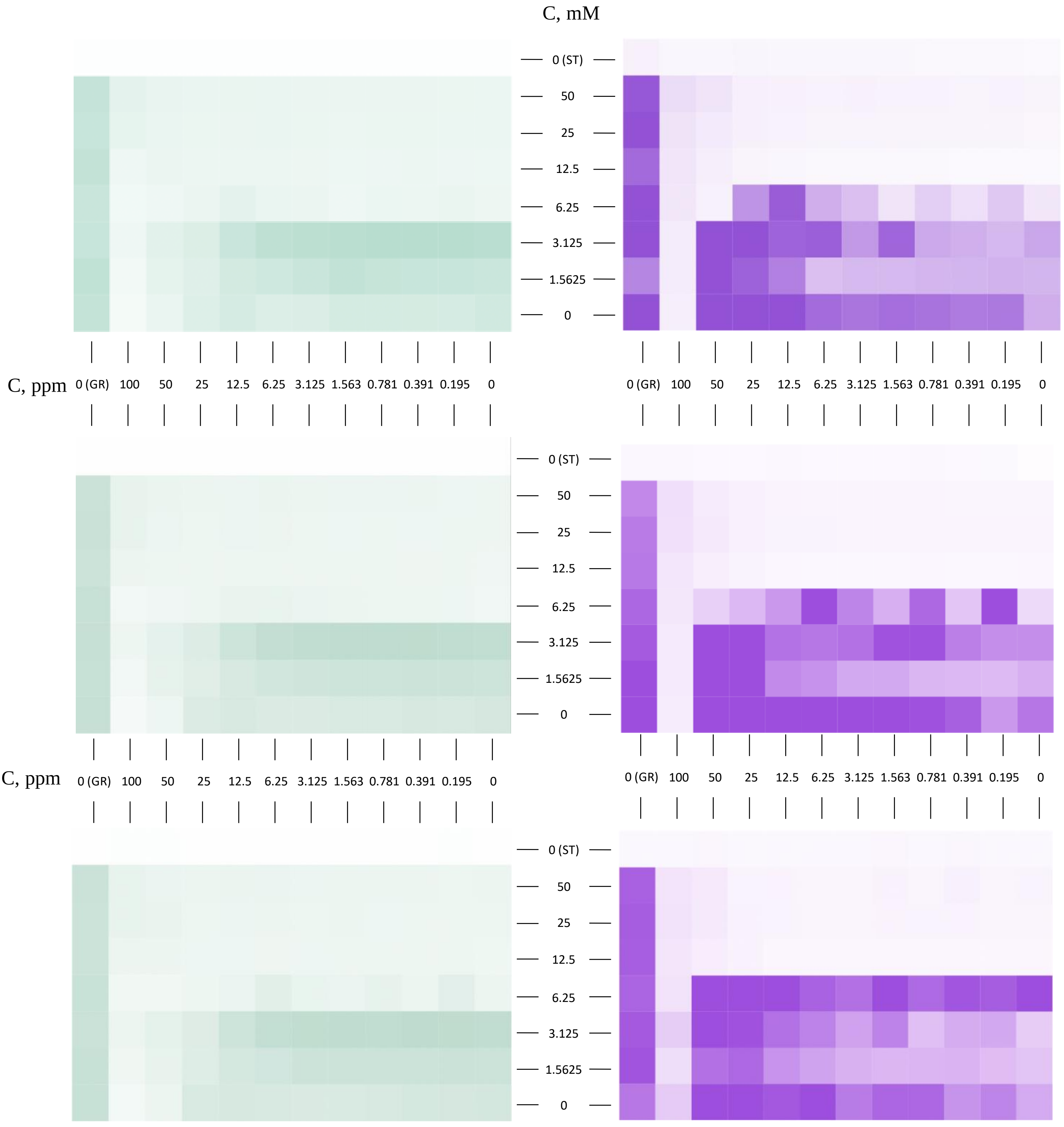

Figure S.1.13. CPB/Cu^{2+}, *P. aeruginosa*. Heatmaps of OD readings from the grown plates of planktonic (green to white) and biofilm (purple to white) growth of *P. aeruginosa* after 24h exposure to checkerboard assay of cetylpiridinium bromide (CPB, horizontal concentrations gradient) and copper chloride (Cu^{2+}, vertical concentrations gradient).

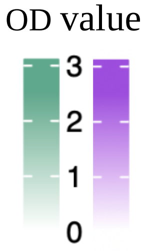

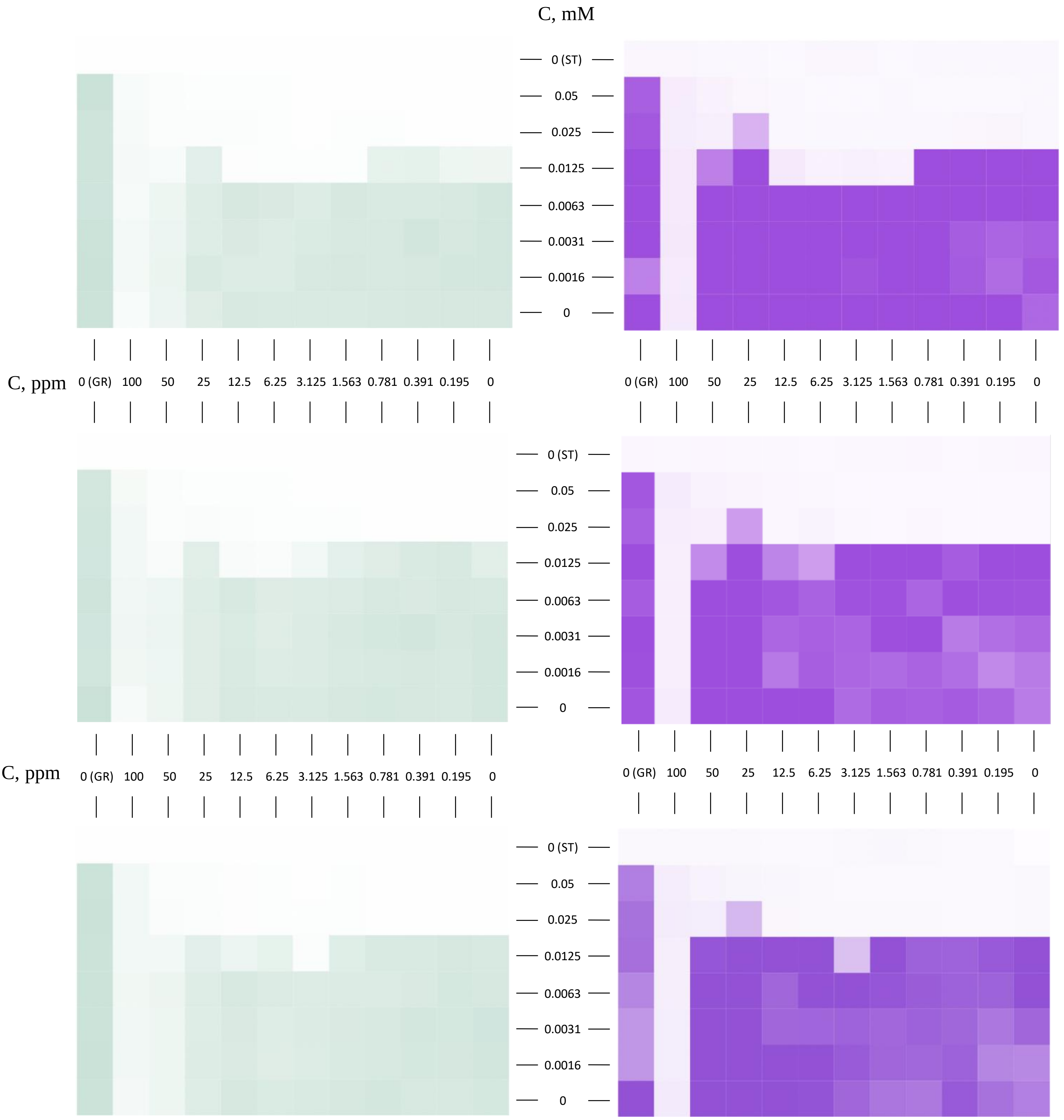

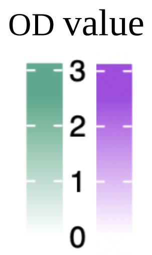

Figure S.1.14. CPB/TeO_3^{2-}, *P. aeruginosa*. Heatmaps of OD readings from the grown plates of planktonic (green to white) and biofilm (purple to white) growth of *P. aeruginosa* after 24h exposure to checkerboard assay of cetylpiridinium bromide (CPB, horizontal concentrations gradient) and potassium tellurite (TeO_3^{2-}, vertical concentrations gradient).

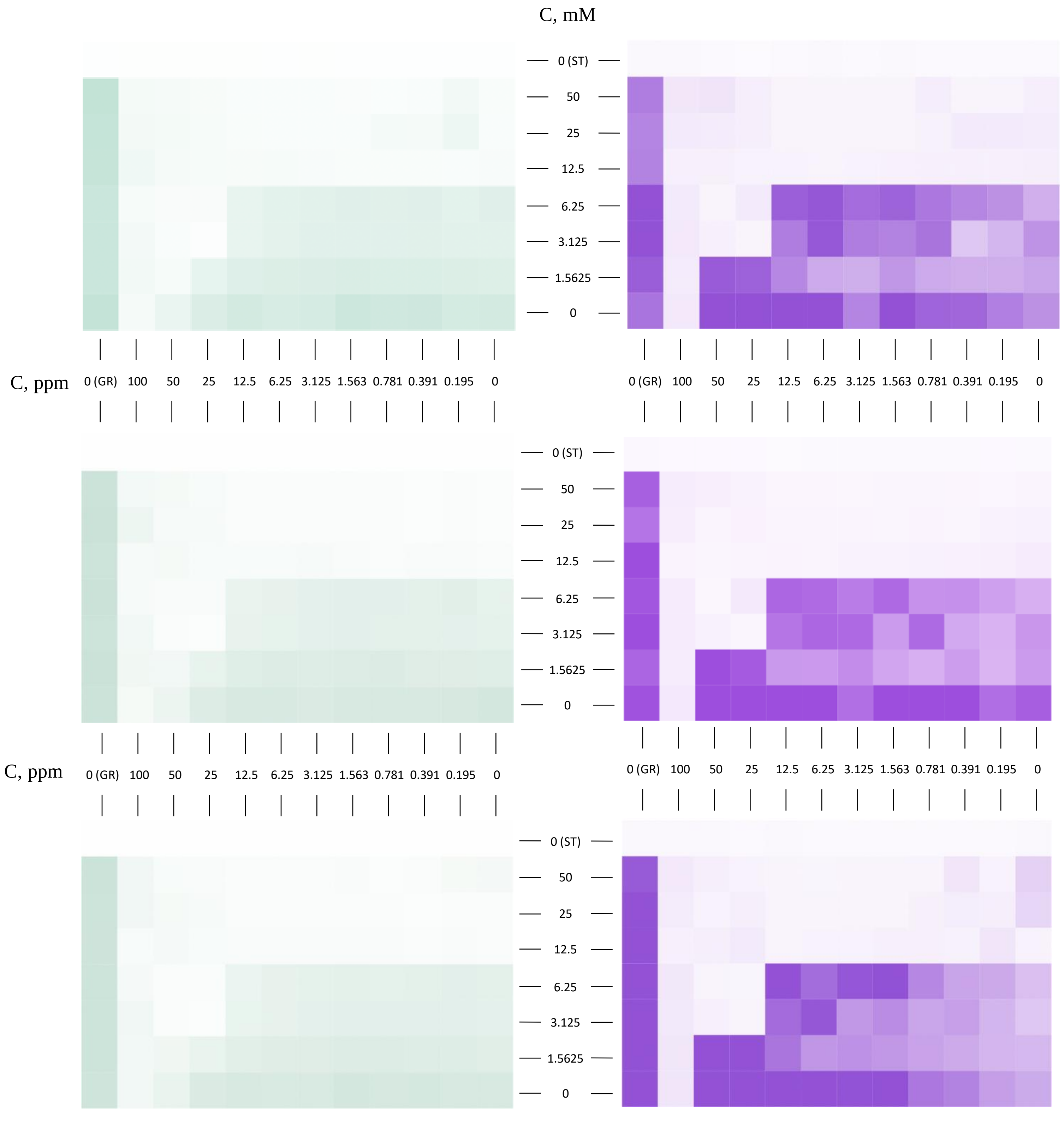

Figure S.1.15. CPB/Zn^{2+}, *P. aeruginosa*. Heatmaps of OD readings from the grown plates of planktonic (green to white) and biofilm (purple to white) growth of *P. aeruginosa* after 24h exposure to checkerboard assay of cetylpiridinium bromide (CPB, horizontal concentrations gradient) and zinc chloride (Zn^{2+}, vertical concentrations gradient).

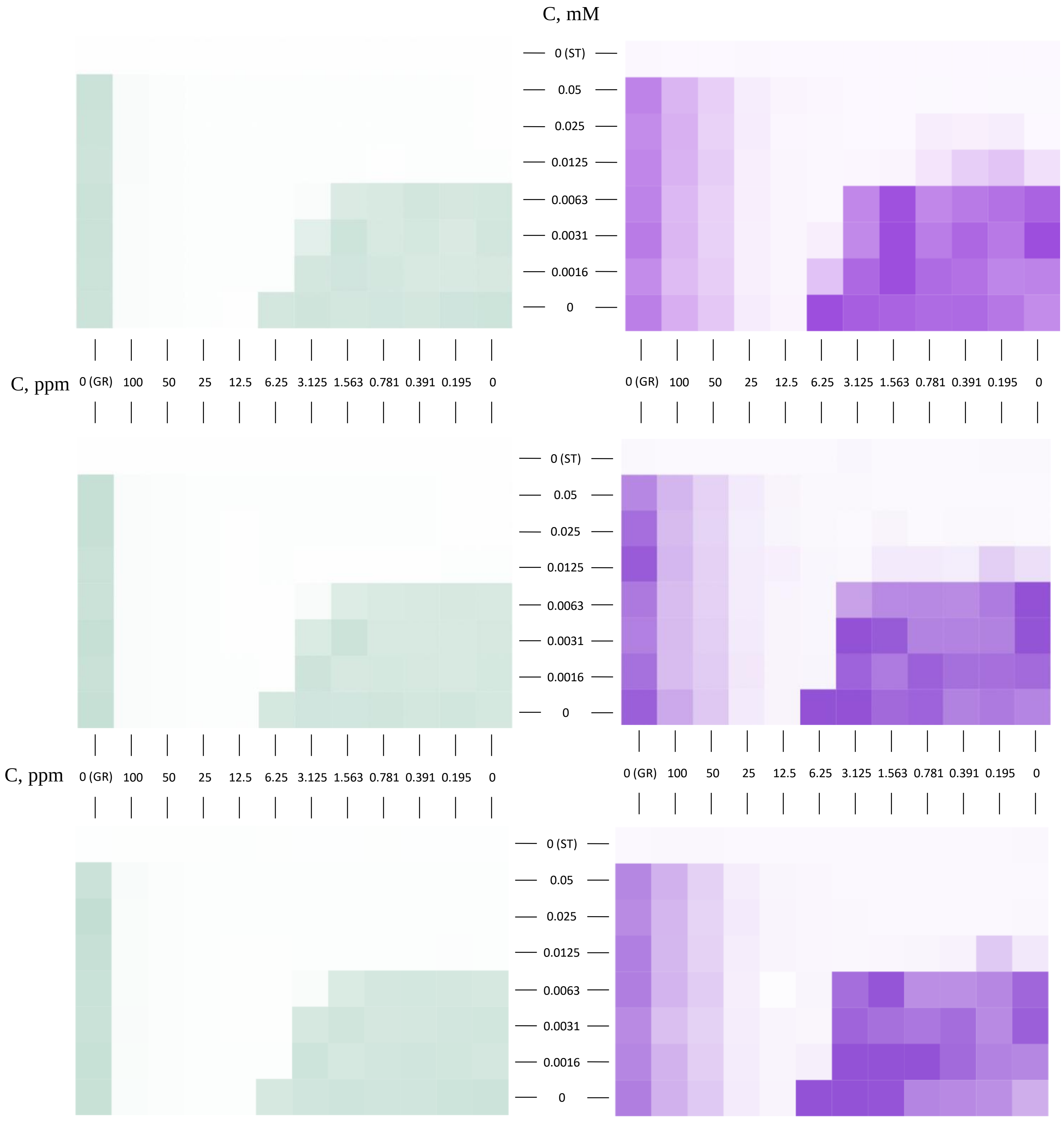

Figure S.1.16. DDAB/Ag^{+}, *P. aeruginosa.* Heatmaps of OD readings from the grown plates of planktonic (green to white) and biofilm (purple to white) growth of *P. aeruginosa* after 24h exposure to checkerboard assay of didecyldimethylammonium bromide (DDAB, horizontal concentrations gradient) and silver nitrate (Ag^{+}, vertical concentrations gradient).

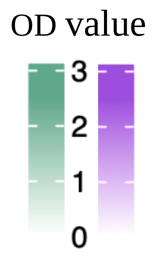

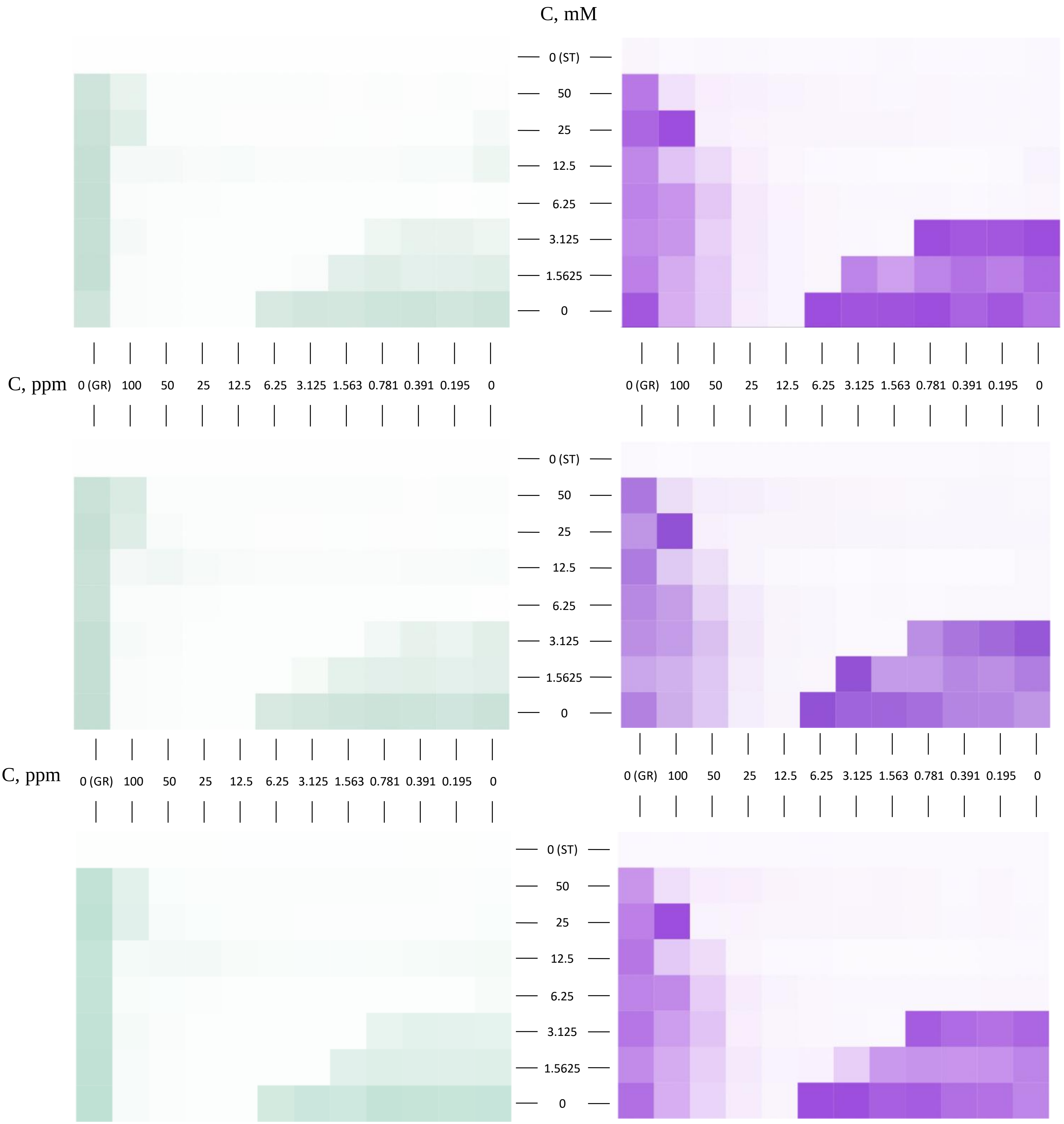

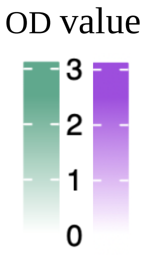

Figure S.1.17. DDAB/Al^{3+}, *P. aeruginosa*. Heatmaps of OD readings from the grown plates of planktonic (green to white) and biofilm (purple to white) growth of *P. aeruginosa* after 24h exposure to checkerboard assay of didecyldimethylammonium bromide (DDAB, horizontal concentrations gradient) and aluminum chloride (Cu^{2+}, vertical concentrations gradient).

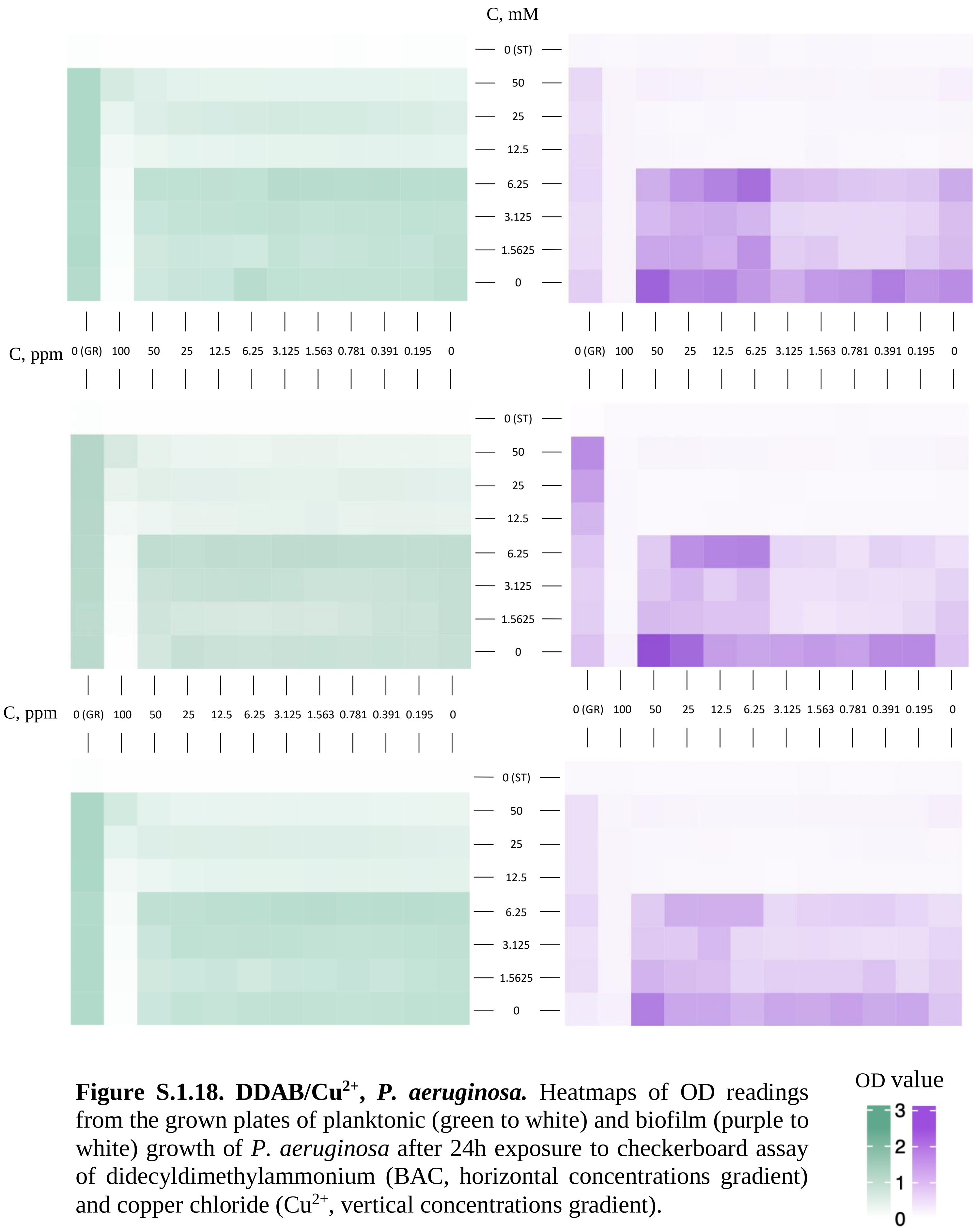

Figure S.1.18. DDAB/Cu^{2+}, *P. aeruginosa*. Heatmaps of OD readings from the grown plates of planktonic (green to white) and biofilm (purple to white) growth of *P. aeruginosa* after 24h exposure to checkerboard assay of didecyldimethylammonium (BAC, horizontal concentrations gradient) and copper chloride (Cu^{2+}, vertical concentrations gradient).

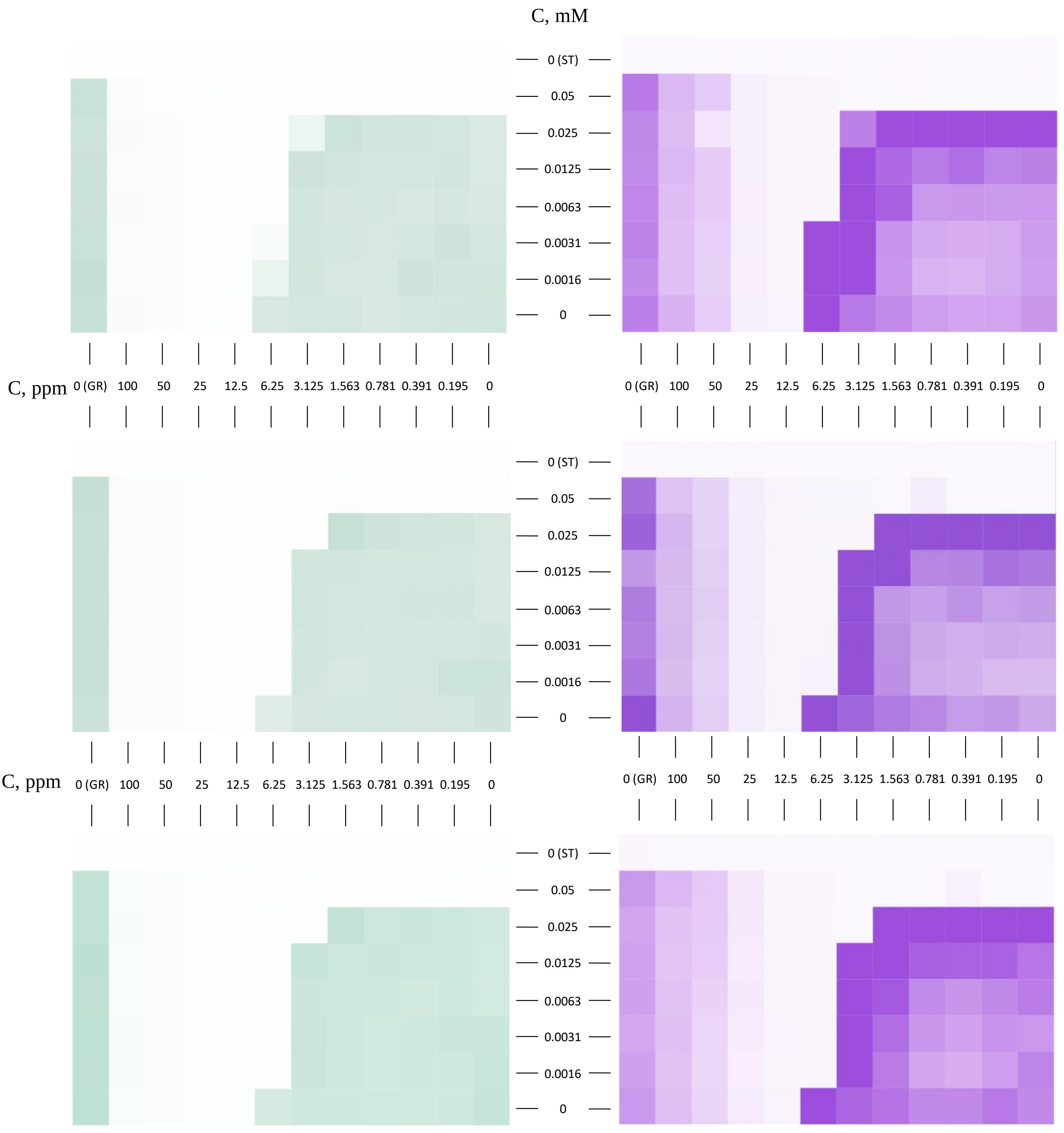

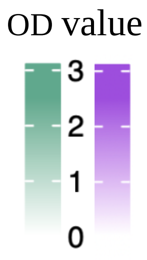

Figure S.1.19. DDAB/ TeO_3^{2-}, *P. aeruginosa.* Heatmaps of OD readings from the grown plates of planktonic (green to white) and biofilm (purple to white) growth of *P. aeruginosa* after 24h exposure to checkerboard assay of didecyldimethylammonium (BAC, horizontal concentrations gradient) and potassium tellurite (TeO_3^{2-}, vertical concentrations gradient).

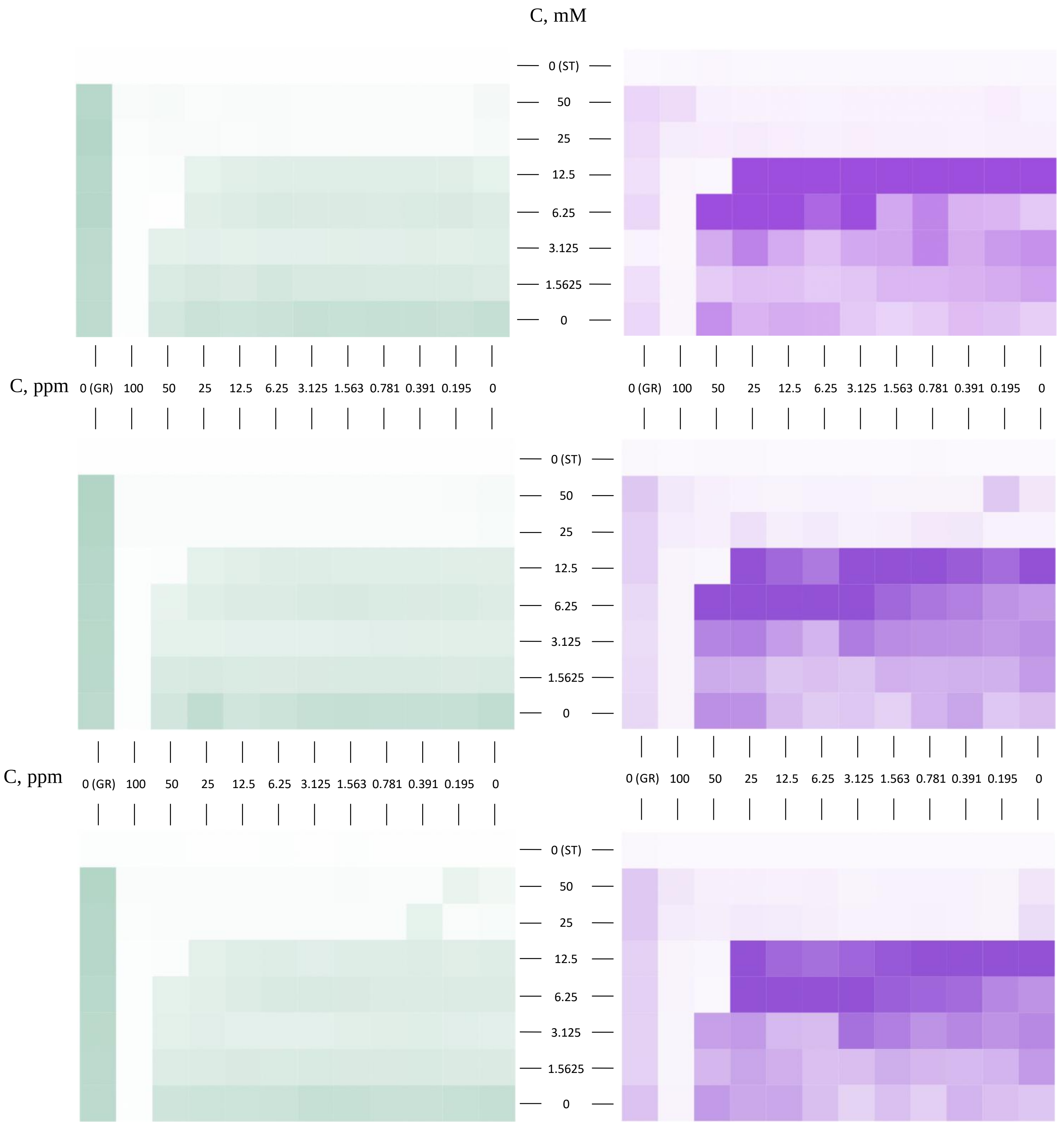

Figure S.1.20. DDAB/Zn^{2+}, *P. aeruginosa*. Heatmaps of OD readings from the grown plates of planktonic (green to white) and biofilm (purple to white) growth of *P. aeruginosa* after 24h exposure to checkerboard assay of didecyldimethylammonium bromide (DDAB, horizontal concentrations gradient) and zinc chloride (Zn^{2+}, vertical concentrations gradient).
