# Supplementary material for: Exploring antimicrobial interactions between metal ions and quaternary ammonium compounds toward synergistic metallo-antimicrobial formulations
Source: Microbiol Spectr. 2024 Aug 20;12(10):e01047-24. doi: 10.1128/spectrum.01047-24 (PMC11448152; doi:10.1128/spectrum.01047-24)
Supplement: Fig. S2 — E. coli checkerboard data. [file spectrum.01047-24-s0002.pdf]

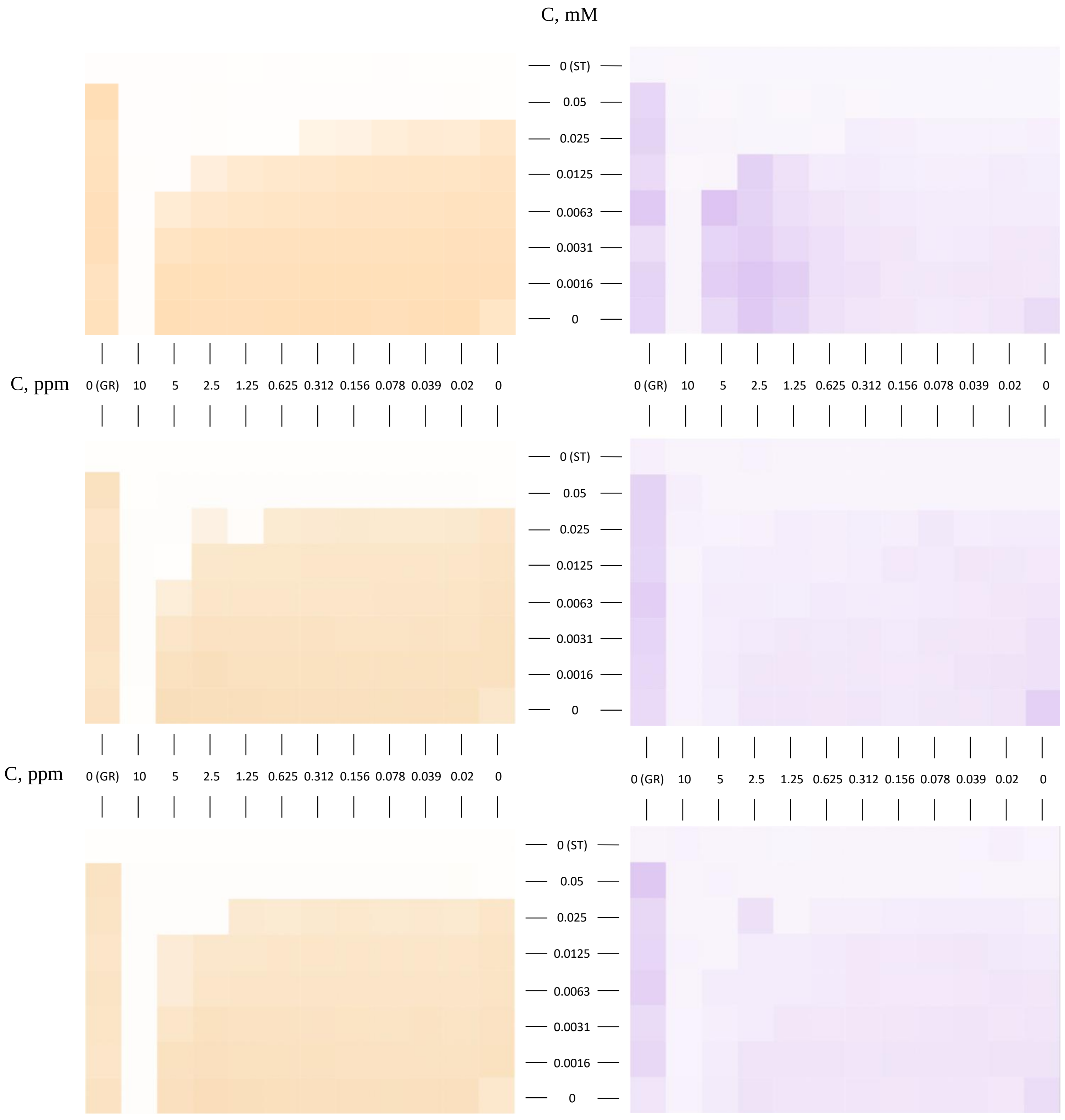

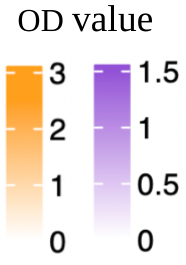

Figure S.2.1. BAC/Ag^{+}, *E. coli*. Heatmaps of OD readings from the grown plates of planktonic (orange to white) and biofilm (purple to white) growth of *E. coli* after 24h exposure to checkerboard assay of benzalkonium chloride (BAC, horizontal concentrations gradient) and silver nitrate (Ag^{+}, vertical concentrations gradient).

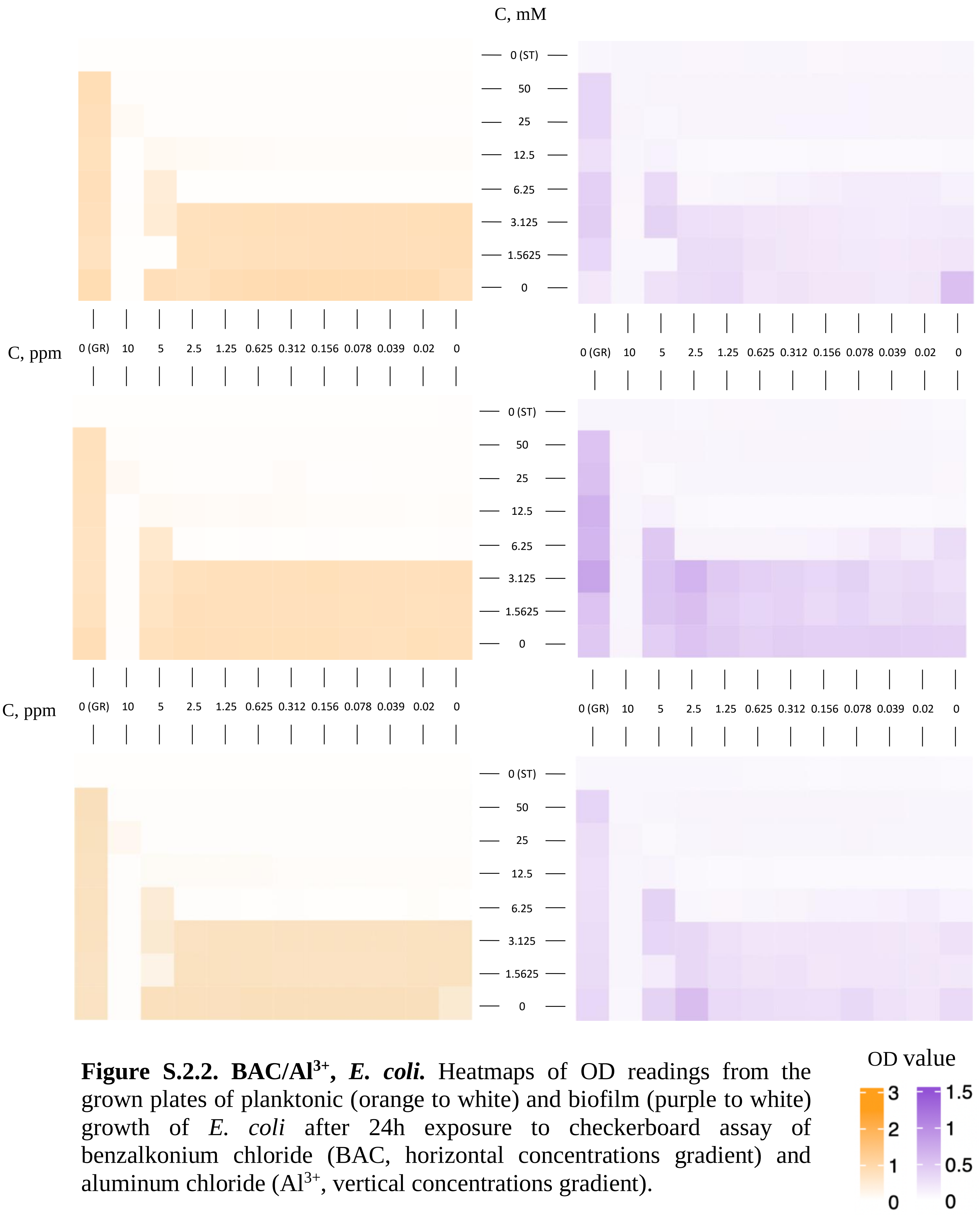

Figure S.2.2. BAC/Al^{3+}, *E. coli*. Heatmaps of OD readings from the grown plates of planktonic (orange to white) and biofilm (purple to white) growth of *E. coli* after 24h exposure to checkerboard assay of benzalkonium chloride (BAC, horizontal concentrations gradient) and aluminum chloride (Al^{3+}, vertical concentrations gradient).

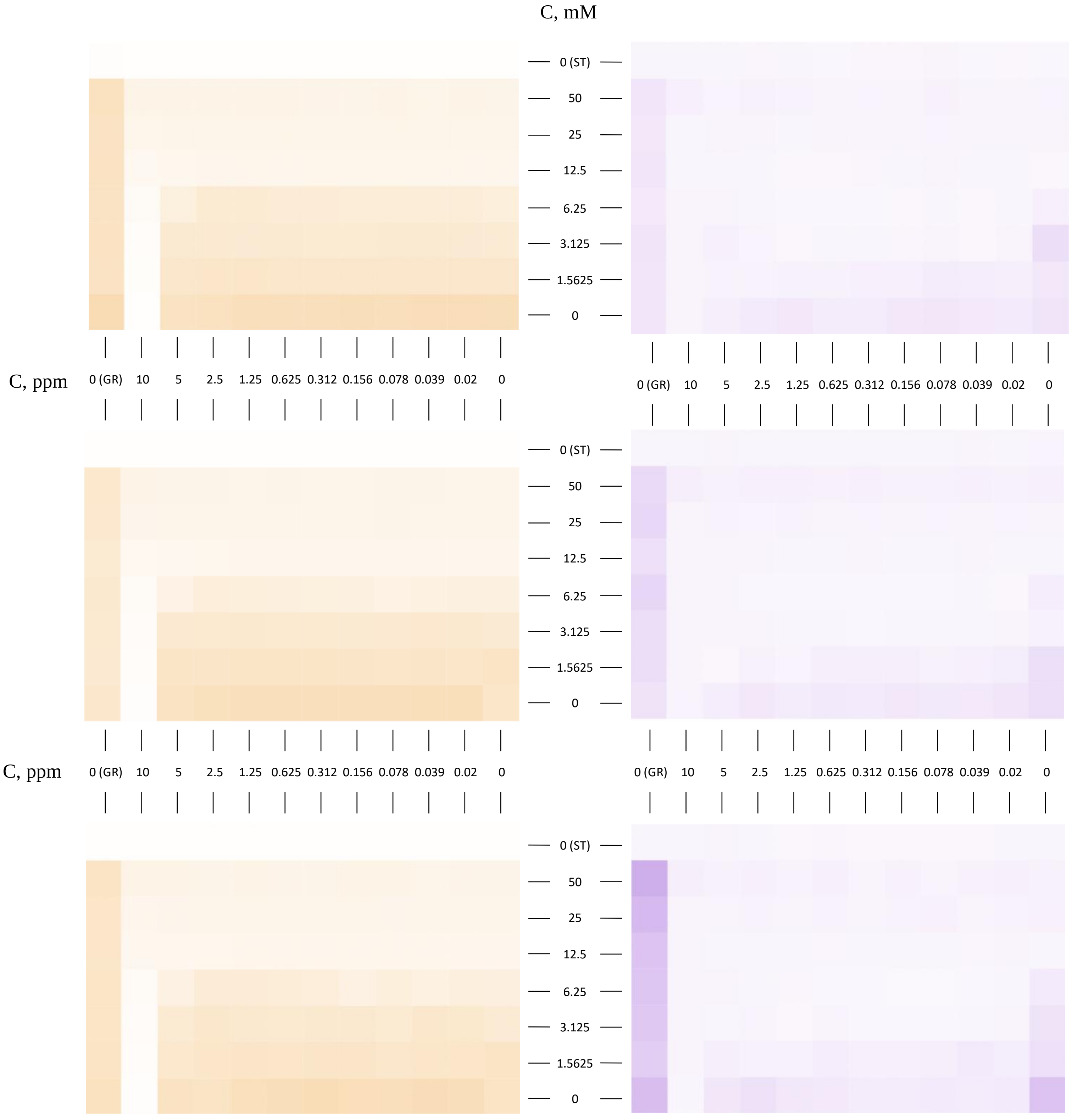

Figure S.2.3. BAC/Cu^{2+}, *E. coli.* Heatmaps of OD readings from the grown plates of planktonic (orange to white) and biofilm (purple to white) growth of *E. coli* after 24h exposure to checkerboard assay of benzalkonium chloride (BAC, horizontal concentrations gradient) and copper chloride (Cu^{2+}, vertical concentrations gradient).

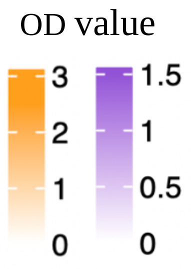

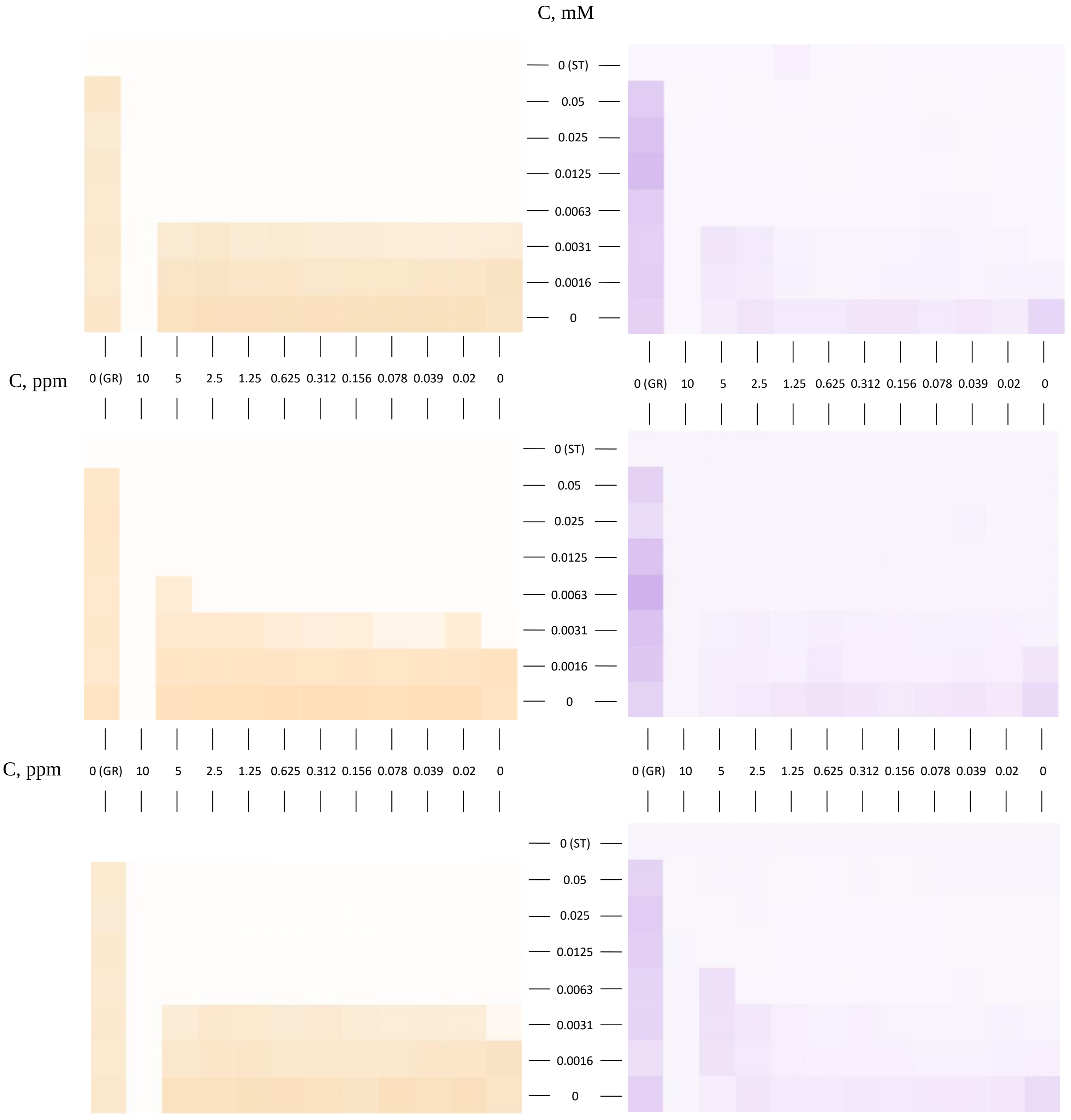

Figure S.2.4. BAC/TeO$_3^{2-}$, *E. coli*. Heatmaps of OD readings from the grown plates of planktonic (orange to white) and biofilm (purple to white) growth of *E. coli* after 24h exposure to checkerboard assay of benzalkonium chloride (BAC, horizontal concentrations gradient) and potassium tellurite (TeO$_3^{2-}$, vertical concentrations gradient).

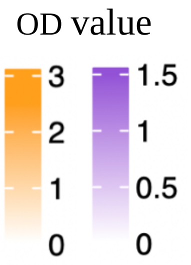

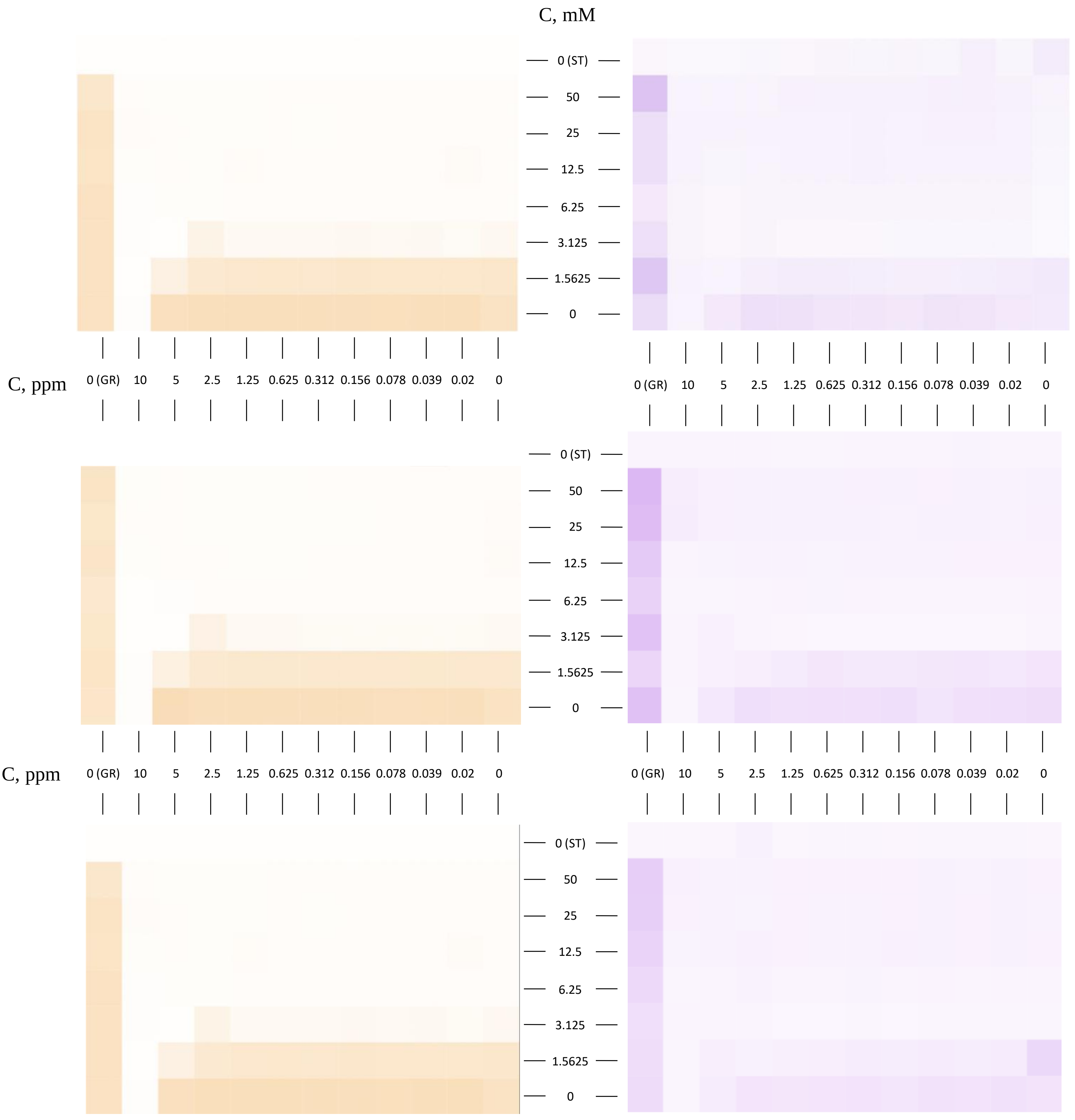

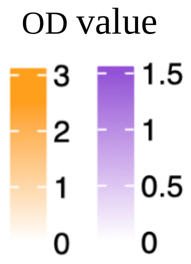

Figure S.2.5. BAC/Zn^{2+}, *E. coli*. Heatmaps of OD readings from the grown plates of planktonic (orange to white) and biofilm (purple to white) growth of *E. coli* after 24h exposure to checkerboard assay of benzalkonium chloride (BAC, horizontal concentrations gradient) and zinc chloride (Zn^{2+}, vertical concentrations gradient).

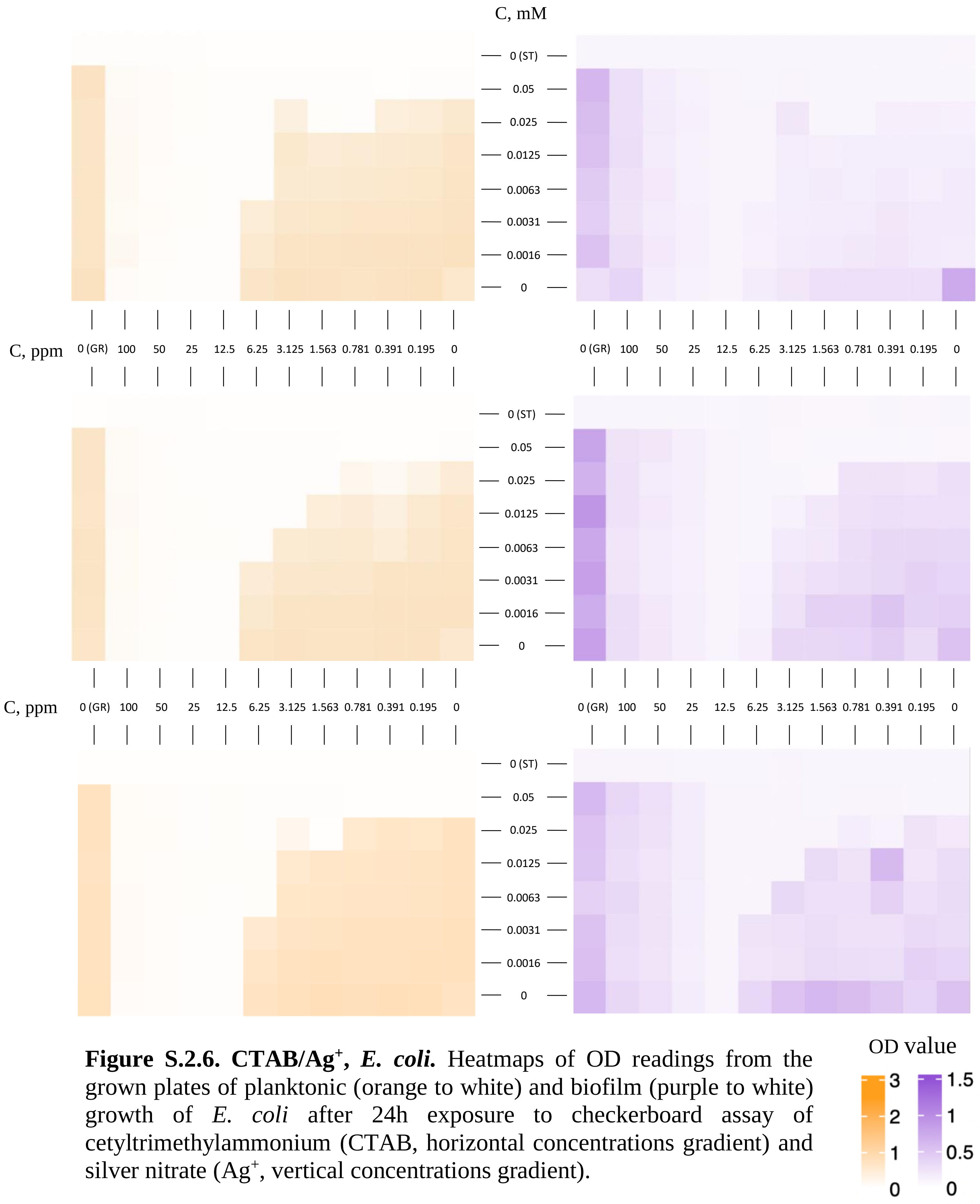

Figure S.2.6. CTAB/Ag⁺, *E. coli*. Heatmaps of OD readings from the grown plates of planktonic (orange to white) and biofilm (purple to white) growth of *E. coli* after 24h exposure to checkerboard assay of cetyltrimethylammonium (CTAB, horizontal concentrations gradient) and silver nitrate (Ag^+, vertical concentrations gradient).

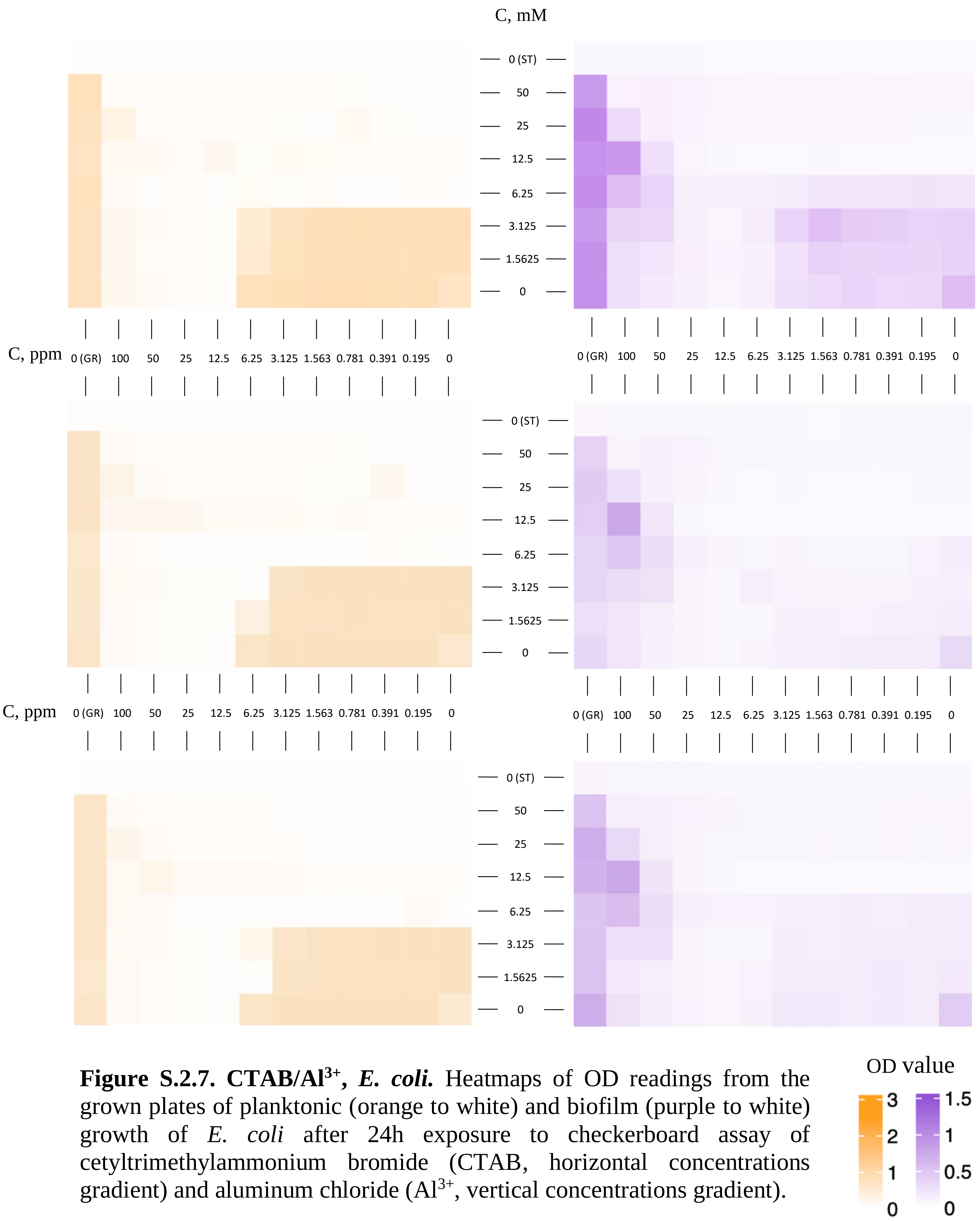

Figure S.2.7. CTAB/Al^{3+}, *E. coli*. Heatmaps of OD readings from the grown plates of planktonic (orange to white) and biofilm (purple to white) growth of *E. coli* after 24h exposure to checkerboard assay of cetyltrimethylammonium bromide (CTAB, horizontal concentrations gradient) and aluminum chloride (Al^{3+}, vertical concentrations gradient).

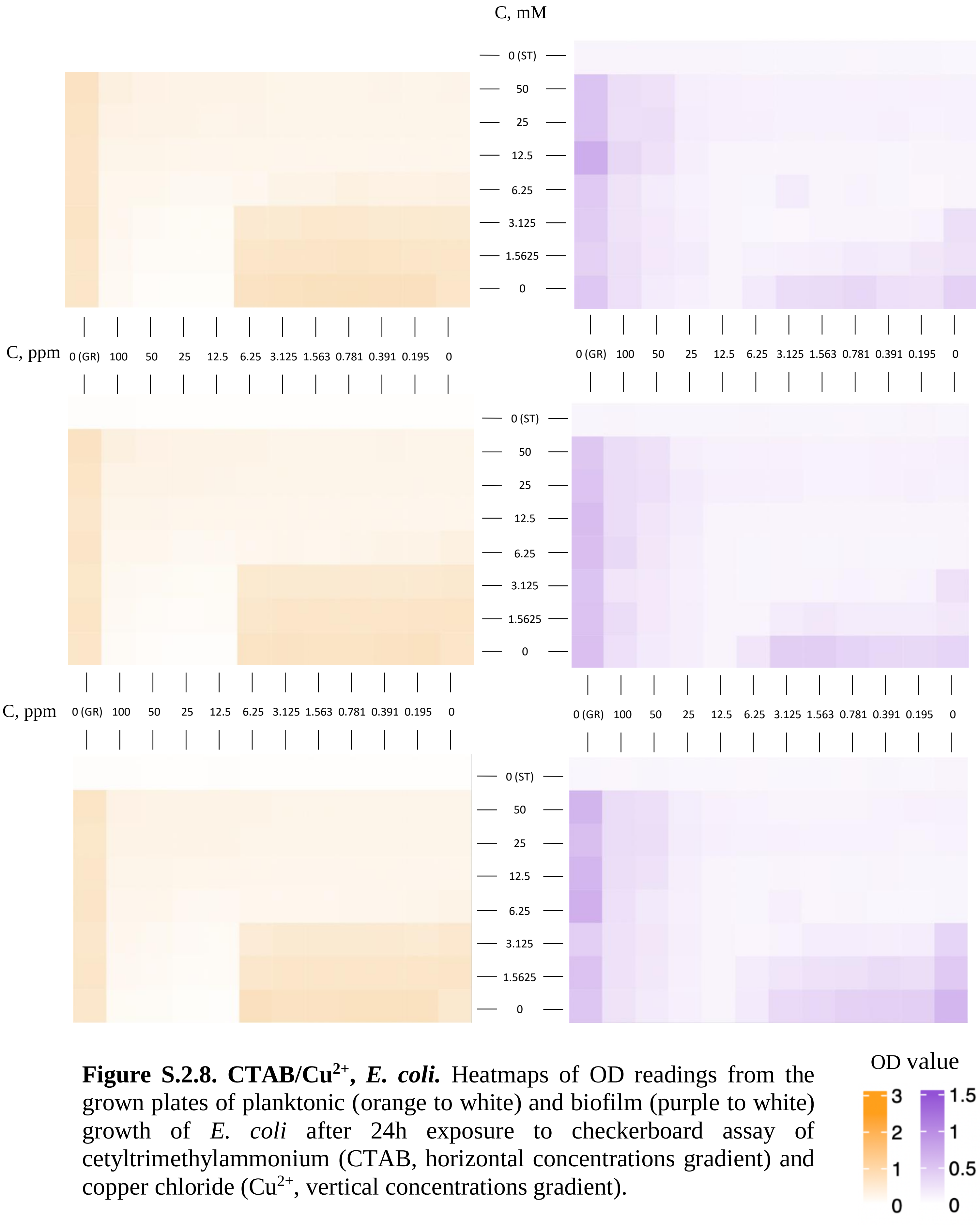

Figure S.2.8. CTAB/Cu^{2+}, *E. coli*. Heatmaps of OD readings from the grown plates of planktonic (orange to white) and biofilm (purple to white) growth of *E. coli* after 24h exposure to checkerboard assay of cetyltrimethylammonium (CTAB, horizontal concentrations gradient) and copper chloride (Cu^{2+}, vertical concentrations gradient).

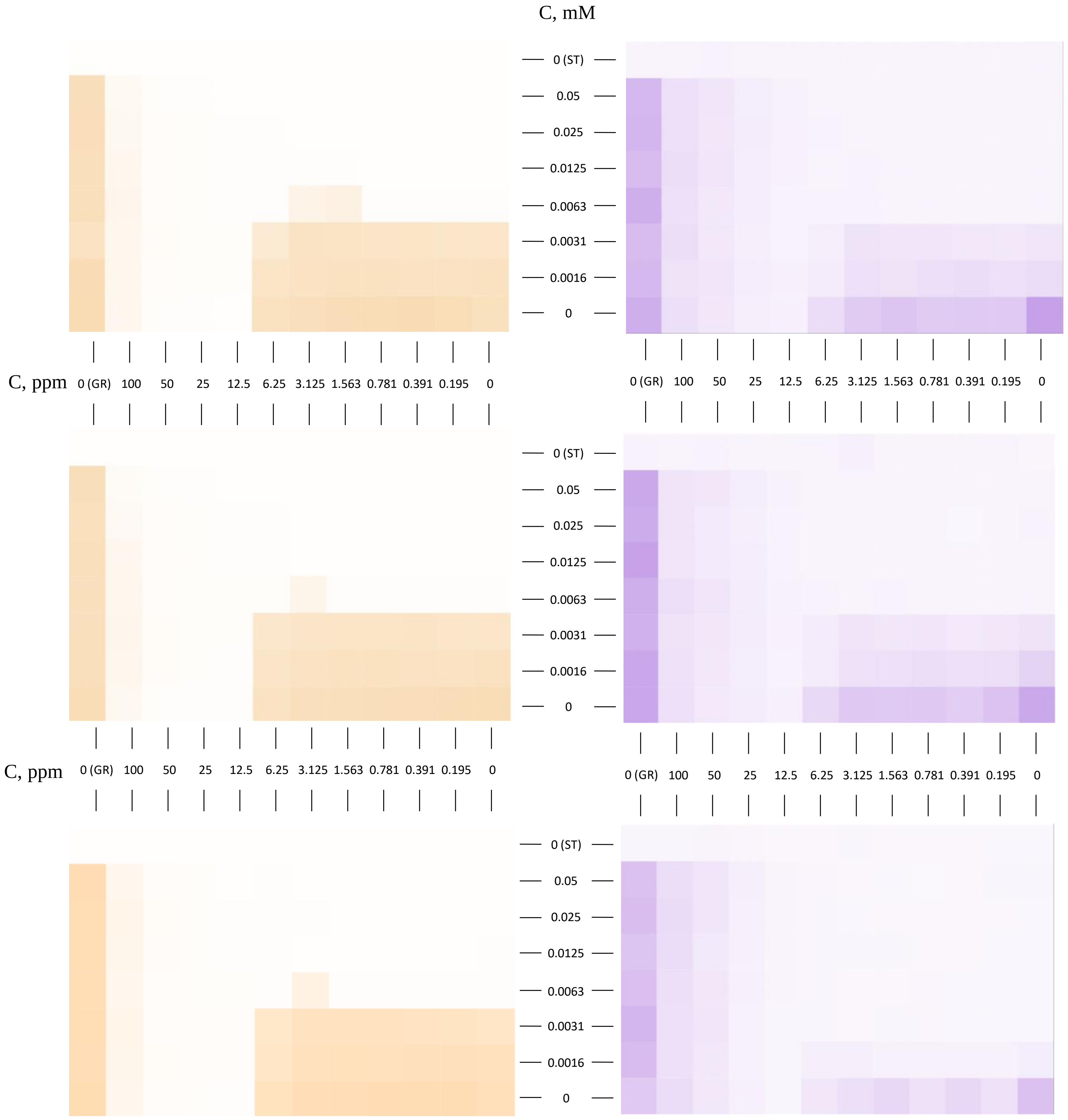

Figure S.2.9. CTAB/TeO_3^{2-}, *E. coli*. Heatmaps of OD readings from the grown plates of planktonic (orange to white) and biofilm (purple to white) growth of *E. coli* after 24h exposure to checkerboard assay of cetyltrimethylammonium bromide (CTAB, horizontal concentrations gradient) and potassium tellurite (TeO_3^{2-}, vertical concentrations gradient).

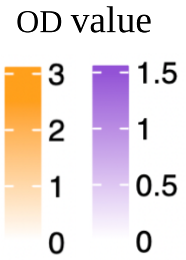

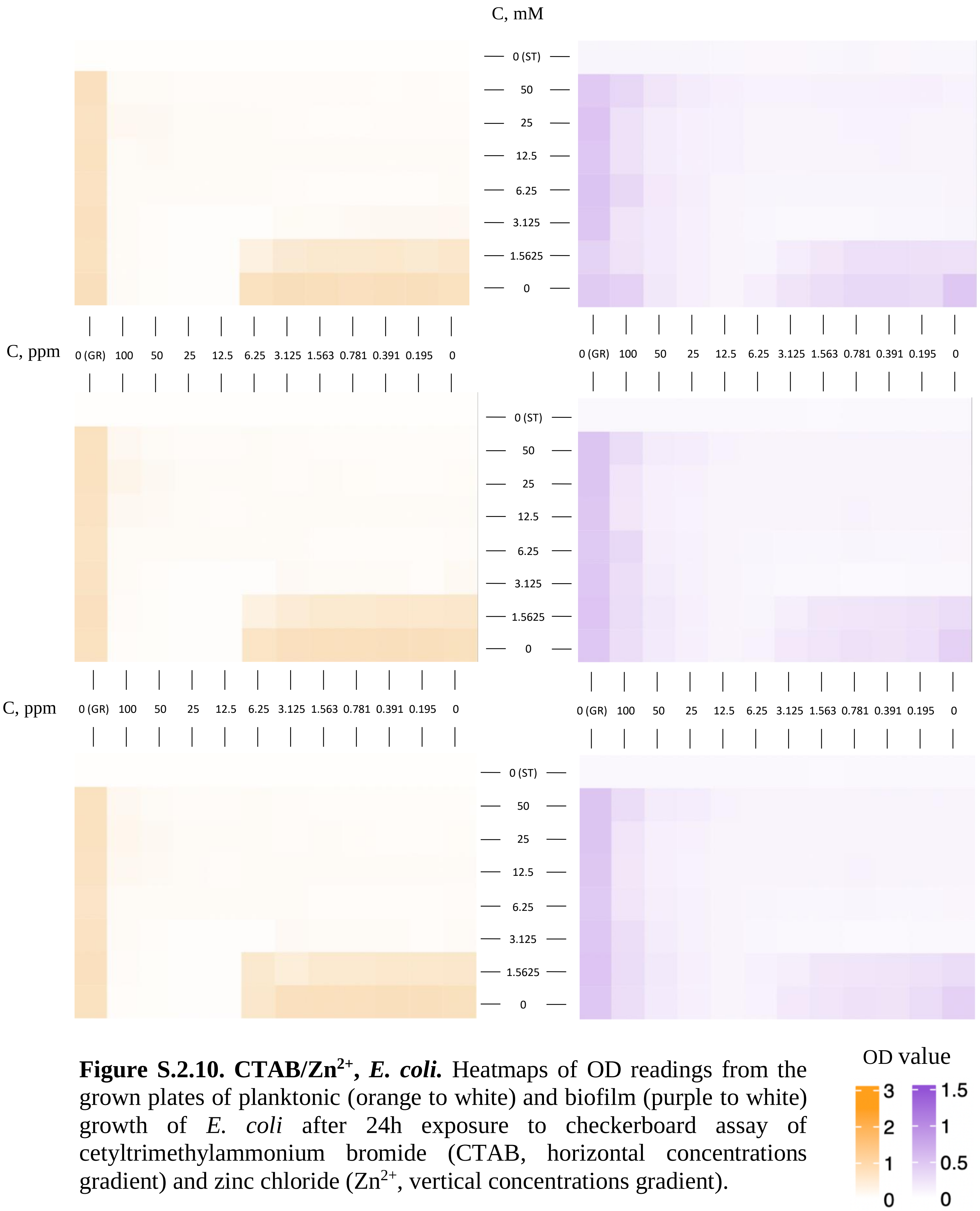

Figure S.2.10. CTAB/Zn^{2+}, *E. coli*. Heatmaps of OD readings from the grown plates of planktonic (orange to white) and biofilm (purple to white) growth of *E. coli* after 24h exposure to checkerboard assay of cetyltrimethylammonium bromide (CTAB, horizontal concentrations gradient) and zinc chloride (Zn^{2+}, vertical concentrations gradient).

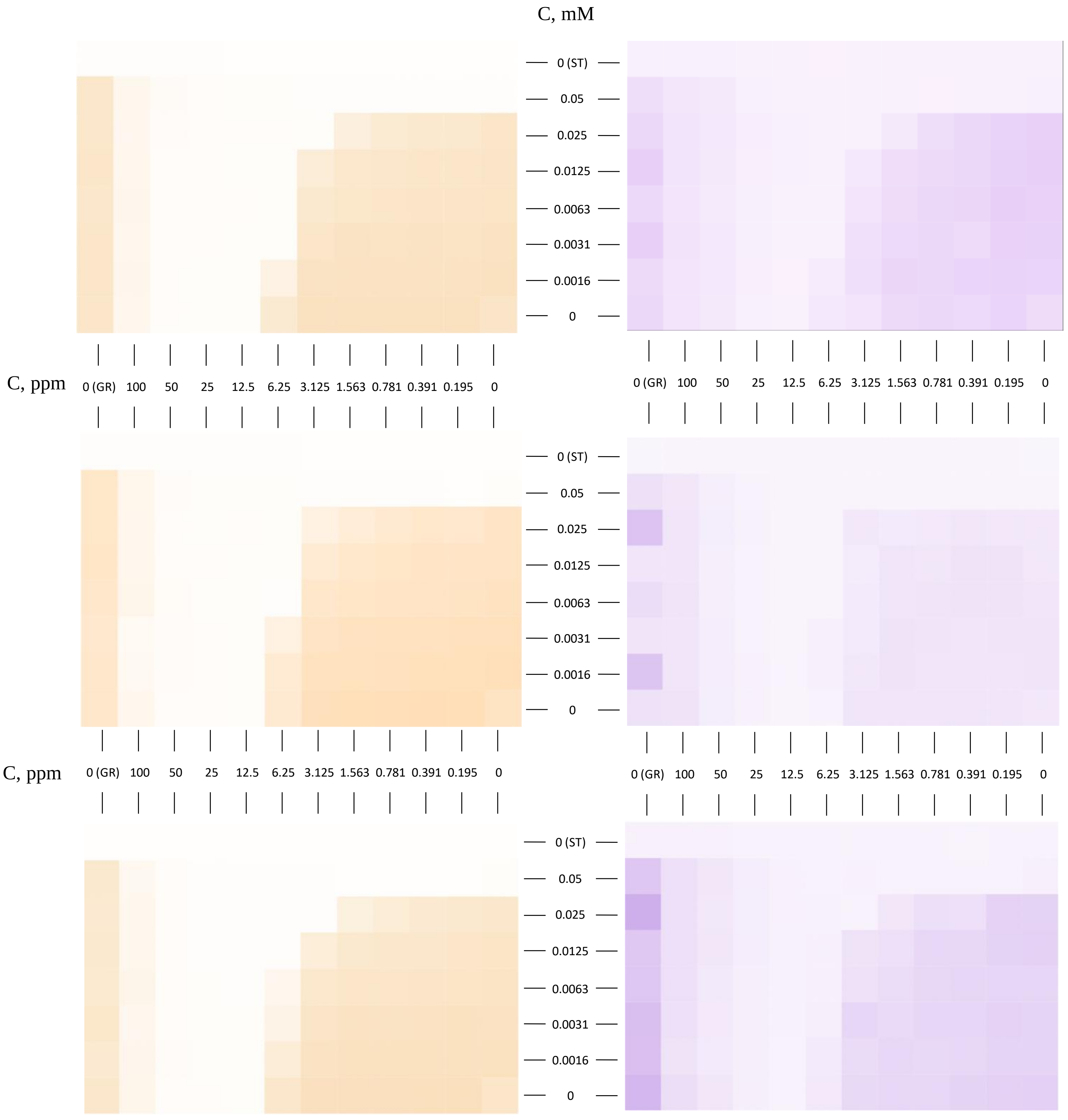

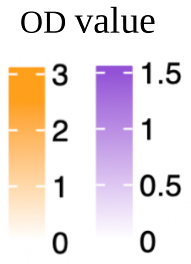

Figure S.2.11. CPB/Ag^{+}, *E. coli.* Heatmaps of OD readings from the grown plates of planktonic (orange to white) and biofilm (purple to white) growth of *E. coli* after 24h exposure to checkerboard assay of cetylpiridinium bromide (CPB, horizontal concentrations gradient) and silver nitrate (Ag^{+}, vertical concentrations gradient).

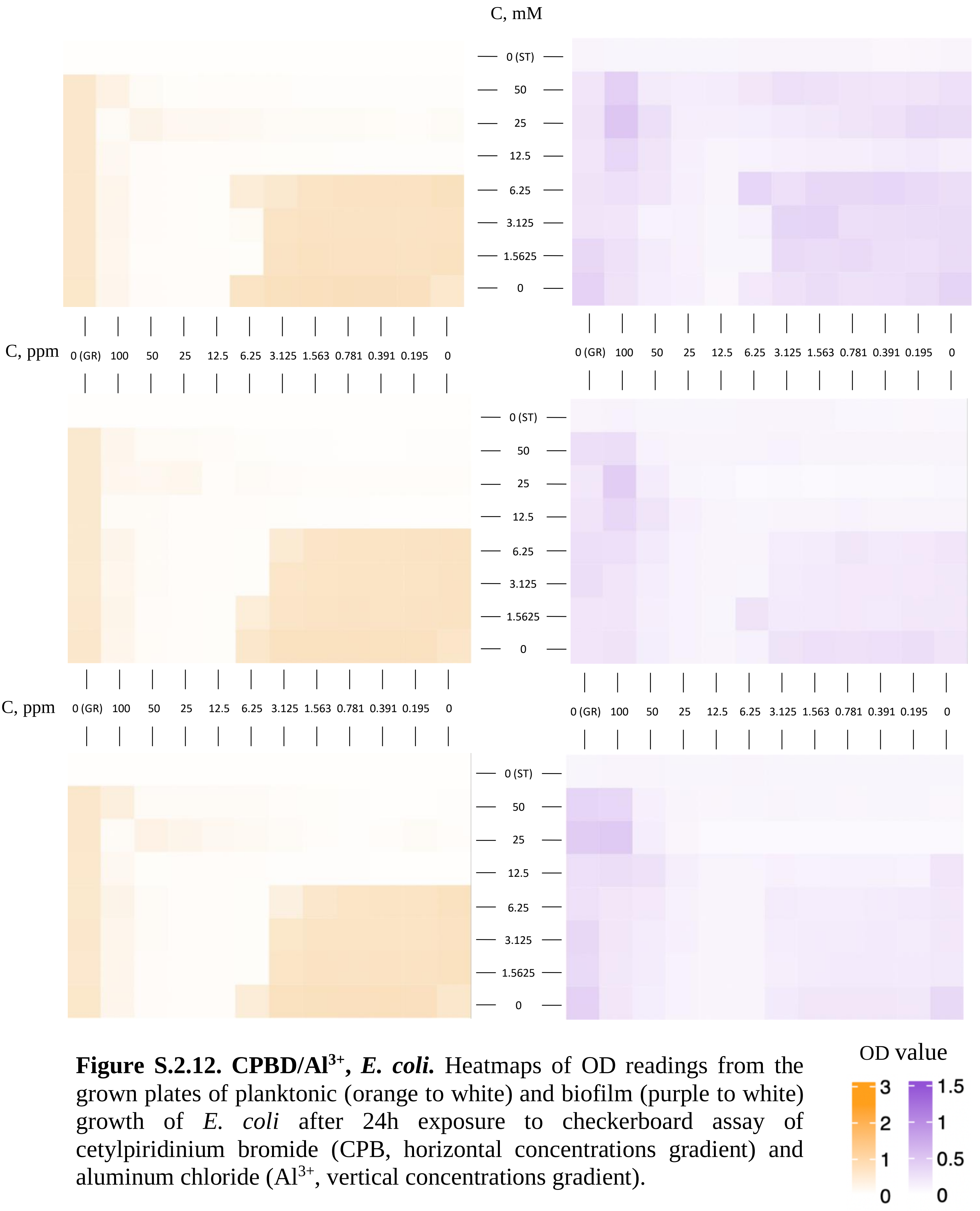

Figure S.2.12. CPBD/Al^{3+}, *E. coli*. Heatmaps of OD readings from the grown plates of planktonic (orange to white) and biofilm (purple to white) growth of *E. coli* after 24h exposure to checkerboard assay of cetylpiridinium bromide (CPB, horizontal concentrations gradient) and aluminum chloride (Al^{3+}, vertical concentrations gradient).

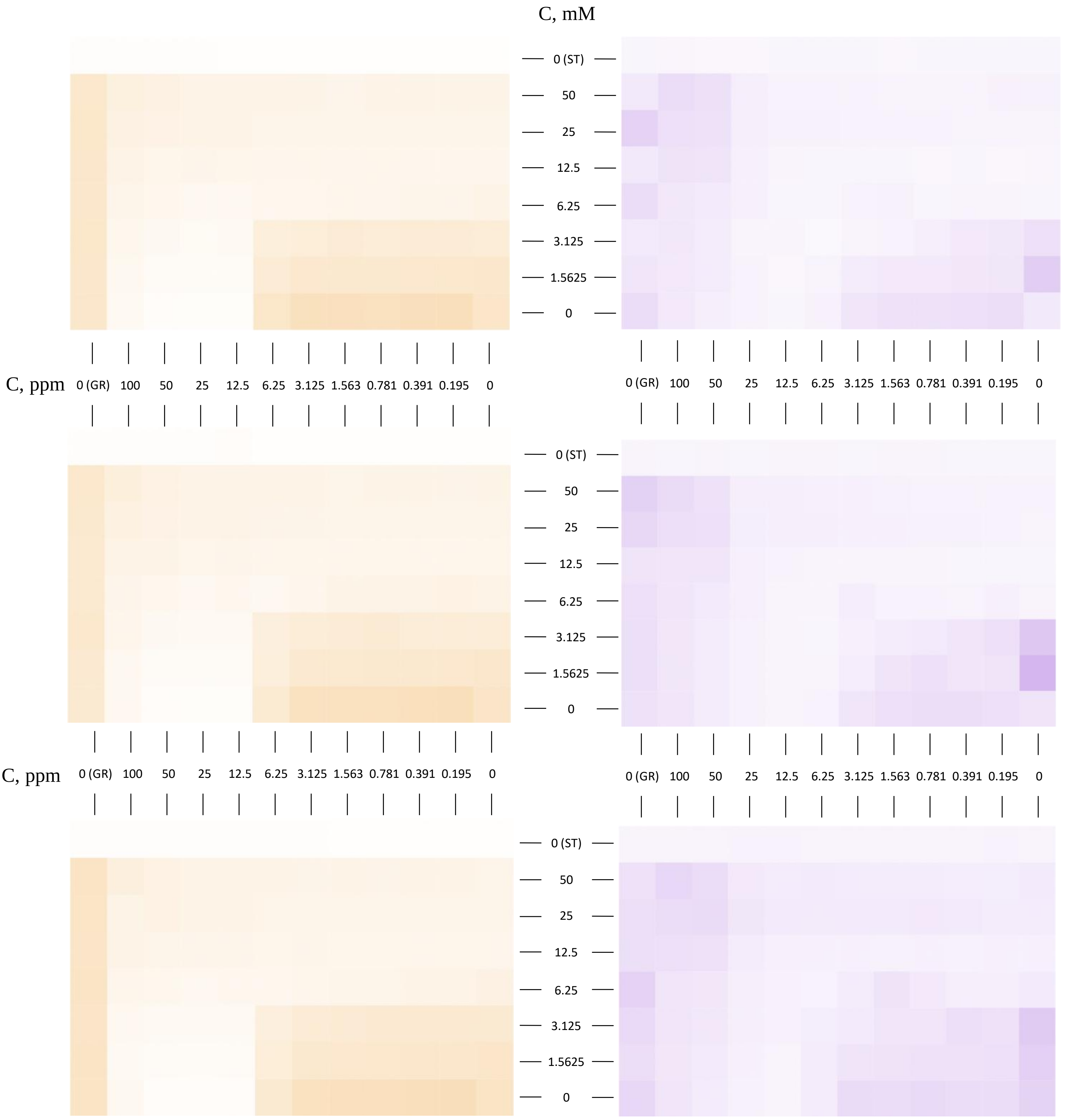

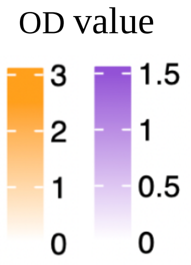

Figure S.2.13. CPB/Cu^{2+}, *E. coli.* Heatmaps of OD readings from the grown plates of planktonic (orange to white) and biofilm (purple to white) growth of *E. coli* after 24h exposure to checkerboard assay of cetylpiridinium bromide (CPB, horizontal concentrations gradient) and copper chloride (Cu^{2+}, vertical concentrations gradient).

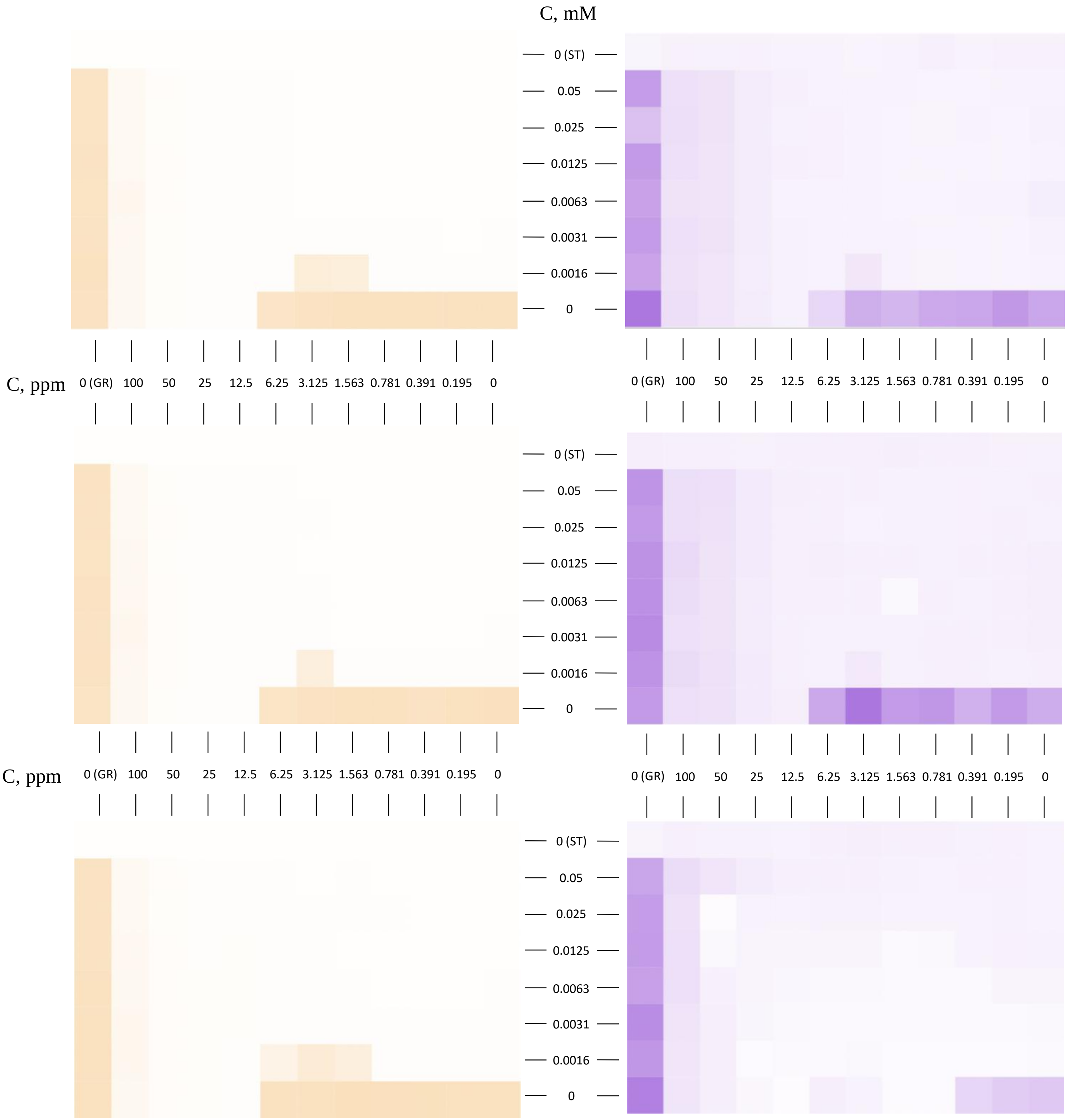

Figure S.2.14. CPB/TeO$_3^{2-}$, *E. coli*. Heatmaps of OD readings from the grown plates of planktonic (orange to white) and biofilm (purple to white) growth of *E. coli* after 24h exposure to checkerboard assay of cetylpiridinium bromide (CPB, horizontal concentrations gradient) and potassium tellurite (TeO$_3^{2-}$, vertical concentrations gradient).

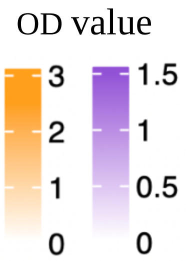

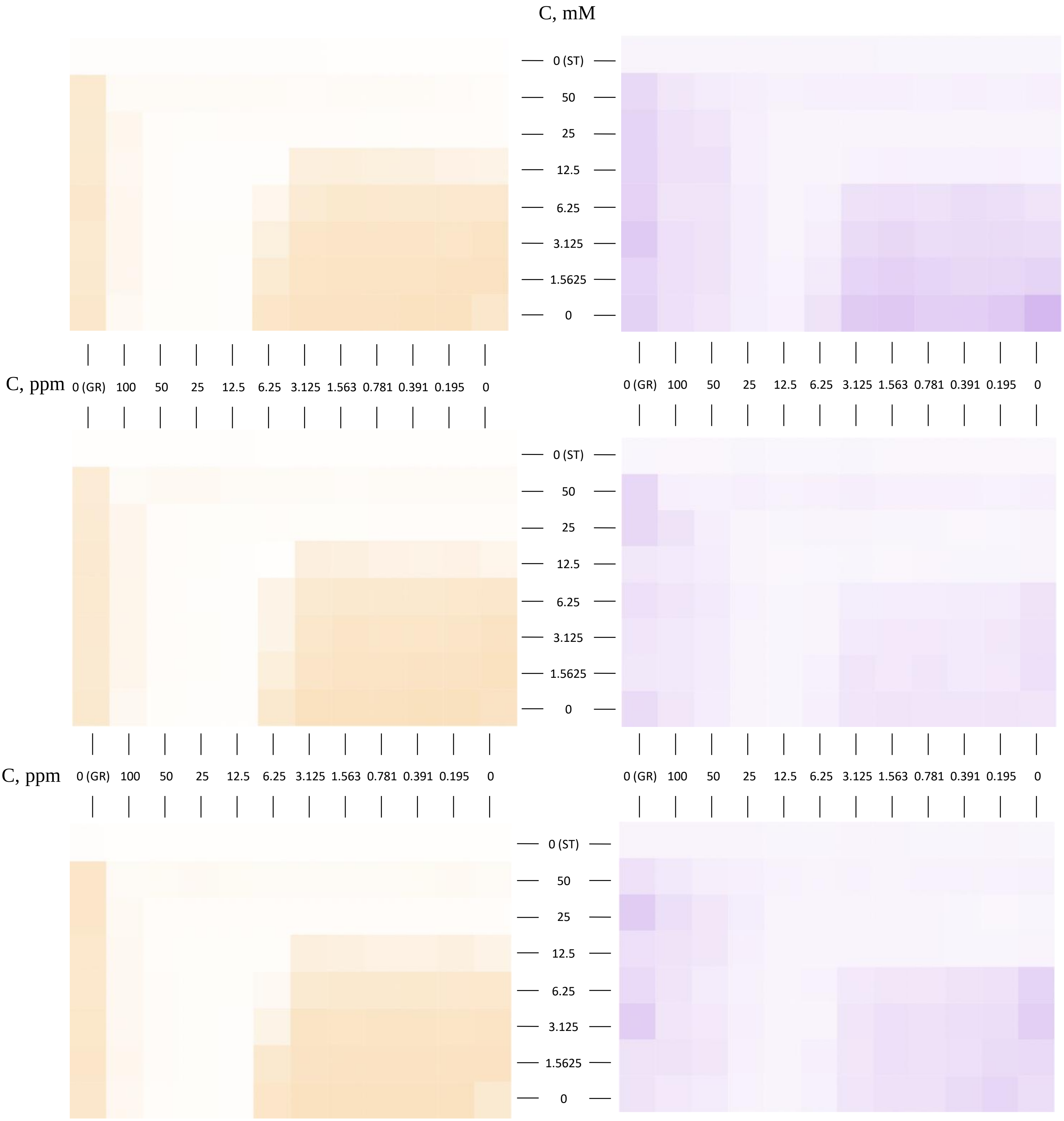

Figure S.2.15. CPB/Zn^{2+}, *E. coli*. Heatmaps of OD readings from the grown plates of planktonic (orange to white) and biofilm (purple to white) growth of *E. coli* after 24h exposure to checkerboard assay of cetylpiridinium bromide (CPB, horizontal concentrations gradient) and zinc chloride (Zn^{2+}, vertical concentrations gradient).

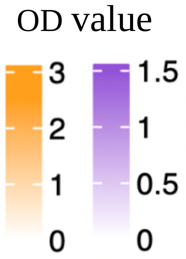

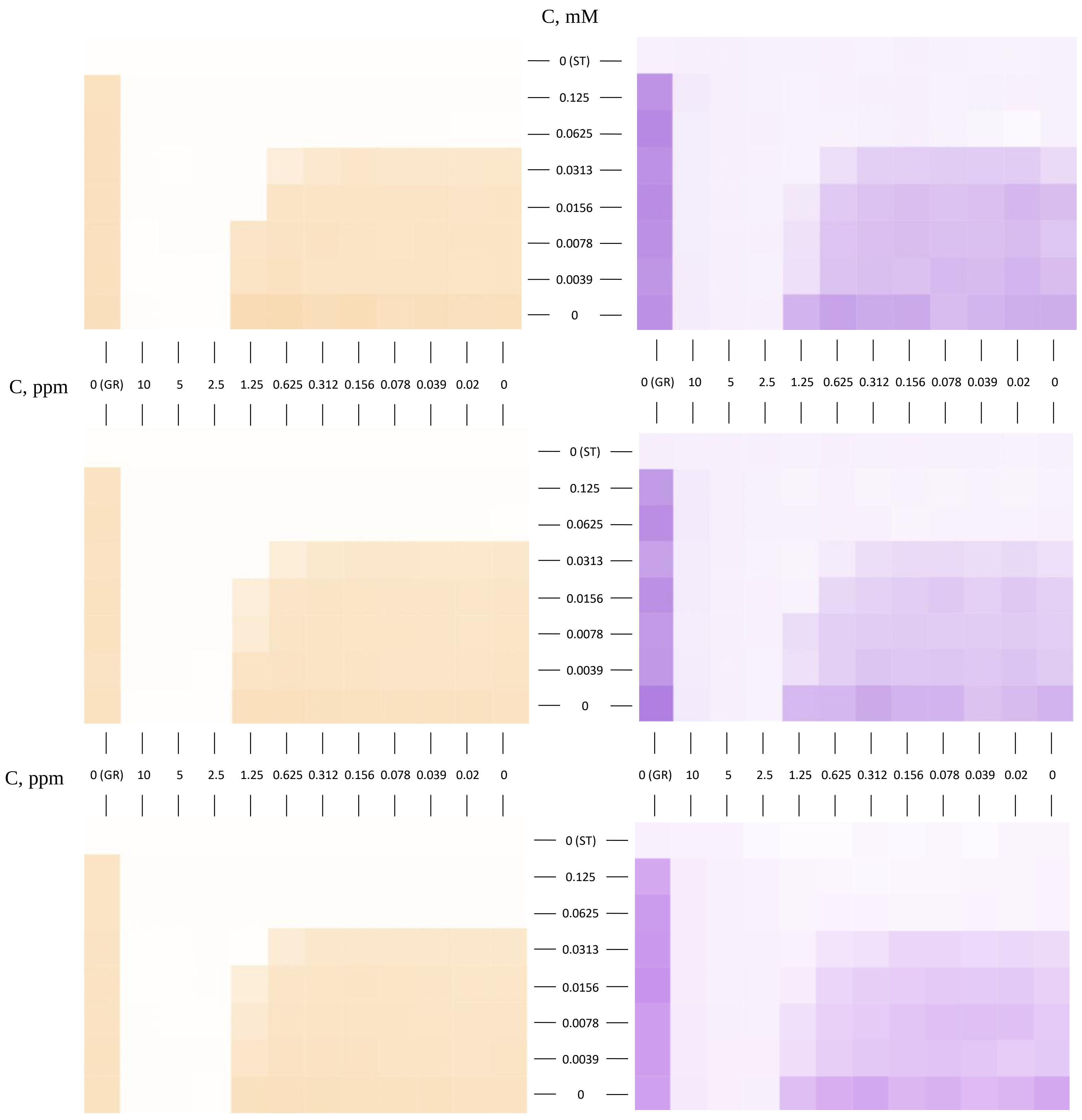

Figure S.2.16. DDAB/Ag$^+$, *E. coli*. Heatmaps of OD readings from the grown plates of planktonic (orange to white) and biofilm (purple to white) growth of *E. coli* after 24h exposure to checkerboard assay of didecyldimethylammonium bromide (DDAB, horizontal concentrations gradient) and silver nitrate (Ag$^+$, vertical concentrations gradient).

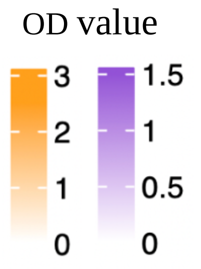

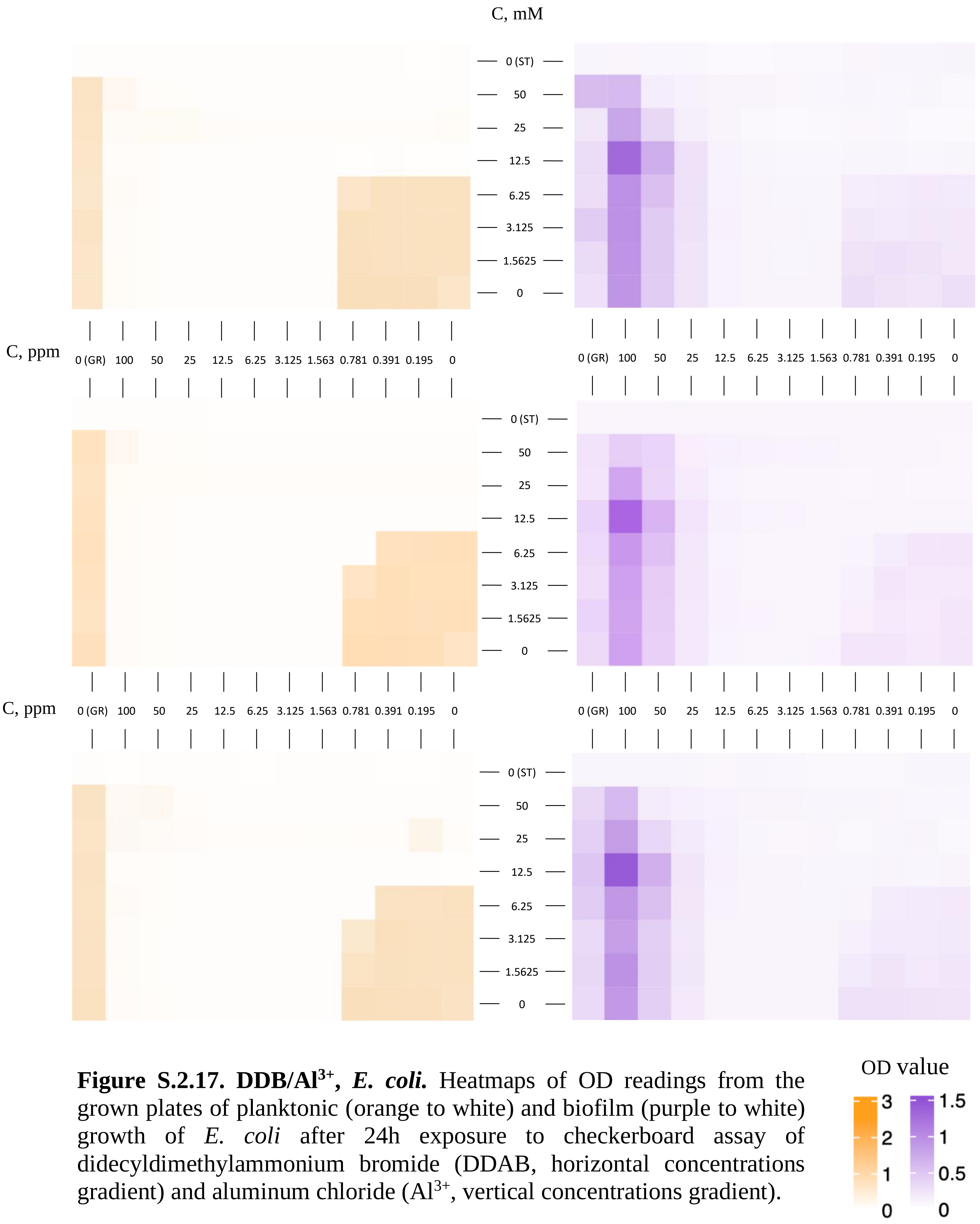

Figure S.2.17. DDB/Al^{3+}, *E. coli*. Heatmaps of OD readings from the grown plates of planktonic (orange to white) and biofilm (purple to white) growth of *E. coli* after 24h exposure to checkerboard assay of didecyldimethylammonium bromide (DDAB, horizontal concentrations gradient) and aluminum chloride (Al^{3+}, vertical concentrations gradient).

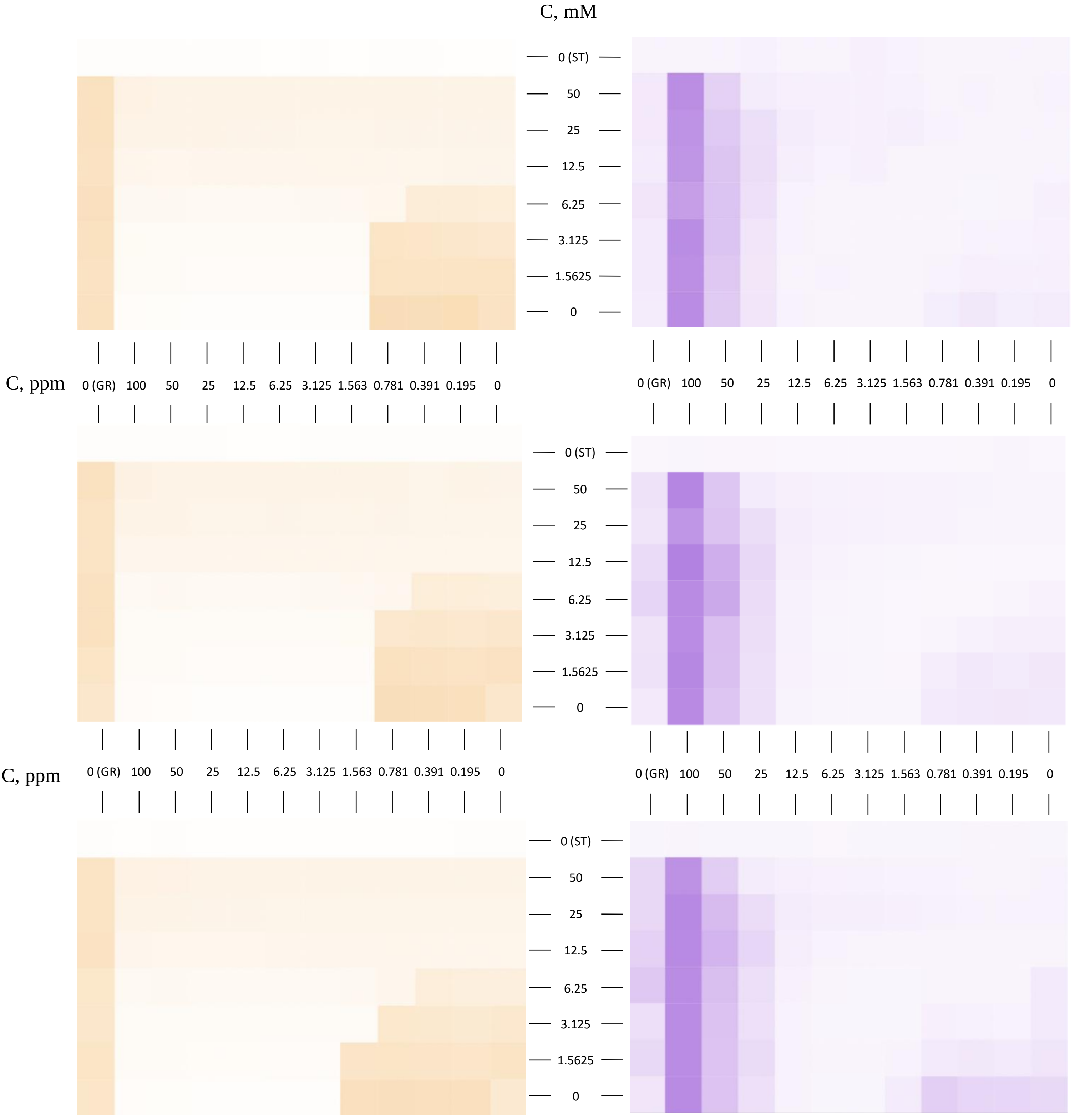

Figure S.2.18. DDAB/Cu^{2+}, *E. coli*. Heatmaps of OD readings from the grown plates of planktonic (orange to white) and biofilm (purple to white) growth of *E. coli* after 24h exposure to checkerboard assay of didecyldimethylammonium bromide (DDAB, horizontal concentrations gradient) and copper chloride (Cu^{2+}, vertical concentrations gradient).

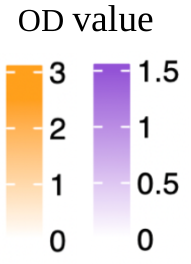

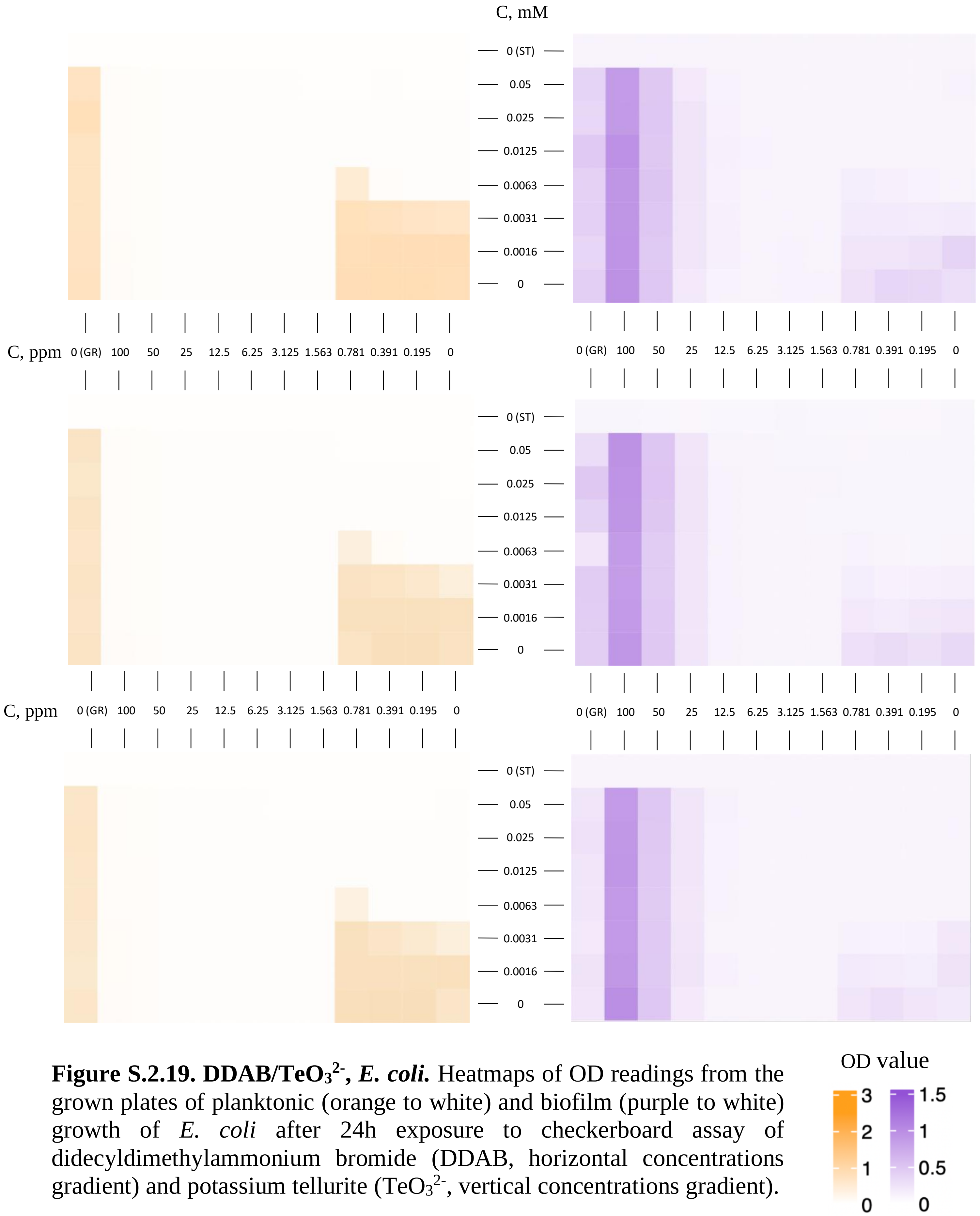

Figure S.2.19. DDAB/TeO_3^{2-}, *E. coli*. Heatmaps of OD readings from the grown plates of planktonic (orange to white) and biofilm (purple to white) growth of *E. coli* after 24h exposure to checkerboard assay of didecyldimethylammonium bromide (DDAB, horizontal concentrations gradient) and potassium tellurite (TeO_3^{2-}, vertical concentrations gradient).

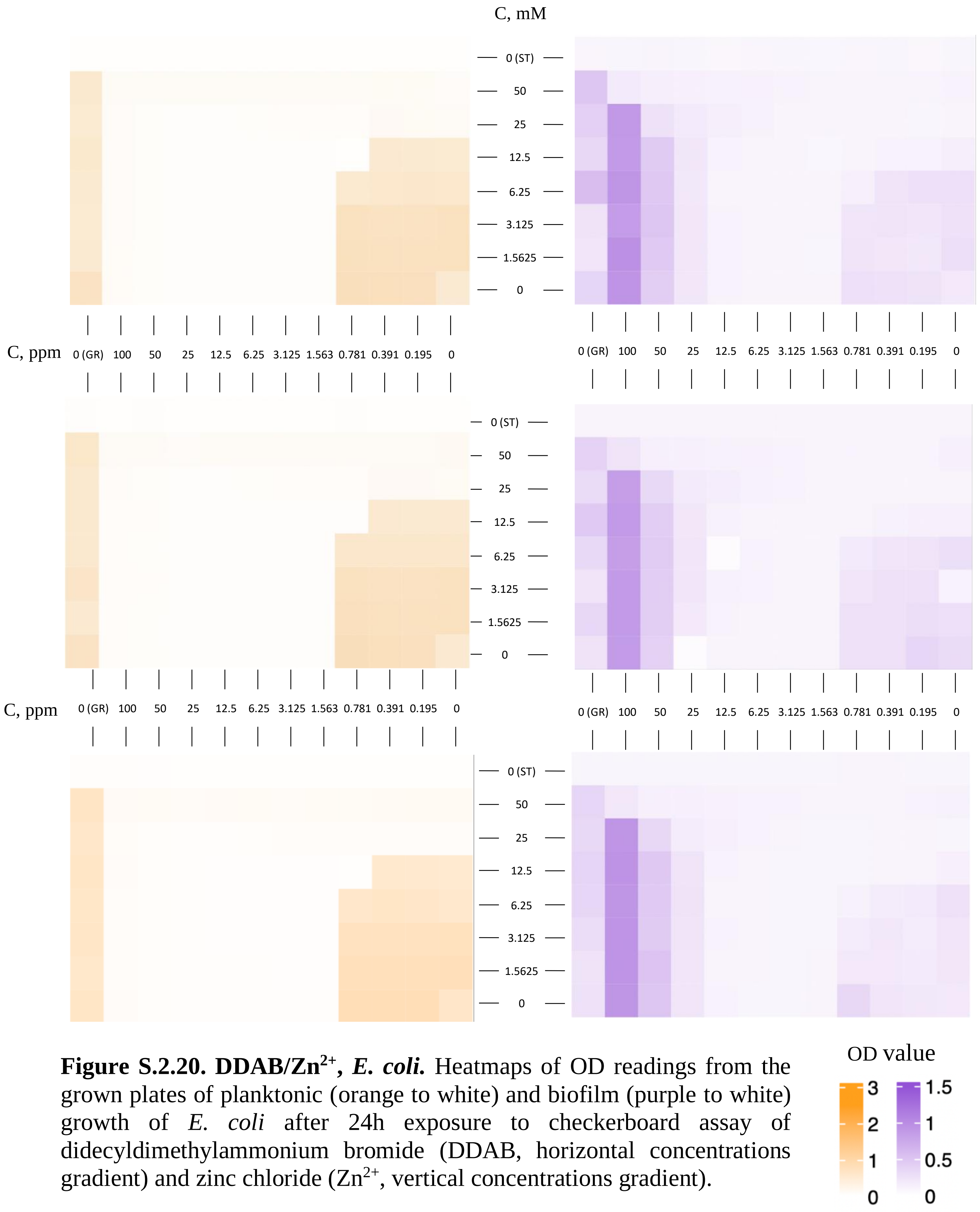

Figure S.2.20. DDAB/Zn^{2+}, *E. coli*. Heatmaps of OD readings from the grown plates of planktonic (orange to white) and biofilm (purple to white) growth of *E. coli* after 24h exposure to checkerboard assay of didecyldimethylammonium bromide (DDAB, horizontal concentrations gradient) and zinc chloride (Zn^{2+}, vertical concentrations gradient).
